# The temporal trend of cause-specific mortality: comparing Estonia and Lithuania, 2001 – 2019

**DOI:** 10.1186/s12889-022-14354-8

**Published:** 2022-10-30

**Authors:** Alexander Tran, Relika Stoppel, Huan Jiang, Kawon Victoria Kim, Shannon Lange, Janina Petkevičienė, Ričardas Radišauskas, Mindaugas Štelemėkas, Tadas Telksnys, Anush Zafar, Jürgen Rehm

**Affiliations:** 1 Institute for Mental Health Policy Research, Centre for Addiction and Mental Health (CAMH), 33 Ursula Franklin Street, Toronto, ON M5S 2S1 Canada; 2 Campbell Family Mental Health Research Institute, CAMH, 250 College Street, Toronto, ON M5T 1R8 Canada; 3 Department of Economics, University of Potsdam, August-Bebel-Straße 89, 14482 Potsdam, Germany; 4 Faculty of Public Health, Health Research Institute, Lithuanian University of Health Sciences, Tilzes Str. 18, 47181 Kaunas, Lithuania; 5 Dalla Lana School of Public Health, University of Toronto, 155 College Street, 6Th Floor, Toronto, ON M5T 3M7 Canada; 6 Department of Psychiatry, University of Toronto, 250 College Street, 8Th Floor, Toronto, ON M5T 1R8 Canada; 7 Department of Preventive Medicine, Faculty of Public Health, Lithuanian University of Health Sciences, Tilzes Str. 18, 47181 Kaunas, Lithuania; 8 Department of Environmental and Occupational Medicine, Faculty of Public Health, Lithuanian University of Health Sciences, Tilžės Str. 18, 47181 Kaunas, Lithuania; 9 Institute of Cardiology, Lithuanian University of Health Sciences, Sukilėlių Str. 15, 50162 Kaunas, Lithuania; 10 Centre for Addiction and Mental Health, World Health Organization / Pan American Health Organization Collaborating Centre, 33 Ursula Franklin Street, Toronto, ON M5S 2S1 Canada; 11 Faculty of Medicine, Institute of Medical Science, University of Toronto, Medical Sciences Building, 1 King’s College Circle, Room 2374, Toronto, ON M5S 1A8 Canada; 12 Institute of Clinical Psychology and Psychotherapy & Center of Clinical Epidemiology and Longitudinal Studies (CELOS), Technische Universität Dresden, Chemnitzer Str. 46, 01187 Dresden, Germany; 13 Department of International Health Projects, Institute for Leadership and Health Management, I.M. Sechenov First Moscow State Medical University, Trubetskaya Str., 8, B. 2, 119992 Moscow, Russian Federation; 14 Program On Substance Abuse & Designated WHO CC, Public Health Agency of Catalonia, 81-95 Roc Boronat St, 08005 Barcelona, Spain

**Keywords:** Epidemiological transition, Temporal trends, Cause-specific mortality, Joinpoint regression

## Abstract

**Background:**

Despite being two Baltic countries with similar histories, Estonia and Lithuania have diverged in life expectancy trends in recent years. We investigated this divergence by comparing cause-specific mortality trends.

**Methods:**

We obtained yearly mortality data for individuals 20 + years of age from 2001–2019 (19 years worth of data) through Statistics Lithuania, the Lithuanian Institute for Hygiene, and the National Institute for Health Development (Estonia). Using ICD-10 codes, we analyzed all-cause mortality rates and created eight major disease categories: ischemic heart disease, cerebrovascular disease, all other cardiovascular disease, cancers (neoplasms), digestive diseases, self-harm and interpersonal violence, unintentional injuries and related conditions, and other mortality (deaths per 100,000 population). We used joinpoint regression analysis, and analyzed the proportional contribution of each category to all-cause mortality.

**Results:**

There was a steeper decline in all-cause mortality in Estonia (average annual percent change, AAPC = -2.55%, 95% CI: [-2.91%, -2.20%], *P* < .001) as compared to Lithuania (AAPC = -1.26%, 95% CI: [-2.18%, -0.57%], *P* = .001). For ischemic heart disease mortality Estonia exhibited a relatively larger decline over the 19-year period (AAPC = -6.61%, 95% CI: [-7.02%, -6.21%], *P* < .001) as compared to Lithuania (AAPC = -2.23%, 95% CI: [-3.40%, -1.04%], *P* < .001).

**Conclusion:**

Estonia and Lithuania showed distinct mortality trends and distributions of major disease categories. Our findings highlight the role of ischemic heart disease mortality. Differences in public health care, management and prevention of ischemic heart disease, alcohol control policies may explain these differences.

**Supplementary Information:**

The online version contains supplementary material available at 10.1186/s12889-022-14354-8.

## Introduction

Epidemiologic transition refers to a temporal change in population-level patterns of health and disease (e.g., fertility, life expectancy, mortality, and leading causes of death) and its interaction with demographics, and sociologic and economic determinants [1]. As countries undergo development and transition there is gradual shift from high infant mortality rates, low life expectancy, and predominantly infectious diseases, to low infant mortality, increasing life expectancy, and a higher prevalence of degenerative and/or man-made diseases [2]. Epidemiological transitions become more nuanced over time [3–6]. Notably the reductions in the prevalence of communicable diseases leads to an increase in chronic, degenerative man-made disease [1], however the relative proportion of each type of chronic diseases does not remain static. Life expectancy gains in Western European countries (e.g., Germany) are attributed to reductions in the burden of CVD, accompanied by an increase in the relative prevalence of cancers [7]. Analyzing the specific contribution of disease categories in a country can identify target areas for public policy, reduce all-cause mortality by addressing the largest burdens of disease, and subsequently lead to better socioeconomic development [6, 8].

Cardiovascular disease (CVD) has been considered a marker for achieving improved socioeconomic outcomes and higher life expectancy in Russia and surrounding countries. A shift in the burden of different types of chronic disease, however, may indicate a fourth epidemiological transition. For instance, the decline in CVD in Estonia as compared to Russia, is cited as a contributing factor to the subsequent decrease in all-cause mortality [9, 10]. Importantly, there is some indication that alcohol is an underlying factor [11, 12]. In the countries that were formerly a part of the Soviet Union, the high adult mortality rate has been attributed to lifestyle factors (e.g., heavy binge drinking [6, 13]) and has distinguished these countries from other high-income European countries [8].

**Fig. 1:**
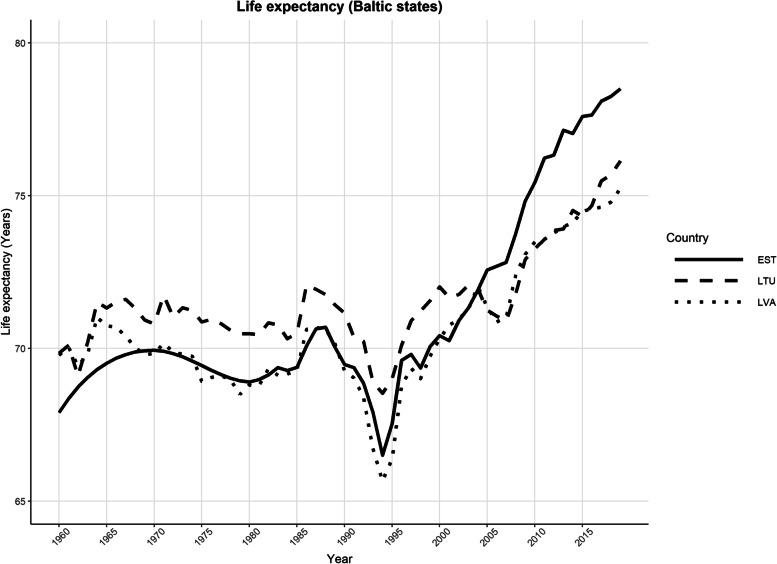
Comparison of life expectancy between the Baltic states (EST = Estonia, LTU = Lithuania, LVA = Latvia) between 1960 and 2019. Data obtained from The World Bank [19]

In terms of epidemiological transition theory, the Soviet Union (and after its dissolution, Russia and the surrounding countries) emerged as a unique case. There was a substantial reduction in maternal and early childhood mortality rates in the Soviet Union and a decline in infectious diseases, however the life expectancy began to stagnate in the 1970s and 1980s (see Fig. 1). The end of the Soviet Union in the 1990s was followed by diverging trends (due to political reform and health policy changes) between countries, once they regained their independence [12, 14]. Evidently the mortality trends of these countries form a cluster; according to the Global Burden of Disease Study in 2019, there is a distinct pattern of low childhood and high adult mortality in the former Soviet Union countries in Central Europe which is distinct from other neighbouring countries [8, 15]. Estonia and Lithuania were part of the Soviet Union, until the restoration of their formal independence in 1991 (de facto in 1990), and these two countries share some sociocultural characteristics. Both countries have a culture of heavy drinking including heavy episodic drinking, which may have contributed to relatively higher liver cirrhosis mortality rates compared to some other European countries [13, 16–18].

Since the fall of the Soviet Union, both countries demonstrated similar patterns, overall, in life expectancy, which can be characterized by an initial decline in the early 1990s, followed by an increase until the years of 2000–2005. The patterns soon diverged in the subsequent years; in Lithuania there was a decline in life expectancy until 2008, followed by a slower acceleration, whereas Estonia increased in life expectancy from 2005 onward [20]. Thus, although both countries were able to regain independence and were ushered into the European Union at the same time, in May 2004, life expectancy in the years that followed differed quite dramatically. Estonia achieved a stable increase in life expectancy earlier, compared to Lithuania [9, 20]. Noteworthy, is the fact that when Estonia regained its independence, it had a *lower life expectancy* relative to Lithuania; however, over the subsequent years (1990s – mid 2000s) Estonia began to consistently trend upward in life expectancy, surpassing Lithuania (although in recent years the gap has begun to narrow). In Lithuania, life expectancy rose in the 1990s before stagnating through the mid 2000s, dropping between 2005 and 2007, then regaining an upward trend [20].

To further explore the distinct patterns between Estonia and Lithuania, this article analyzes the different trajectories of mortality rates in these two separate, but similar countries. We investigate the differences in specific causes of death and their relative contributions to all-cause mortality, we analyze the temporal trends in all-cause mortality between these countries, and analyze the change in temporal trends of cardiovascular diseases, a leading cause of death in Eastern European countries [11]. Decomposing the temporal trends in all-cause mortality and cause-specific contributions may identify where there are distinct mortality trends between these two countries and help to inform policy makers on key areas to improve life expectancy in these countries.

## Methods

### Data

For Lithuania and Estonia, we obtained data for the number of cause-specific deaths per year separated by 5-year age groups (0–4, 5–9, 10–14, 15–19,…,85 +) and by sex from 2001 to 2019 (19 years worth of data). Access to the data was granted through Statistics Lithuania and the Lithuanian Institute of Hygiene, and from the National Institute for Health Development (Estonia) respectively [21–23]. The specific causes of death were coded as per the International Classification of Diseases (ICD-10, [24]). The number of deaths resulting from individual causes were then grouped into eight unique pre-determined categories: ischemic heart disease, cerebrovascular disease, neoplasms, digestive diseases, all other cardiovascular disease (other CVD), self-harm and interpersonal violence, unintentional injuries and related conditions, and other causes of death (see Table S1 in the Supplementary Materials for each category and the corresponding ICD-10 codes). The data sources included only country residents in their mortality counts, with only a small fraction of deaths outside of the country recorded by each registry (0.1% of deaths in Estonia were residents abroad, and 1.2% in Lithuania). and These categories were also in line with that of the Institute for Health Metrics and Evaluation (IHME) and the Global Burden of Disease (GBD) Study [8, 25]. Five of these categories were identified in the GBD as among the highest cause-specific mortality rates for non-communicable diseases (NCDs, [26]).

### Dependent variable

The dependent variable was yearly age-standardized mortality rate per 100,000 people for individuals 20 + years of age (using the European Standard, [27]) for males, females and both sexes combined. We computed mortality rates for the eight cause-specific categories. In a sensitivity analysis, we also standardized mortality rates using the World Health Organization standard (see Supplementary Materials). Given that the Lithuania data were provided as monthly data, we aggregated the deaths into yearly values in order to compare across countries.

### Statistical analyses

We first tested a general linear trend for all-cause mortality in each country using a joinpoint regression analysis. The joinpoint regression analysis is a statistical procedure that employs a permutation method to determine inflection points from time series data and identifies linear trends in the data. It iteratively analyzes the data to determine the best fit for a set of linear segments determined by a pre-determined maximum number of inflection points (joinpoints). The maximum number of joinpoints for our analyses was set to three (as is recommended for 17–21 data points, [28]). As well, the analysis computes an annual percentage change (APC) for each linear segment, which is the slope of the segment, or change in rate for each year. It also computes an overall average annual percentage change (AAPC), which is the APC, averaged across all linear segments of the time series. The joinpoint analysis was performed using the Joinpoint Regression Program, version 4.9.0.1 [29]. We also decomposed all-cause mortality into specific causes, and performed separate joinpoint regression analyses for all eight major disease categories to identify time periods where there was a significant change in the slope, as well as AAPC for cause-specific mortality rates.

We compared each country’s all-cause mortality rate at the beginning of the dataset (2001), the mid-point of the dataset (2010) and the final data point (2019), and measured the relative contribution of each cause-specific mortality rate to identify how the proportion of these causes changed between each of these time points for each country respectively. These additional analyses were performed in R version 4.0.4 [30].

## Results

**Fig. 2:**
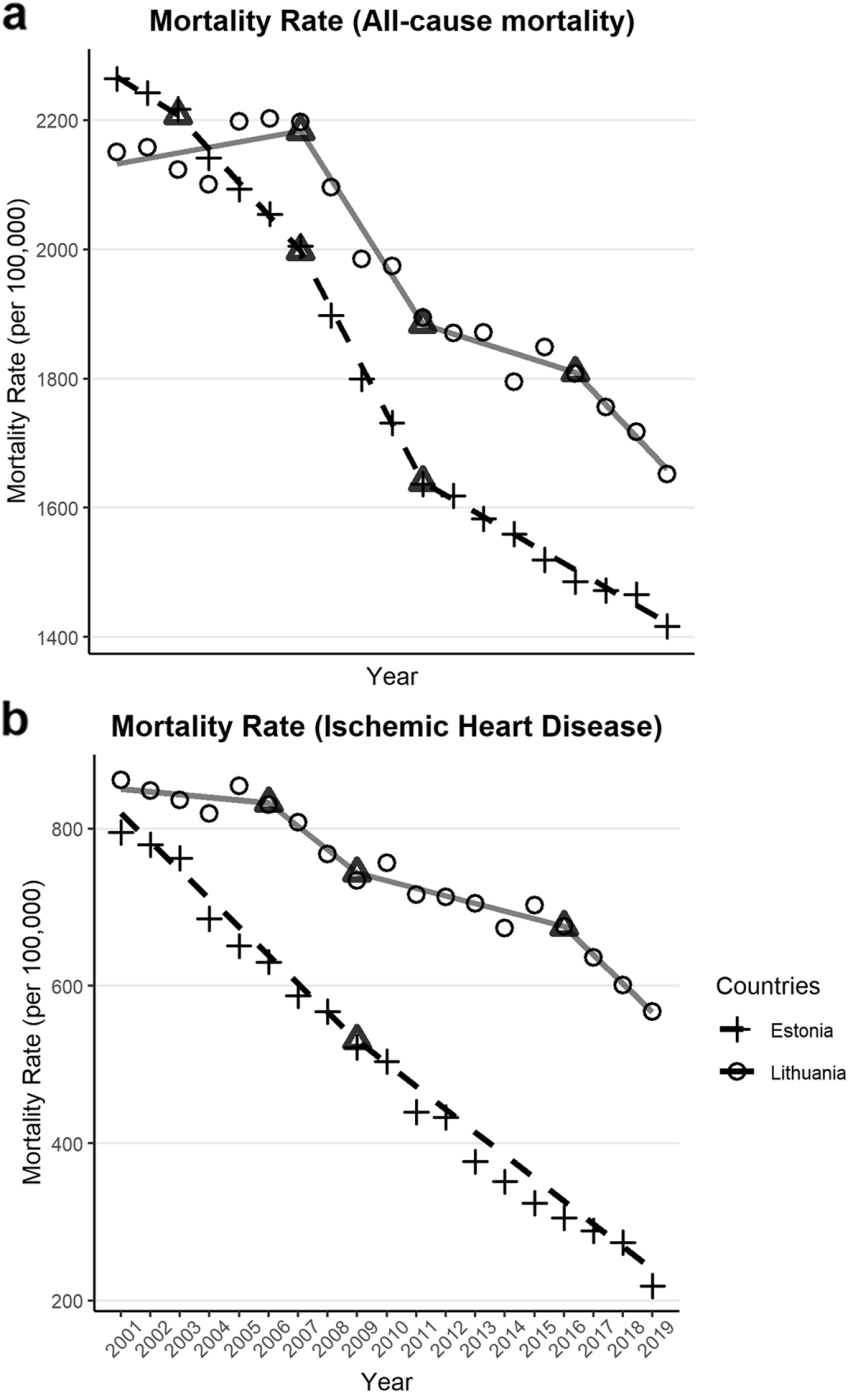
Joinpoint analysis of all-cause (Graph a) and ischemic heart disease (Graph b) mortality rates (deaths per 100,000 people, 20 + years of age) for Estonia (dashed) and Lithuania (solid). Raw data shown by points, joinpoint trends shown by lines, with joinpoints identified are enclosed by triangles

For both sexes combined, as per the joinpoint regression analyses, Estonia had a larger AAPC decrease in all-cause mortality (AAPC = -2.55%, 95% CI: [-2.91%, -2.20%], *P* < 0.001) during the study period (2001–2019) than Lithuania (AAPC = -1.26%, 95% CI: [-2.18%, -0.57%], *P* = 0.001) (See Fig. 2). In both Estonia and Lithuania, three joinpoints were identified; in Estonia, the first joinpoint was in 2003, 95% CI: [2003, 2008] and the second was in 2007, 95% CI: [2006, 2012] and the final point was in 2011, 95% CI: [2010, 2017]. In Lithuania, the first joinpoint was in 2007, 95% CI: [2003, 2009] and the second was in 2011, 95% CI: [2006, 2014] and the final point was in 2016, 95% CI: [2009, 2017]. Specifically, in Estonia the slopes of three of the four segments in all-cause mortality were negative, with a *p*-value of *P* < 0.05, which were considered statistically significant. Meanwhile in Lithuania, only the second and the final segment had a decline with a *P* < 0.05 (the other two segments did not pass the *P* < 0.05 threshold; see Table 1 for full statistics of APC for each segment). When separated by sex, the all-cause mortality trend in Estonia was better approximated by females with 3 joinpoints (see Fig. 5) and significant declines in the latter 3 segments (see Table 3). In contrast, the all-cause mortality trend in Lithuania was better approximated by male mortality, demonstrating the same 3 joinpoints (see Fig. 5), but notably there was a significant increase in mortality in the first segment (APC = 0.84%, 95% CI: [0.11%, 1.58%], *P* < 0.03).

**Table 1:** Annual percentage change in mortality rate from joinpoint analysis (both sexes)

	Time period	APC	95% CI	*p*-value
All-cause mortality				
Estonia				
Segment 1	2001 – 2003	-1.32	[-3.54, 0.95]	*P* = .21
Segment 2	2003 – 2007	-2.47	[-3.51, -1.42]	*P* < .001
Segment 3	2007 – 2011	-4.81	[-5.82, -3.79]	*P* < .001
Segment 4	2011 – 2019	-1.77	[-1.98, -1.55]	*P* < .001
Total AAPC		-2.55	[-2.91, -2.20]	*P* < .001
Lithuania				
Segment 1	2001 – 2007	0.39	[-0.49, 1.29]	*P* = .34
Segment 2	2007 – 2011	-3.61	[-6.29, -0.85]	*P* = .02
Segment 3	2011 – 2016	-0.80	[-2.49, 0.91]	*P* = .31
Segment 4	2016 – 2019	-2.85	[-5.58, -0.05]	*P* = .05
Total AAPC		-1.26	[-2.18, -0.57]	*P* = .001
Ischemic heart disease mortality				
Estonia				
Segment 1	2001 – 2009	-5.28	[-6.01, -4.51]	*P* < .001
Segment 2	2009 – 2019	-7.66	[-8.18, -7.14]	*P* < .001
Total AAPC		-6.61	[-7.02, -6.21]	*P* < .001
Lithuania				
Segment 1	2001 – 2006	-.42	[-1.60, 0.78]	*P* = .44
Segment 2	2006 – 2009	-3.69	[-10.59, 3.75]	*P* = .28
Segment 3	2009 – 2016	-1.37	[-2.21, -0.52]	*P* = .006
Segment 4	2016 – 2019	-5.68	[-8.55, -2.73]	*P* = .002
Total AAPC		-2.23	[-3.40, -1.04]	*P* < .001

*Note*: *APC* Annual percentage change, *AAPC* Average annual percentage change

In addition, we performed a joinpoint analysis on ischemic heart disease mortality rates and also found that there was a steeper overall decline in Estonia (AAPC = -6.61%, 95% CI: [-7.02%, -6.21%], *P* < 0.001]) than Lithuania (AAPC = -2.23%, 95% CI [-3.40%, -1.04%], *P* < 0.001, see Fig. 2. For ischemic heart disease, in Estonia there was only one joinpoint, whereas in Lithuania there were three joinpoints. In Estonia, the joinpoint was in 2009, 95% CI: [2007, 2011] and in Lithuania, the joinpoints were in 2006, 95% CI: [2006, 2008], in 2009, 95% CI: [2007, 2014], and in 2016, 95% CI: [2014, 2017]. In Estonia, both segments showed a decline in ischemic heart disease mortality which were considered statistically significant at *P* < 0.05, with an acceleration in slope in the second segment, while in Lithuania, the first two segments were not significant, however the decline in ischemic heart disease mortality from 2009 onward was considered significant at *P* < 0.05 (see Table 2 for model selection based on comparisons with different joinpoints using Bayesian information criterion). When separated by sex, the IHD trend in Estonia was similar for both males and females, with only a single joinpoint (see Fig. 5 and Table 3). Meanwhile the IHD trend in Lithuania was better approximated by female mortality, demonstrating 3 joinpoints, of which only the final segment had a significant decline.

**Table 2:**
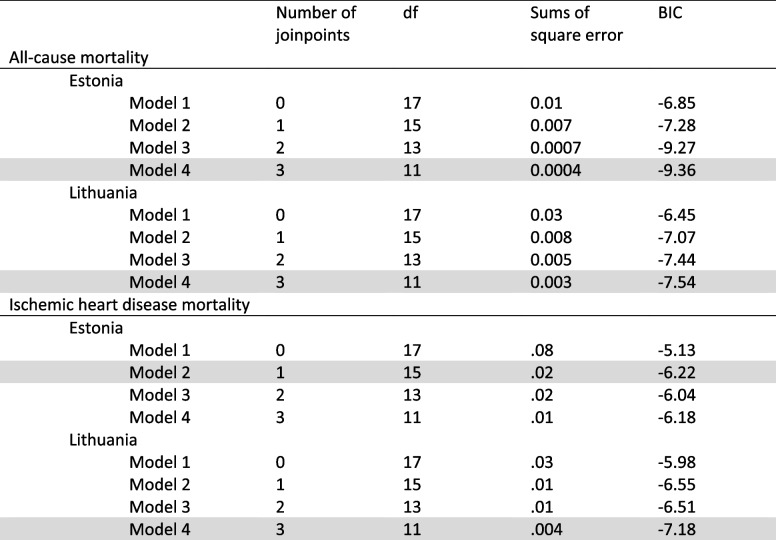
Model selection via Bayesian Information Criterion (BIC), joinpoint regression analysis

*Note*: Final models selected are highlighted in grey. *BIC* Bayesian information criterion

**Table 3:** Annual percentage change in mortality rate from joinpoint analysis (sex-specific)

Males				
	Time period	APC	95% CI	*p*-value
All-cause mortality				
Estonia				
Segment 1	2001 – 2007	-1.69	[-3.54, 0.95]	*P* < .001
Segment 2	2007 – 2011	-5.09	[-3.51, -1.42]	*P* < .001
Segment 3	2011 – 2019	-2.04	[-2.27, -1.08]	*P* < .001
Total AAPC		-2.61	[-2.90, -2.32]	*P* < .001
Lithuania				
Segment 1	2001 – 2007	0.84	[0.11, 1.58]	*P* = .03
Segment 2	2007 – 2010	-3.48	[-5.75, -1.14]	*P* = .01
Segment 3	2010 – 2019	-0.97	[-2.38, 0.45]	*P* = .15
Segment 4	2016 – 2019	-3.04	[-5.37, -0.66]	*P* = .02
Total AAPC		-1.28	[-1.97, -0.60]	*P* < .001
Ischemic heart disease mortality				
Estonia				
Segment 1	2001 – 2008	-4.78	[-5.68, -3.88]	*P* < .001
Segment 2	2008 – 2019	-7.26	[-7.67, -6.83]	*P* < .001
Total AAPC		-6.30	[-6.69, -5.90]	*P* < .001
Lithuania				
Segment 1	2001 – 2005	-.32	[-1.78, 1.16]	*P* = .64
Segment 2	2005 – 2016	-1.85	[-2.16, -1.55]	*P* < .001
Segment 3	2016 – 2019	-5.12	[-7.52, -2.67]	*P* < .001
Total AAPC		-2.07	[-2.57, -1.57]	*P* < .001
Females				
	Time period	APC	95% CI	*p*-value
All-cause mortality				
Estonia				
Segment 1	2001 – 2003	-1.03	[-3.75, 1.76]	*P* = .41
Segment 2	2003 – 2008	-3.30	[-3.30, -4.01]	*P* < .001
Segment 3	2008 – 2011	-4.84	[-4.84, -7.42]	*P* < .001
Segment 4	2011 – 2019	-1.64	[-1.64, -1.87]	*P* = .003
Total AAPC		-2.58	[-3.07, -2.08]	*P* < .001
Lithuania				
Segment 1	2001 – 2007	-0.22	[-1.08, 0.64]	*P* = .58
Segment 2	2007 – 2010	-3.75	[-9.93, 2.84]	*P* = .23
Segment 3	2010 – 2019	-1.29	[-2.49, 0.91]	*P* < .001
Total AAPC		-1.35	[-2.37, -0.32]	*P* = .01
Ischemic heart disease mortality				
Estonia				
Segment 1	2001 – 2009	-5.33	[-6.33, -4.32]	*P* < .001
Segment 2	2009 – 2019	-8.33	[-9.01, -7.65]	*P* < .001
Total AAPC		-7.01	[-7.54, -6.47]	*P* < .001
Lithuania				
Segment 1	2001 – 2006	-0.76	[-2.28, 0.77]	*P* = .28
Segment 2	2006 – 2009	-4.10	[-12.80, 5.47]	*P* = .34
Segment 3	2009 – 2016	-1.29	[-2.38, -0.19]	*P* = .02
Segment 4	2016 – 2019	-5.75	[-9.42, -1.94]	*P* = .009
Total AAPC		-2.37	[-3.87, -0.85]	*P* = .002

*Note*: *APC* Annual percentage change, *AAPC* Average annual percentage change

**Fig. 3:**
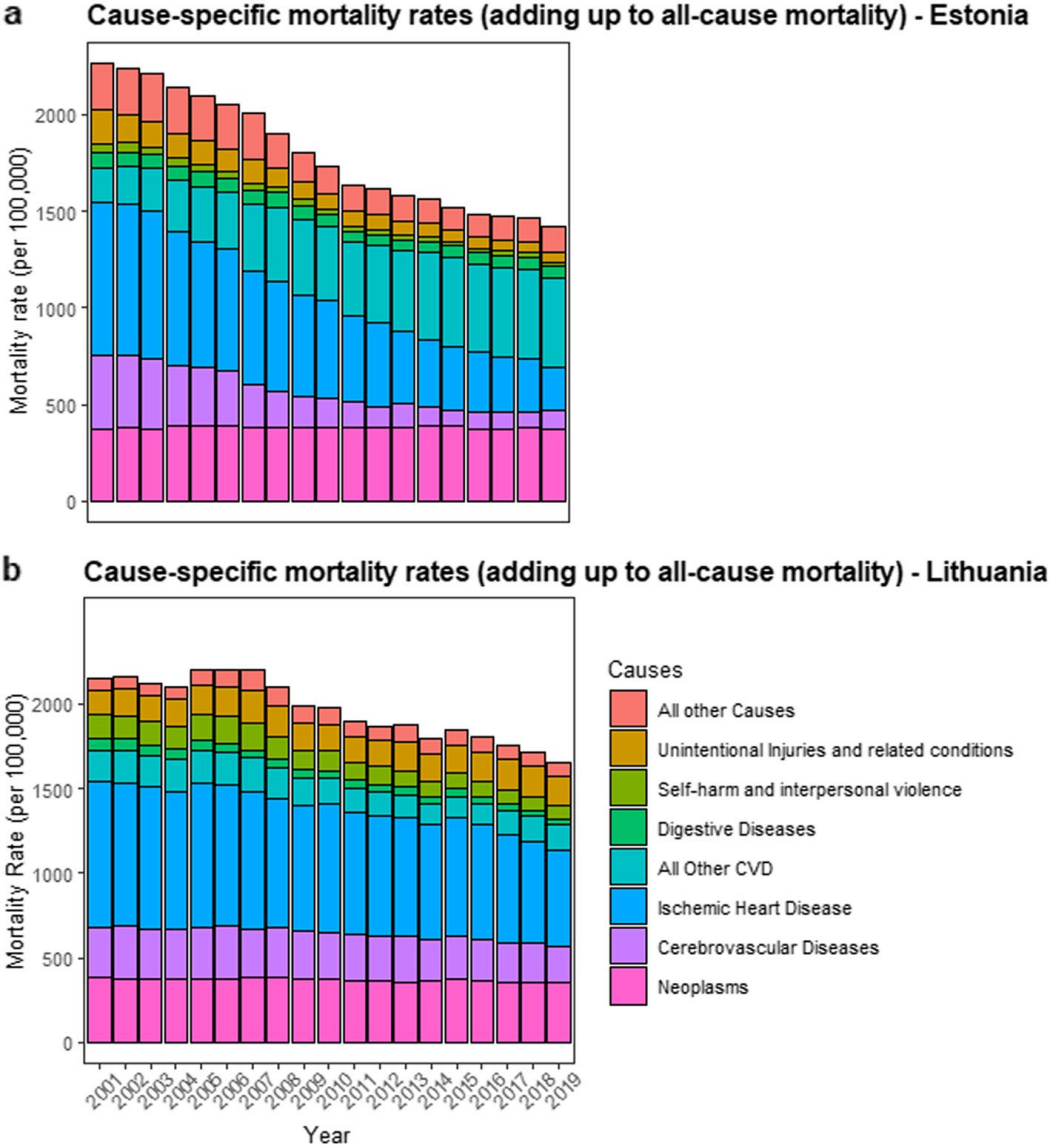
Mortality rate (deaths per 100,000 individuals, 20 + years of age) in Estonia (Graph a) and Lithuania (Graph b), separated by causes between 2001 and 2019. Mortality rate in each year across all categories adds up to all-cause mortality

**Fig. 4:**
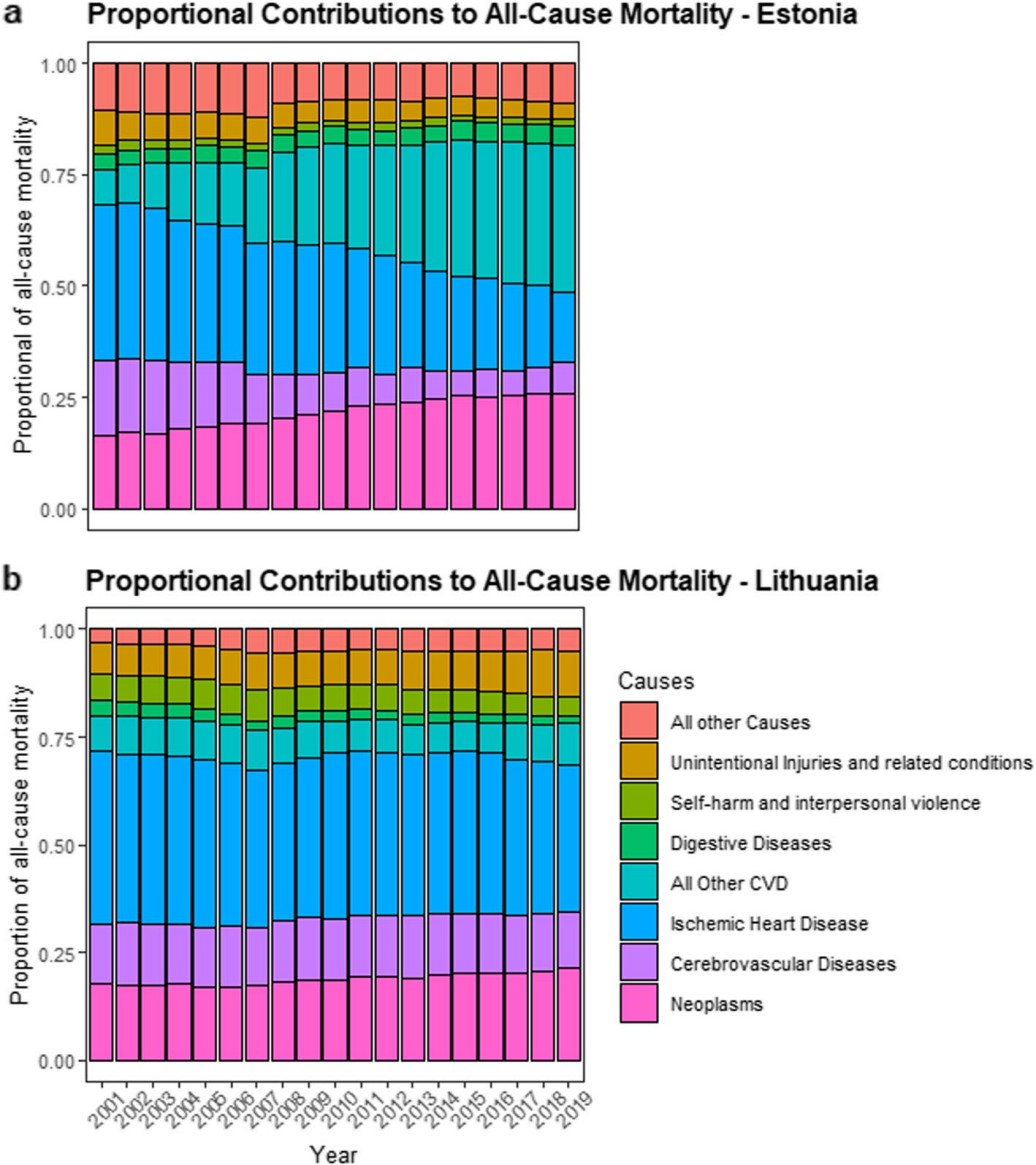
Proportional contributions of each cause-specific category to all-cause mortality rate (deaths per 100,000 individuals, 20 + years of age) in Estonia (Graph a) and Lithuania (Graph b). For each year the mortality rate is divided into the individual cause-specific categories, and the categories are shown as a percentage of the all-cause mortality rate

**Fig. 5:**
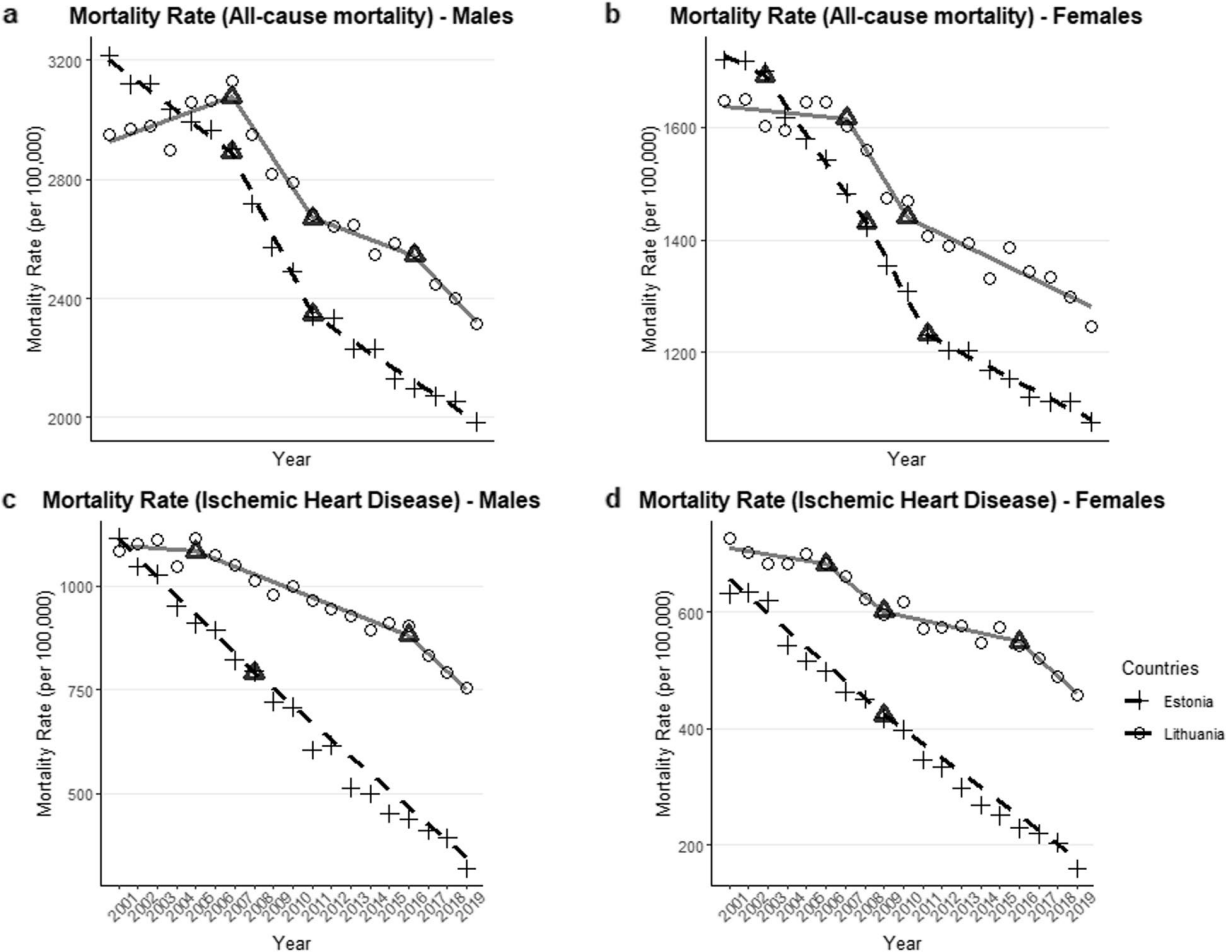
Joinpoint analysis of all-cause (Graphs a and b) and ischemic heart disease (Graphs c and d) mortality rates (deaths per 100,000 people, 20 + years of age) for Estonia (dashed) and Lithuania (solid), separated by sex. Raw data shown by points, joinpoint trends shown by lines, with joinpoints identified are enclosed by triangles

When we decomposed all-cause mortality rates into the eight major disease categories, in Estonia, the largest contributors were ischemic heart disease (between 15.4% and 35.1%), all other cardiovascular diseases (between 7.9% and 33.1%), and neoplasms (between 16.4% and 25.7%, see Supplementary Table 2, and Figs. 3 and 4). In Lithuania, the causes of death that contributed the most to all-cause mortality across the 19-year period were, ischemic heart disease (between 34.3% and 40.1%), followed by neoplasms (between 17.2% and 23.7%), and cerebrovascular diseases (between 12.8% and 14.5%, see Supplementary Table 3, Figs. 3 and 4). The joinpoint regression findings, for both sexes combined, are shown in the main text, with analyses for all-cause mortality and ischemic heart disease are presented in Figs. 2, 3 and for individual sexes in Fig. 5. For all other categories, and sex specific analyses, see the Supplementary Materials.

For the three time points of interest, (the beginning of the study period: 2001, the mid-point: 2010 and the final data point: 2019), in Estonia, the most notable changes were an increase in the proportion of cancer (neoplasm) mortality rate; 16.4% (2001), 21.8% (2010), 25.7% (2019), as well an increase in all other cardiovascular diseases; 7.9% (2001), 22.4% (2010), 33.1% (2019). As well, there was a substantial decrease in ischemic heart disease; 35.1% (2001), 29.1% (2010), 15.4% (2019). Changes in the proportion of other causes for Estonia can be found in Supplementary Table 2. In Lithuania, the relative proportion of each cause remained largely stable, however there was a trend of increasing proportion of cancer (neoplasm) mortality; 17.8% (2001), 18.8% (2010), 23.7% (2019), and other mortality rate; 7% (2001), 7.8% (2010), 10.5% (2019). As well there was a slight decrease in ischemic heart disease; 40.1% (2001), 38.3% (2010), 34.3% (2019). All other changes can be found in Supplementary Table 3.

## Discussion

Our findings demonstrate nuanced differences in all-cause mortality and cause-specific mortality trends between two Baltic countries. Countries of the former Soviet Union exhibit a unique life expectancy profile, associated with worse health outcomes as compared to other European countries [8]. Despite some similarities, there is evidence of an earlier epidemiological transition in Estonia than Lithuania (e.g., cardiovascular revolution [6]). In Estonia, there was a steeper decline in all-cause mortality, and in ischemic heart disease (although there was an increase in deaths due to other cardiovascular diseases, it was less than that decline in ischemic heart disease, see Supplementary Materials). Noteworthy as well, was that there was a higher all-cause mortality rate in Estonia between 2001 and 2003. Although Estonia had a lower IHD mortality rate during this time, the difference in all-cause mortality appeared to be due to higher cerebrovascular and other mortality rates in Estonia than Lithuania.

The AAPC decline in ischemic heart disease mortality rates in Estonia was nearly 3 times the magnitude of Lithuania. As well, in Lithuania, the proportion of all-cause mortality deaths attributed to ischemic heart disease remained relatively stable, whereas in Estonia, the proportion declined substantially over time. The greater progress in ischemic heart disease mortality in Estonia as compared to Lithuania mirror the findings in the 2019 data from the Global Burden of Disease (GBD) Study; that is Estonia has a higher life expectancy of 81.8 years and 74.0 years in women and men respectively, and 78.0 years in both sexes, relative to Lithuania, where it is 80.7 years and 71.5 years in women and men, respectively, and 76.2 years in both sexes [8, 25]. It is worth noting that the trends in all-cause mortality and IHD showed very subtle differences between sexes, with the same general trends across time. The progress in Estonia is also apparent when compared to other surrounding countries (e.g., Russian Federation [31]). While Estonia has seen improvements and a reduction in the life expectancy gap with other EU countries, there remains a larger gap with Lithuania [9, 20, 31, 32].

Overall, we found that Estonia had an increasing proportion of deaths due to other CVD, and a growing proportion of deaths due to cancers, despite a general trend of declining mortality rate due cancers. In Estonia, given that the decline in cancer mortality rate (approximate 30 deaths per 100,000 individuals) was not as steep as all other CVD (600 deaths per 100,000 individuals) this explains the increasing proportion due to cancer deaths. Some of the change in all other CVD mortality rate may reflect a change in coding practices in the reporting of causes of death (differences in coding practices have been also reported elsewhere, [18]), with decreasing emphasis on IHD as a cause of death. Still, when combining any CVD-related cause of death, it is clear that the cardiovascular diseases (i.e., cerebrovascular, ischemic heart disease, all other CVD), exhibited a downward trend in Estonia that was steeper than Lithuania (see Supplementary Materials). A high proportion of CVD mortality and of cancer is in line with countries in North America and Western Europe [8]. In some of these countries, the burden of CVD is continuing to decline, accompanied by a rise in the burden of disease of cancer deaths [11, 33]. Our findings indicate that Estonia may be developing a profile similar to the Western European counterparts, and thus entering an epidemiological transition.

A significant risk factor of ischemic heart disease is heavy episodic drinking, and given the history of high alcohol consumption in Lithuania, it would be reasonable to suspect that alcohol may play a significant role in the burden of disease [12, 34]. Despite a decline in all-cause mortality, there was a relatively high proportion of ischemic heart disease deaths. Heart disease is a product of multiple risk factors, many of which are behaviour-based (e.g., diet, exercise, smoking, alcohol consumption). Improving health outcomes in Lithuania may include reducing alcohol consumption, increasing public awareness of risk factors for ischemic heart disease (e.g., lack of exercise, poor diet) and developing prevention programs for ischemic heart disease. Indeed, Estonia transitioned to insurance-based healthcare and increased the number of family medicine/general practitioners (which improves access to medical care, and improves patient involvement in healthcare) earlier than Lithuania [35–37]. As well, there is less funding spent on emergency care in Estonia, and more spent on healthcare management, preventative measures, and out of hospital care. These factors may have led to better treatment of ischemic heart disease, however the causal impact on mortality is an area for future studies [38, 39].

Identifying the causal factors leading to the differences found between these countries will require additional studies. Notably we identified periods of change using the joinpoint analysis; specifically in 2007 for all-cause mortality in both countries, and 2009 for IHD in both countries. A key event that may have been related to the change in mortality in 2007 would be the financial crisis, as declining discretionary spending would decrease alcohol consumption and alcohol-attributable harm, as found elsewhere [40]. For Estonia there was a steeper decline in IHD, which could be due to a large increase in alcohol taxation just prior to 2009 [41]. As alluded to above, other contributing factors may include healthcare spending, or alcohol control policies as they relate to IHD. Recent alcohol control policy changes have begun to reverse alcohol consumption trends in both countries [32, 41]. Lithuania has decreased its alcohol consumption from a peak of 14.7 L of pure alcohol consumption per capita (15 + years of age) in 2011, to 11.1 L of pure alcohol consumption per capita (15 + years of age) in 2019. Estonia, on the other hand, has more substantially decreased its consumption from an earlier peak of 14.8 L of pure alcohol consumption per capita in 2007, to 10.4 L of pure alcohol consumption per capita in 2019 [42].

There are a few limitations to note in our analyses. First, we separated causes of death into only eight major categories according to the GBD; however, there are various other ways to categorize cause of death data. Second, our findings are observational in nature, thus we can only hypothesize as to why these countries differ. Other studies could employ more focused analyses which can support causal attributions (e.g., interrupted time series analyses) investigating causal factors (e.g., alcohol consumption) on these mortality trends. Finally, we looked at only two countries and their respective profiles to estimate an epidemiological transition, however future studies may also compare the changes across all of the Baltic states (including Latvia), as well as surrounding countries (e.g., Northern European countries).

In the present paper, we have demonstrated that Estonia and Lithuania, two geographically close, high-income Baltic countries in the European Union with similar history pertaining to the former Soviet Union, exhibit different epidemiological profiles with respect to mortality. There were notable differences in temporal trends in all-cause mortality rates, as well as cause-specific mortality rates. Furthermore, examination of the proportional contribution of CVD suggests key differences in ischemic heart disease mortality, which may led to differences in life expectancy these two countries. By identifying cause-specific mortality trends over time, interventions aimed at reducing the main contributors to all-cause mortality rate can be formulated, which can lead to improvements in health and social development.

## Supplementary Information

**Additional file 1.**

## Data Availability

The original data are administrative data of the Lithuanian government agencies, and Estonian agencies, and need to be obtained directly from the original source (exact sources as indicated in the article) or by contacting the corresponding author. The R code used to analyze and compute variables can be found in the Supplementary Materials online or can be obtained from the corresponding author.
