# Supplementary material for: The temporal trend of cause-specific mortality: comparing Estonia and Lithuania, 2001 – 2019
Source: BMC Public Health. 2022 Oct 30;22:1984. doi: 10.1186/s12889-022-14354-8 (PMC9618211; doi:10.1186/s12889-022-14354-8)

## Supplementary Materials

Table S1. Table of ICD-10 codes included in each cause-specific category.

| **Cause-specific Mortality Category** | **ICD-10 Codes** |
| --- | --- |
| Neoplasms | C00-C07, C08-C19.0, C20, C21-C21.8, C22-C22.4, C22.7-C23, C24-C26.1, C26.8-C26.9, C30-C30.1, C31-C33, C34-C34.92, C37-C37.0, C38-C39.9, C40-C41.4, C41.8-C41.9, C43-C45.2, C45.7, C45.9, C47-C49, C50-C50.629, C50.8-C52, C53-C54.3, C54.8-C56.2, C56.9-C58.0, C60-C64.2, C64.9-C69.92, C70-C70.1, C70.9-C73, C74-C75.5, C75.8-C79.9, C80-C81.49, C81.7-C81.79, C81.9-C85.29, C85.7-C86.6, C88-C90.32, C91-C93.7, C93.9-C95.2, C95.7-C97.9, D00-D24.9, D26.0-D39.9, D40-D49.9, E34.0, K51.4-K51.419, K62.0-K62.3, K63.5, N60-N60.99, N84.0-N84.1, N87-N87.9, Z03.1, Z08-Z09.9, Z12-Z12.9, Z80-Z80.9, Z85-Z85.9, Z86.0-Z86.03 |
| Ischemic Heart Disease | I20-I25 |
| Cerebrovascular Disease | I60-I69 |
| All Other CVD | I00-I19, I26-I59, I70-I99, D63.1, E08-E08.9, E10-E14.9, I12-I13.9, N00-N08.8, N15.0, N17-N19, Q60-Q63.2, Q63.8-Q63.9, Q64.2-Q64.9, R73-R73.9, Z13.1, Z49-Z49.32, Z52.4, Z83.3, Z99.2 |
| Digestive Diseases | I84-I85.9, I98.2, K20-K23.8, K25-K31.9, K35-K38.9, K40-K42.9, K44-K46.9, K50-K51.319, K51.5-K52, K52.2-K52.9, K55-K62, K62.4-K62.6, K62.8-K63.4, K63.8-K67, K67.8-K68.1, K68.12-K68.9, K70-K75, K75.2, K75.4-K76.2, K76.4-K77.8, K80-K80.81, K81-K83.9, K85-K87.1, K90-K90.9, K92-K92.9, K93.8, R11-R19.8, R85-R85.9, Z13.81-Z13.818, Z43.1-Z43.4, Z52.6, Z83.7-Z83.79, Z87.1-Z87.19, Z94.4 |
| Unintentional injuries and related conditions | V00-V86.99, V87.2-V87.3, V88.2-V88.3, V90-V98.8, D69.5-D69.59, D70.1-D70.2, D78-D78.89, D89.81-D89.813, E03.2, E06.4, E09-E09.9, E16.0, E23.1, E24.2, E27.3, E36-E36.8, E66.1, E86.02-E87.99, E89-E89.9, G21.0-G21.19, G24.0-G24.09, G25.1, G25.4, G25.6-G25.79, G62.0, G72.0, G93.7, G96.0, G96.11, G97-G97.9, H02.81-H02.819, H05.33-H05.339, H05.42-H05.53, H44.6-H44.799, H59-H59.89, H91.0-H91.09, H95-H95.9, I95.2-I95.81, I97-I97.9, J70-J70.5, J95-J95.9, K08.5-K08.59, K43-K43.9, K52.0, K62.7, K68.11, K91-K91.9, K94-K95.89, L23.3, L27.0-L27.1, L55-L55.9, L56.0-L56.1, L58-L58.9, L64.0, L76-L76.82, M10.2-M10.29, M60.2-M60.28, M87.1-M87.19, M96-M96.9, N14-N14.4, N30.4-N30.41, N46.021, N46.121, N52.2-N52.39, N65-N65.1, N99-N99.9, P93-P93.8, P96.2, P96.5, R50.2-R50.83, W00-W46.2, W49-W62.9, W64-W70.9, W73-W81.9, W83-W94.9, W97.9, W99, X00-X06.9, X08-X44.9, X46-X58.9, Y10-Y14.9, Y16-Y19.9, Y40-Y84.9, Y88-Y88.3, Z21.0, Z42-Z43.0, Z43.8-Z43.9, Z48-Z48.9, Z51-Z51.9, Z88-Z88.9, Z92-Z94.0, Z94.6, Z94.8-Z94.9, Z96-Z96.49, Z96.6-Z97.2, Z97.8-Z99.12, Z99.3-Z99.9, X45, X59, X65, Y09, Y15, Y20-Y34, Y85, Y86 |
| Self-harm and interpersonal violence | T74.2-T76.22, U00-U03, X60-X64.9, X66-X99.9, Y00-Y08.9, Y35-Y38.9, Y87.0-Y87.2, Y89.0-Y89.1 |
| Other causes | All codes not listed above |

Table S2: Proportional (percentage) contributions of 8 cause-specific mortality to all-cause mortality, and yearly percentage changes for Estonia (both sexes)

|  | Other Causes | % Change | Unintentional Injuries and related conditions | % Change | Self-harm and interpersonal violence | % Change | Digestive Diseases | % Change | All Other CVD | % Change | Ischemic Heart Disease | % Change | Cerebrovascular Disease | % Change | Neoplasms | %  Change |
| --- | --- | --- | --- | --- | --- | --- | --- | --- | --- | --- | --- | --- | --- | --- | --- | --- |
| 2001 | 10.7 |  | 7.5 |  | 2.3 |  | 3.3 |  | 7.9 |  | 35.1 |  | 16.8 |  | 16.4 |  |
| 2002 | 11 | 0.3 | 6.3 | -1.2 | 2.1 | -0.2 | 3.3 | 0.1 | 8.7 | 0.7 | 34.8 | -0.4 | 16.7 | -0.1 | 17.1 | 0.7 |
| 2003 | 11.4 | 0.5 | 5.9 | -0.4 | 1.9 | -0.2 | 3.2 | -0.2 | 10 | 1.3 | 34.4 | -0.4 | 16.4 | -0.3 | 16.8 | -0.3 |
| 2004 | 11.2 | -0.3 | 6 | 0.1 | 1.9 | -0.1 | 3.3 | 0.1 | 12.8 | 2.8 | 32 | -2.4 | 14.8 | -1.6 | 18.1 | 1.3 |
| 2005 | 11 | -0.2 | 5.8 | -0.2 | 1.7 | -0.1 | 3.6 | 0.4 | 13.9 | 1.1 | 31.1 | -0.9 | 14.4 | -0.4 | 18.5 | 0.3 |
| 2006 | 11.2 | 0.2 | 6 | 0.2 | 1.5 | -0.2 | 3.5 | -0.2 | 14.4 | 0.4 | 30.7 | -0.4 | 13.8 | -0.6 | 19.1 | 0.6 |
| 2007 | 12 | 0.8 | 6 | 0 | 1.6 | 0.1 | 3.8 | 0.3 | 17.1 | 2.7 | 29.3 | -1.4 | 11.2 | -2.6 | 19 | -0.1 |
| 2008 | 9.1 | -2.9 | 5.2 | -0.8 | 1.6 | -0.1 | 4 | 0.2 | 20.3 | 3.2 | 29.9 | 0.6 | 9.9 | -1.3 | 20.2 | 1.2 |
| 2009 | 8.4 | -0.7 | 4.9 | -0.2 | 1.8 | 0.3 | 3.7 | -0.2 | 22 | 1.8 | 29 | -0.9 | 9 | -0.9 | 21.1 | 0.9 |
| 2010 | 8.1 | -0.3 | 4.6 | -0.3 | 1.5 | -0.3 | 3.7 | -0.1 | 22.4 | 0.3 | 29.1 | 0.1 | 8.9 | -0.1 | 21.8 | 0.7 |
| 2011 | 8.2 | 0.2 | 5 | 0.4 | 1.6 | 0.1 | 3.5 | -0.1 | 23.3 | 0.9 | 26.9 | -2.2 | 8.4 | -0.5 | 23.2 | 1.4 |
| 2012 | 8.1 | -0.1 | 5.2 | 0.3 | 1.7 | 0.1 | 3.4 | -0.1 | 24.6 | 1.3 | 26.7 | -0.1 | 6.9 | -1.4 | 23.3 | 0.1 |
| 2013 | 8.6 | 0.5 | 4.4 | -0.8 | 1.6 | -0.1 | 3.6 | 0.2 | 26.3 | 1.8 | 23.8 | -3 | 7.9 | 1 | 23.8 | 0.5 |
| 2014 | 7.9 | -0.7 | 4.3 | -0.1 | 1.7 | 0.1 | 3.8 | 0.2 | 28.9 | 2.6 | 22.5 | -1.3 | 6.2 | -1.8 | 24.8 | 1 |
| 2015 | 7.4 | -0.5 | 4.1 | -0.1 | 1.5 | -0.2 | 4 | 0.3 | 30.6 | 1.7 | 21.3 | -1.2 | 5.6 | -0.6 | 25.4 | 0.6 |
| 2016 | 7.8 | 0.4 | 4.2 | 0.1 | 1.4 | -0.1 | 4.1 | 0.1 | 30.7 | 0.1 | 20.5 | -0.8 | 6.1 | 0.6 | 25.1 | -0.3 |
| 2017 | 8.1 | 0.3 | 4 | -0.2 | 1.6 | 0.2 | 4.1 | -0.1 | 31.6 | 0.9 | 19.6 | -0.9 | 5.8 | -0.3 | 25.3 | 0.2 |
| 2018 | 8.5 | 0.5 | 3.9 | -0.1 | 1.4 | -0.3 | 4.2 | 0.1 | 31.8 | 0.2 | 18.7 | -0.9 | 5.7 | -0.1 | 25.6 | 0.5 |
| 2019 | 8.9 | 0.4 | 3.7 | -0.1 | 1.4 | 0.1 | 4.3 | 0.1 | 33.1 | 1.4 | 15.4 | -3.3 | 7.2 | 1.5 | 25.7 | 0.1 |

Note: The three periods of interest (beginning, mid-point, final time point) are highlighted in grey.

Table S3: Proportional (percentage) contributions of 8 cause-specific mortality to all-cause mortality, and yearly percentage changes for Lithuania (both sexes)

|  | Other Causes | % Change | Unintentional Injuries and related conditions | % Change | Self-harm and interpersonal violence | % Change | Digestive Diseases | % Change | All Other CVD | % Change | Ischemic Heart Disease | % Change | Cerebrovascular Disease | % Change | Neoplasms | %  Change |
| --- | --- | --- | --- | --- | --- | --- | --- | --- | --- | --- | --- | --- | --- | --- | --- | --- |
| 2001 | 7 |  | 6.4 |  | 3.4 |  | 3.1 |  | 10.4 |  | 40.1 |  | 13.8 |  | 17.8 |  |
| 2002 | 7.4 | 0.4 | 6.2 | -0.2 | 3.1 | -0.2 | 3.3 | 0.1 | 10.6 | 0.2 | 39.3 | -0.7 | 14.3 | 0.6 | 17.5 | -0.3 |
| 2003 | 7.4 | 0 | 6.3 | 0.1 | 3.2 | 0.1 | 3.5 | 0.2 | 10.6 | 0.1 | 39.4 | 0.1 | 14 | -0.3 | 17.6 | 0.1 |
| 2004 | 7.5 | 0.1 | 6.3 | 0 | 3 | -0.2 | 3.5 | 0 | 10.6 | -0.1 | 39 | -0.4 | 13.9 | -0.1 | 17.8 | 0.2 |
| 2005 | 7.8 | 0.3 | 7 | 0.7 | 2.9 | -0.2 | 3.9 | 0.3 | 10.7 | 0.1 | 38.9 | -0.1 | 13.5 | -0.4 | 17.2 | -0.6 |
| 2006 | 8 | 0.2 | 7.2 | 0.2 | 2.3 | -0.6 | 4.7 | 0.8 | 10.3 | -0.4 | 37.7 | -1.2 | 14.1 | 0.6 | 17.2 | 0 |
| 2007 | 8.8 | 0.8 | 7.1 | -0.1 | 2.2 | -0.1 | 5.4 | 0.7 | 11 | 0.7 | 36.8 | -0.9 | 13.2 | -0.9 | 17.4 | 0.2 |
| 2008 | 8.3 | -0.5 | 6.6 | -0.6 | 2.5 | 0.3 | 5.4 | -0.1 | 10.9 | -0.1 | 36.6 | -0.2 | 14.1 | 0.9 | 18.2 | 0.7 |
| 2009 | 8 | -0.3 | 5.7 | -0.9 | 2.6 | 0.1 | 5 | -0.4 | 10.6 | -0.2 | 37 | 0.4 | 14.5 | 0.3 | 18.8 | 0.6 |
| 2010 | 7.8 | -0.2 | 5.9 | 0.2 | 2.4 | -0.2 | 5 | 0 | 10.2 | -0.4 | 38.3 | 1.3 | 14.2 | -0.3 | 18.8 | 0 |
| 2011 | 8 | 0.1 | 5.6 | -0.3 | 2.6 | 0.1 | 4.8 | -0.2 | 10.5 | 0.3 | 37.8 | -0.5 | 14.4 | 0.2 | 19.5 | 0.7 |
| 2012 | 8.1 | 0.1 | 5.8 | 0.2 | 2.3 | -0.2 | 4.9 | 0.1 | 10.4 | -0.1 | 38.1 | 0.3 | 14.2 | -0.2 | 19.3 | -0.1 |
| 2013 | 8.9 | 0.8 | 5.4 | -0.4 | 2.7 | 0.3 | 5.2 | 0.4 | 11.6 | 1.2 | 37.7 | -0.5 | 14.5 | 0.3 | 19 | -0.3 |
| 2014 | 8.8 | -0.1 | 5.4 | 0 | 2.4 | -0.3 | 5.1 | -0.1 | 11.2 | -0.4 | 37.5 | -0.2 | 13.9 | -0.6 | 20.1 | 1.1 |
| 2015 | 9.1 | 0.3 | 5.1 | -0.3 | 2.2 | -0.2 | 5 | -0.2 | 11.3 | 0.1 | 38 | 0.5 | 13.8 | -0.1 | 22 | 0.4 |
| 2016 | 9.3 | 0.3 | 5.1 | 0 | 2.2 | -0.1 | 5.2 | 0.3 | 11.5 | 0.2 | 37.4 | -0.6 | 13.7 | -0.1 | 22 | 0 |
| 2017 | 10 | 0.6 | 4.9 | -0.2 | 2 | -0.2 | 4.9 | -0.3 | 11.9 | 0.5 | 36.2 | -1.1 | 13.4 | -0.3 | 22.3 | 0.3 |
| 2018 | 10.6 | 0.7 | 4.7 | -0.2 | 1.8 | -0.1 | 4.8 | -0.1 | 12.5 | 0.6 | 35 | -1.3 | 13.5 | 0.1 | 22.8 | 0.5 |
| 2019 | 10.5 | -0.1 | 4.4 | -0.3 | 1.8 | 0 | 5 | 0.2 | 12.4 | -0.1 | 34.3 | -0.7 | 12.8 | -0.7 | 23.7 | 0.9 |

Note: The three periods of interest (beginning, mid-point, final time point) are highlighted in grey.

Table S4: Annual percentage change in mortality rate from joinpoint analysis for 7 other disease categories (both sexes)

|  | Time period | APC | 95% CI | p-value |
| --- | --- | --- | --- | --- |
| Neoplasms | | | | |
| Estonia |  |  |  |  |
| Segment 1 | 2001 – 2005 | 0.75 | [-0.43, 1.95] | p = 0.20 |
| Segment 2 | 2005 – 2019 | -0.24 | [-0.38, -0.09] | p < 0.001 |
| Lithuania |  |  |  |  |
| Segment 1 | 2001 – 2019 | -0.38 | [-0.52, -0.25] | p < .001 |
| Gastrointestinal Disease | | | | |
| Estonia |  |  |  |  |
| Segment 1 | 2001 – 2008 | 0.26 | [-0.87, 1.41] | p = 0.61 |
| Segment 2 | 2008 – 2012 | -7.47 | [-11.82, -2.90] | p = 0.01 |
| Segment 3 | 2012 – 2015 | 4.08 | [-7.01, 16.49] | p = 0.44 |
| Segment 4 | 2015 – 2019 | -0.04 | [-2.90, 2.91] | p = 0.98 |
| Lithuania |  |  |  |  |
| Segment 1 | 2001 – 2004 | 3.12 | [-3.83, 10.57] | p = 0.34 |
| Segment 2 | 2004 – 2007 | 16.60 | [1.35, 34.14] | p = 0.04 |
| Segment 3 | 2007 – 2010 | -6.70 | [-18.90, 7.33] | p = 0.29 |
| Segment 4 | 2010 – 2019 | -1.40 | [-2.64, -0.15] | p = 0.03 |
| Self-harm and Interpersonal Violence | | | | |
| Estonia |  |  |  |  |
| Segment 1 | 2001 – 2006 | -8.47 | [-11.36, -5.49] | p < .001 |
| Segment 2 | 2006 – 2019 | -3.70 | [-4.35, -3.05] | p < .001 |
| Lithuania |  |  |  |  |
| Segment 1 | 2001 – 2007 | -5.82 | [-7.93, -3.66] | p < 0.001 |
| Segment 2 | 2007 – 2014 | -1.98 | [-4.16, 0.24] | p = 0.08 |
| Segment 3 | 2014 – 2019 | -7.87 | [-10.67, -4.99] | p < 0.001 |
| Other mortality rate | | | | |
| Estonia |  |  |  |  |
| Segment 1 | 2001 – 2007 | -1.65 | [-4.22, 0.99] | p = 0.19 |
| Segment 2 | 2007 – 2010 | -16.35 | [-28.37, -2.30] | p = 0.03 |
| Segment 3 | 2010 – 2019 | -1.20 | [-2.60, 0.22] | p = 0.09 |
| Lithuania |  |  |  |  |
| Segment 1 | 2001 – 2004 | 0.93 | [-2.42, 4.40] | p = 0.54 |
| Segment 2 | 2004 – 2007 | 5.96 | [-2.58, 15.24] | p = 0.15 |
| Segment 3 | 2007 – 2010 | -7.66 | [-15.10, 0.44] | p = 0.06 |
| Segment 4 | 2010 – 2019 | 2.16 | [1.69, 2.63] | p = 0.00 |
| Other Cardiovascular Diseases | | | | |
| Estonia |  |  |  |  |
| Segment 1 | 2001 – 2008 | 11.08 | [9.08, 13.13] | p < .001 |
| Segment 2 | 2008 – 2019 | 2.14 | [1.19, 3.10] | p < .001 |
| Lithuania |  |  |  |  |
| Segment 1 | 2001 – 2007 | 1.06 | [0.20, 1.93] | p = 0.02 |
| Segment 2 | 2007 – 2010 | -5.31 | [-12.57, 2.55] | p = 0.16 |
| Segment 3 | 2010 – 2019 | 0.61 | [0.17, 1.04] | p = 0.01 |
| Cerebrovascular Disease | | | | |
| Estonia |  |  |  |  |
| Segment 1 | 2001 – 2003 | -1.89 | [-27.75, 33.21] | p = 0.89 |
| Segment 2 | 2003 – 2015 | -11.43 | [-12.67, -10.18] | p < 0.001 |
| Segment 3 | 2015 – 2019 | 2.02 | [-5.35, 9.96] | p = 0.57 |
| Lithuania |  |  |  |  |
| Segment 1 | 2001 – 2007 | -0.09 | [-0.96, 0.80] | p = 0.83 |
| Segment 2 | 2007 – 2016 | -2.09 | [-2.61, -1.56] | p < 0.001 |
| Segment 3 | 2016 – 2019 | -4.32 | [-7.35, -1.20] | p = 0.01 |
| Unintentional Injuries and related conditions | | | | |
| Estonia |  |  |  |  |
| Segment 1 | 2001 – 2019 | -6.04 | [-6.51, -5.56] | p < .001 |
| Lithuania |  |  |  |  |
| Segment 1 | 2001 – 2003 | -3.98 | [-15.63, 9.29] | p = 0.49 |
| Segment 2 | 2003 – 2006 | 8.69 | [-3.99, 23.05] | p = 0.16 |
| Segment 3 | 2006 – 2009 | -9.99 | [-20.49, 1.90] | p = 0.09 |
| Segment 4 | 2009 – 2019 | -4.24 | [-4.88, -3.59] | p < 0.001 |

Table S5: Proportional (percentage) contributions of 8 cause-specific mortality to all-cause mortality, and yearly percentage changes for Lithuania (males)

|  | Other Causes | % Change | Unintentional Injuries and related conditions | % Change | Self-harm and interpersonal violence | % Change | Digestive Diseases | % Change | All Other CVD | % Change | Ischemic Heart Disease | % Change | Cerebrovascular Disease | % Change | Neoplasms | %  Change |
| --- | --- | --- | --- | --- | --- | --- | --- | --- | --- | --- | --- | --- | --- | --- | --- | --- |
| 2001 | 9.1 |  | 7.8 |  | 8.1 |  | 4.4 |  | 3.1 |  | 36.7 |  | 10.9 |  | 19.8 |  |
| 2002 | 9.2 | 0.1 | 8.2 | 0.4 | 7.5 | -0.6 | 4.2 | -0.2 | 3.2 | 0.1 | 37.1 | 0.4 | 11.1 | 0.2 | 19.5 | -0.3 |
| 2003 | 9.4 | 0.2 | 7.8 | -0.4 | 7.7 | 0.2 | 4.1 | -0.1 | 3.5 | 0.3 | 37.3 | 0.2 | 11.1 | 0 | 19.1 | -0.4 |
| 2004 | 9.6 | 0.2 | 8.1 | 0.3 | 7.9 | 0.2 | 3.9 | -0.2 | 3.5 | 0 | 36.2 | -1.1 | 11 | -0.1 | 19.9 | 0.8 |
| 2005 | 9.9 | 0.3 | 8.5 | 0.4 | 8.6 | 0.7 | 3.7 | -0.2 | 3.8 | 0.3 | 36.4 | 0.2 | 10.4 | -0.6 | 18.7 | -1.2 |
| 2006 | 9.9 | 0 | 8.3 | -0.2 | 8.9 | 0.3 | 3 | -0.7 | 4.8 | 1 | 35 | -1.4 | 11 | 0.6 | 19.2 | 0.5 |
| 2007 | 10.9 | 1 | 8.9 | 0.6 | 8.6 | -0.3 | 2.8 | -0.2 | 5.3 | 0.5 | 33.6 | -1.4 | 10.4 | -0.6 | 19.5 | 0.3 |
| 2008 | 10.2 | -0.7 | 8.1 | -0.8 | 8 | -0.6 | 3.2 | 0.4 | 5.3 | 0 | 34.3 | 0.7 | 11 | 0.6 | 19.9 | 0.4 |
| 2009 | 9.9 | -0.3 | 8.1 | 0 | 7 | -1 | 3.4 | 0.2 | 4.8 | -0.5 | 34.7 | 0.4 | 11.3 | 0.3 | 20.9 | 1 |
| 2010 | 9.3 | -0.6 | 7.4 | -0.7 | 7.3 | 0.3 | 3 | -0.4 | 5 | 0.2 | 35.8 | 1.1 | 11.1 | -0.2 | 21 | 0.1 |
| 2011 | 9.6 | 0.3 | 7.2 | -0.2 | 6.8 | -0.5 | 3.2 | 0.2 | 4.6 | -0.4 | 36 | 0.2 | 11.4 | 0.3 | 21.3 | 0.3 |
| 2012 | 9.6 | 0 | 7.1 | -0.1 | 7 | 0.2 | 3 | -0.2 | 4.8 | 0.2 | 35.8 | -0.2 | 11.1 | -0.3 | 21.6 | 0.3 |
| 2013 | 10.3 | 0.7 | 6.8 | -0.3 | 6.6 | -0.4 | 3.4 | 0.4 | 5.1 | 0.3 | 35 | -0.8 | 12 | 0.9 | 20.8 | -0.8 |
| 2014 | 10.1 | -0.2 | 6.7 | -0.1 | 6.6 | 0 | 3.1 | -0.3 | 5 | -0.1 | 35 | 0 | 11 | -1 | 22.5 | 1.7 |
| 2015 | 10.7 | 0.6 | 6.9 | 0.2 | 6.2 | -0.4 | 2.9 | -0.2 | 4.8 | -0.2 | 35.3 | 0.3 | 10.6 | -0.4 | 22.6 | 0.1 |
| 2016 | 10.3 | -0.4 | 6.7 | -0.2 | 6.1 | -0.1 | 2.9 | 0 | 5.4 | 0.6 | 35.3 | 0 | 11 | 0.4 | 22.2 | -0.4 |
| 2017 | 10.9 | 0.6 | 8.1 | 1.4 | 5.9 | -0.2 | 2.6 | -0.3 | 4.8 | -0.6 | 34 | -1.3 | 11.1 | 0.1 | 22.7 | 0.5 |
| 2018 | 11.9 | 1 | 8.3 | 0.2 | 5.5 | -0.4 | 2.4 | -0.2 | 4.7 | -0.1 | 32.9 | -1.1 | 11.2 | 0.1 | 23 | 0.3 |
| 2019 | 11.3 | -0.6 | 9.1 | 0.8 | 5.2 | -0.3 | 2.5 | 0.1 | 4.9 | 0.2 | 32.6 | -0.3 | 10.6 | -0.6 | 23.9 | 0.9 |

Note: The three periods of interest (beginning, mid-point, final time point) are highlighted in grey.

Table S6: Proportional (percentage) contributions of 8 cause-specific mortality to all-cause mortality, and yearly percentage changes for Lithuania (females)

|  | Other Causes | % Change | Unintentional Injuries and related conditions | % Change | Self-harm and interpersonal violence | % Change | Digestive Diseases | % Change | All Other CVD | % Change | Ischemic Heart Disease | % Change | Cerebrovascular Disease | % Change | Neoplasms | %  Change |
| --- | --- | --- | --- | --- | --- | --- | --- | --- | --- | --- | --- | --- | --- | --- | --- | --- |
| 2001 | 5.2 |  | 9.1 |  | 3.5 |  | 1.6 |  | 3 |  | 44.2 |  | 16.8 |  | 16.6 |  |
| 2002 | 5.8 | 0.6 | 9.3 | 0.2 | 3.8 | 0.3 | 1.3 | -0.3 | 3.2 | 0.2 | 42.6 | -1.6 | 17.8 | 1 | 16.4 | -0.2 |
| 2003 | 5.6 | -0.2 | 9.4 | 0.1 | 3.6 | -0.2 | 1.5 | 0.2 | 3.3 | 0.1 | 42.6 | 0 | 17.2 | -0.6 | 16.9 | 0.5 |
| 2004 | 5.6 | 0 | 9.6 | 0.2 | 3.5 | -0.1 | 1.5 | 0 | 3.4 | 0.1 | 42.8 | 0.2 | 17.1 | -0.1 | 16.6 | -0.3 |
| 2005 | 5.8 | 0.2 | 9.2 | -0.4 | 3.9 | 0.4 | 1.4 | -0.1 | 3.7 | 0.3 | 42.5 | -0.3 | 16.9 | -0.2 | 16.6 | 0 |
| 2006 | 5.9 | 0.1 | 9.2 | 0 | 4.2 | 0.3 | 1.1 | -0.3 | 4.4 | 0.7 | 41.5 | -1 | 17.7 | 0.8 | 16 | -0.6 |
| 2007 | 6.6 | 0.7 | 9.1 | -0.1 | 4.1 | -0.1 | 1 | -0.1 | 5.1 | 0.7 | 41.3 | -0.2 | 16.6 | -1.1 | 16.2 | 0.2 |
| 2008 | 6.4 | -0.2 | 8.3 | -0.8 | 3.8 | -0.3 | 1.2 | 0.2 | 5.1 | 0 | 39.9 | -1.4 | 17.7 | 1.1 | 17.6 | 1.4 |
| 2009 | 6.3 | -0.1 | 8.7 | 0.4 | 3.1 | -0.7 | 1.1 | -0.1 | 4.7 | -0.4 | 40.4 | 0.5 | 18 | 0.3 | 17.6 | 0 |
| 2010 | 6.4 | 0.1 | 7.5 | -1.2 | 3.3 | 0.2 | 1.2 | 0.1 | 4.6 | -0.1 | 42.1 | 1.7 | 17.6 | -0.4 | 17.4 | -0.2 |
| 2011 | 6.3 | -0.1 | 7.6 | 0.1 | 3.3 | 0 | 1.3 | 0.1 | 4.6 | 0 | 40.8 | -1.3 | 17.7 | 0.1 | 18.6 | 1.2 |
| 2012 | 6.6 | 0.3 | 7.3 | -0.3 | 3.4 | 0.1 | 1.1 | -0.2 | 4.6 | 0 | 41.4 | 0.6 | 17.5 | -0.2 | 18.1 | -0.5 |
| 2013 | 7.4 | 0.8 | 6.4 | -0.9 | 3.1 | -0.3 | 1.2 | 0.1 | 5.1 | 0.5 | 41.3 | -0.1 | 17.4 | -0.1 | 18 | -0.1 |
| 2014 | 7.4 | 0 | 6.8 | 0.4 | 3.1 | 0 | 1 | -0.2 | 5 | -0.1 | 41 | -0.3 | 17.2 | -0.2 | 18.6 | 0.6 |
| 2015 | 7.4 | 0 | 6.4 | -0.4 | 3.1 | 0 | 1 | 0 | 4.9 | -0.1 | 41.5 | 0.5 | 17 | -0.2 | 18.7 | 0.1 |
| 2016 | 8.2 | 0.8 | 6.9 | 0.5 | 3.1 | 0 | 0.8 | -0.2 | 4.8 | -0.1 | 40.4 | -1.1 | 16.7 | -0.3 | 19.1 | 0.4 |
| 2017 | 9.1 | 0.9 | 8.2 | 1.3 | 3.2 | 0.1 | 0.9 | 0.1 | 4.8 | 0 | 39 | -1.4 | 15.8 | -0.9 | 19 | -0.1 |
| 2018 | 9.5 | 0.4 | 9 | 0.8 | 3.2 | 0 | 0.8 | -0.1 | 4.5 | -0.3 | 37.7 | -1.3 | 15.9 | 0.1 | 19.3 | 0.3 |
| 2019 | 9.8 | 0.3 | 9.6 | 0.6 | 2.8 | -0.4 | 0.8 | 0 | 4.8 | 0.3 | 36.7 | -1 | 15.2 | -0.7 | 20.3 | 1 |

Note: The three periods of interest (beginning, mid-point, final time point) are highlighted in grey.

Table S7: Proportional (percentage) contributions of 8 cause-specific mortality to all-cause mortality, and yearly percentage changes for Estonia (males)

|  | Other Causes | % Change | Unintentional Injuries and related conditions | % Change | Self-harm and interpersonal violence | % Change | Digestive Diseases | % Change | All Other CVD | % Change | Ischemic Heart Disease | % Change | Cerebrovascular Disease | % Change | Neoplasms | %  Change |
| --- | --- | --- | --- | --- | --- | --- | --- | --- | --- | --- | --- | --- | --- | --- | --- | --- |
| 2001 | 11.1 |  | 7.3 |  | 10.1 |  | 2.8 |  | 3.3 |  | 34.7 |  | 12.8 |  | 17.9 |  |
| 2002 | 11.9 | 0.8 | 7.9 | 0.6 | 8.4 | -1.7 | 2.7 | -0.1 | 3.4 | 0.1 | 33.6 | -1.1 | 12.8 | 0 | 19.3 | 1.4 |
| 2003 | 12.3 | 0.4 | 8.9 | 1 | 7.7 | -0.7 | 2.4 | -0.3 | 3.1 | -0.3 | 32.9 | -0.7 | 13.8 | 1 | 18.9 | -0.4 |
| 2004 | 12.1 | -0.2 | 11.1 | 2.2 | 7.8 | 0.1 | 2.4 | 0 | 3.3 | 0.2 | 31.3 | -1.6 | 11.5 | -2.3 | 20.5 | 1.6 |
| 2005 | 11.7 | -0.4 | 12.7 | 1.6 | 7.2 | -0.6 | 2.1 | -0.3 | 3.6 | 0.3 | 30.4 | -0.9 | 11.3 | -0.2 | 21 | 0.5 |
| 2006 | 11.9 | 0.2 | 13.1 | 0.4 | 7.4 | 0.2 | 1.8 | -0.3 | 3.5 | -0.1 | 30.1 | -0.3 | 11.2 | -0.1 | 20.9 | -0.1 |
| 2007 | 12.5 | 0.6 | 15.6 | 2.5 | 7.5 | 0.1 | 2.2 | 0.4 | 3.8 | 0.3 | 28.3 | -1.8 | 9.2 | -2 | 20.9 | 0 |
| 2008 | 10.1 | -2.4 | 17.9 | 2.3 | 6.5 | -1 | 2 | -0.2 | 4.1 | 0.3 | 29.2 | 0.9 | 8.2 | -1 | 22.1 | 1.2 |
| 2009 | 8.7 | -1.4 | 19.5 | 1.6 | 6.2 | -0.3 | 2.3 | 0.3 | 4.1 | 0 | 28 | -1.2 | 7.6 | -0.6 | 23.5 | 1.4 |
| 2010 | 8.6 | -0.1 | 19.9 | 0.4 | 5.6 | -0.6 | 1.9 | -0.4 | 3.8 | -0.3 | 28.4 | 0.4 | 7.2 | -0.4 | 24.6 | 1.1 |
| 2011 | 9.9 | 1.3 | 19.9 | 0 | 6.1 | 0.5 | 2.1 | 0.2 | 3.4 | -0.4 | 25.9 | -2.5 | 7.4 | 0.2 | 25.4 | 0.8 |
| 2012 | 9.6 | -0.3 | 20.7 | 0.8 | 6.3 | 0.2 | 2.2 | 0.1 | 3.1 | -0.3 | 26.4 | 0.5 | 6 | -1.4 | 25.7 | 0.3 |
| 2013 | 10.8 | 1.2 | 21.9 | 1.2 | 5.5 | -0.8 | 2.1 | -0.1 | 3.6 | 0.5 | 23 | -3.4 | 6.4 | 0.4 | 26.7 | 1 |
| 2014 | 9.9 | -0.9 | 23.8 | 1.9 | 5.2 | -0.3 | 2.2 | 0.1 | 3.9 | 0.3 | 22.4 | -0.6 | 5.4 | -1 | 27.3 | 0.6 |
| 2015 | 9.3 | -0.6 | 25.2 | 1.4 | 5.1 | -0.1 | 2 | -0.2 | 3.9 | 0 | 21.2 | -1.2 | 5 | -0.4 | 28.4 | 1.1 |
| 2016 | 9.1 | -0.2 | 25.7 | 0.5 | 5 | -0.1 | 1.8 | -0.2 | 4.3 | 0.4 | 20.9 | -0.3 | 5.6 | 0.6 | 27.6 | -0.8 |
| 2017 | 9.7 | 0.6 | 26.1 | 0.4 | 4.9 | -0.1 | 2 | 0.2 | 4.1 | -0.2 | 19.8 | -1.1 | 5.4 | -0.2 | 28 | 0.4 |
| 2018 | 10.2 | 0.5 | 26.2 | 0.1 | 4.7 | -0.2 | 1.8 | -0.2 | 4.1 | 0 | 19.1 | -0.7 | 5.1 | -0.3 | 28.7 | 0.7 |
| 2019 | 10.8 | 0.6 | 26.8 | 0.6 | 4.4 | -0.3 | 1.8 | 0 | 4.5 | 0.4 | 16.1 | -3 | 6.9 | 1.8 | 28.8 | 0.1 |

Note: The three periods of interest (beginning, mid-point, final time point) are highlighted in grey.

Table S8: Proportional (percentage) contributions of 8 cause-specific mortality to all-cause mortality, and yearly percentage changes for Estonia (females)

|  | Other Causes | % Change | Unintentional Injuries and related conditions | % Change | Self-harm and interpersonal violence | % Change | Digestive Diseases | % Change | All Other CVD | % Change | Ischemic Heart Disease | % Change | Cerebrovascular Disease | % Change | Neoplasms | %  Change |
| --- | --- | --- | --- | --- | --- | --- | --- | --- | --- | --- | --- | --- | --- | --- | --- | --- |
| 2001 | 10.1 |  | 8.4 |  | 4.1 |  | 1.2 |  | 3.3 |  | 36.7 |  | 20.5 |  | 15.7 |  |
| 2002 | 10.1 | 0 | 9.1 | 0.7 | 3.3 | -0.8 | 1.1 | -0.1 | 3.1 | -0.2 | 36.9 | 0.2 | 20.4 | -0.1 | 16.1 | 0.4 |
| 2003 | 10.7 | 0.6 | 11 | 1.9 | 3 | -0.3 | 1 | -0.1 | 3.1 | 0 | 36.5 | -0.4 | 19.1 | -1.3 | 15.6 | -0.5 |
| 2004 | 10.3 | -0.4 | 14.2 | 3.2 | 3 | 0 | 0.8 | -0.2 | 3 | -0.1 | 33.6 | -2.9 | 17.8 | -1.3 | 17.2 | 1.6 |
| 2005 | 10.7 | 0.4 | 15 | 0.8 | 3.1 | 0.1 | 0.9 | 0.1 | 3.4 | 0.4 | 32.7 | -0.9 | 17.2 | -0.6 | 17.1 | -0.1 |
| 2006 | 10.4 | -0.3 | 15.2 | 0.2 | 3.3 | 0.2 | 0.8 | -0.1 | 3.3 | -0.1 | 32.3 | -0.4 | 16.3 | -0.9 | 18.3 | 1.2 |
| 2007 | 11.5 | 1.1 | 18.4 | 3.2 | 3.1 | -0.2 | 0.7 | -0.1 | 3.6 | 0.3 | 31.2 | -1.1 | 13.4 | -2.9 | 18.1 | -0.2 |
| 2008 | 8.1 | -3.4 | 22.3 | 3.9 | 2.7 | -0.4 | 0.8 | 0.1 | 3.6 | 0 | 31.7 | 0.5 | 11.5 | -1.9 | 19.3 | 1.2 |
| 2009 | 7.7 | -0.4 | 24.1 | 1.8 | 2.6 | -0.1 | 0.9 | 0.1 | 3.2 | -0.4 | 30.9 | -0.8 | 10.5 | -1 | 20.2 | 0.9 |
| 2010 | 7.5 | -0.2 | 24.6 | 0.5 | 2.5 | -0.1 | 0.7 | -0.2 | 3.4 | 0.2 | 30.4 | -0.5 | 10.5 | 0 | 20.3 | 0.1 |
| 2011 | 6.8 | -0.7 | 26.6 | 2 | 2.6 | 0.1 | 0.7 | 0 | 3.3 | -0.1 | 28.1 | -2.3 | 9.5 | -1 | 22.4 | 2.1 |
| 2012 | 6.6 | -0.2 | 28.2 | 1.6 | 2.7 | 0.1 | 0.8 | 0.1 | 3.5 | 0.2 | 27.7 | -0.4 | 8 | -1.5 | 22.5 | 0.1 |
| 2013 | 6.8 | 0.2 | 30.3 | 2.1 | 2.2 | -0.5 | 0.9 | 0.1 | 3.2 | -0.3 | 24.8 | -2.9 | 9.3 | 1.3 | 22.5 | 0 |
| 2014 | 5.9 | -0.9 | 33.7 | 3.4 | 2.3 | 0.1 | 0.8 | -0.1 | 3.2 | 0 | 23 | -1.8 | 7.2 | -2.1 | 23.8 | 1.3 |
| 2015 | 5.7 | -0.2 | 35.6 | 1.9 | 2.3 | 0 | 0.7 | -0.1 | 3.8 | 0.6 | 21.8 | -1.2 | 6.2 | -1 | 24 | 0.2 |
| 2016 | 6.4 | 0.7 | 35.5 | -0.1 | 2.5 | 0.2 | 0.7 | 0 | 3.4 | -0.4 | 20.4 | -1.4 | 6.8 | 0.6 | 24.3 | 0.3 |
| 2017 | 6.4 | 0 | 36.8 | 1.3 | 2.1 | -0.4 | 0.9 | 0.2 | 3.5 | 0.1 | 19.8 | -0.6 | 6.2 | -0.6 | 24.3 | 0 |
| 2018 | 6.9 | 0.5 | 37.1 | 0.3 | 2.3 | 0.2 | 0.6 | -0.3 | 3.9 | 0.4 | 18.4 | -1.4 | 6.3 | 0.1 | 24.5 | 0.2 |
| 2019 | 7 | 0.1 | 38.9 | 1.8 | 2.4 | 0.1 | 0.7 | 0.1 | 3.7 | -0.2 | 14.9 | -3.5 | 7.6 | 1.3 | 24.8 | 0.3 |

Note: The three periods of interest (beginning, mid-point, final time point) are highlighted in grey.


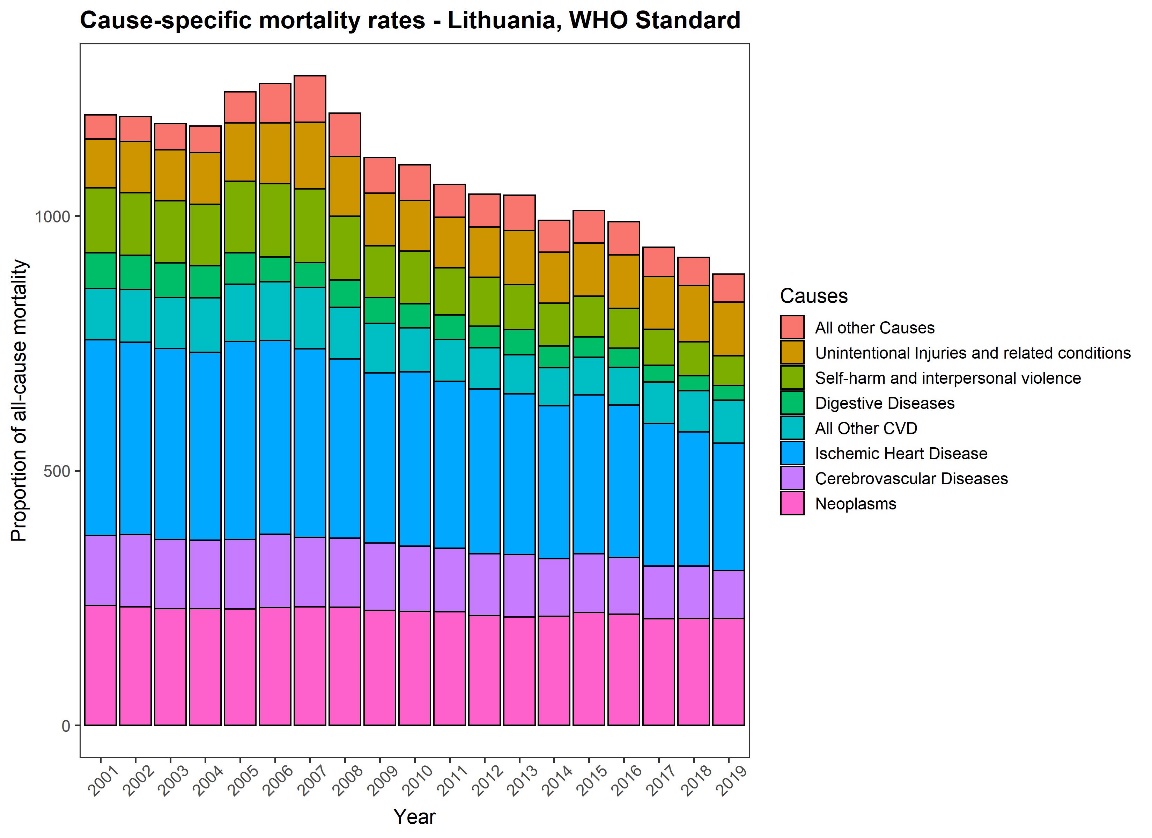


**B**

**A**


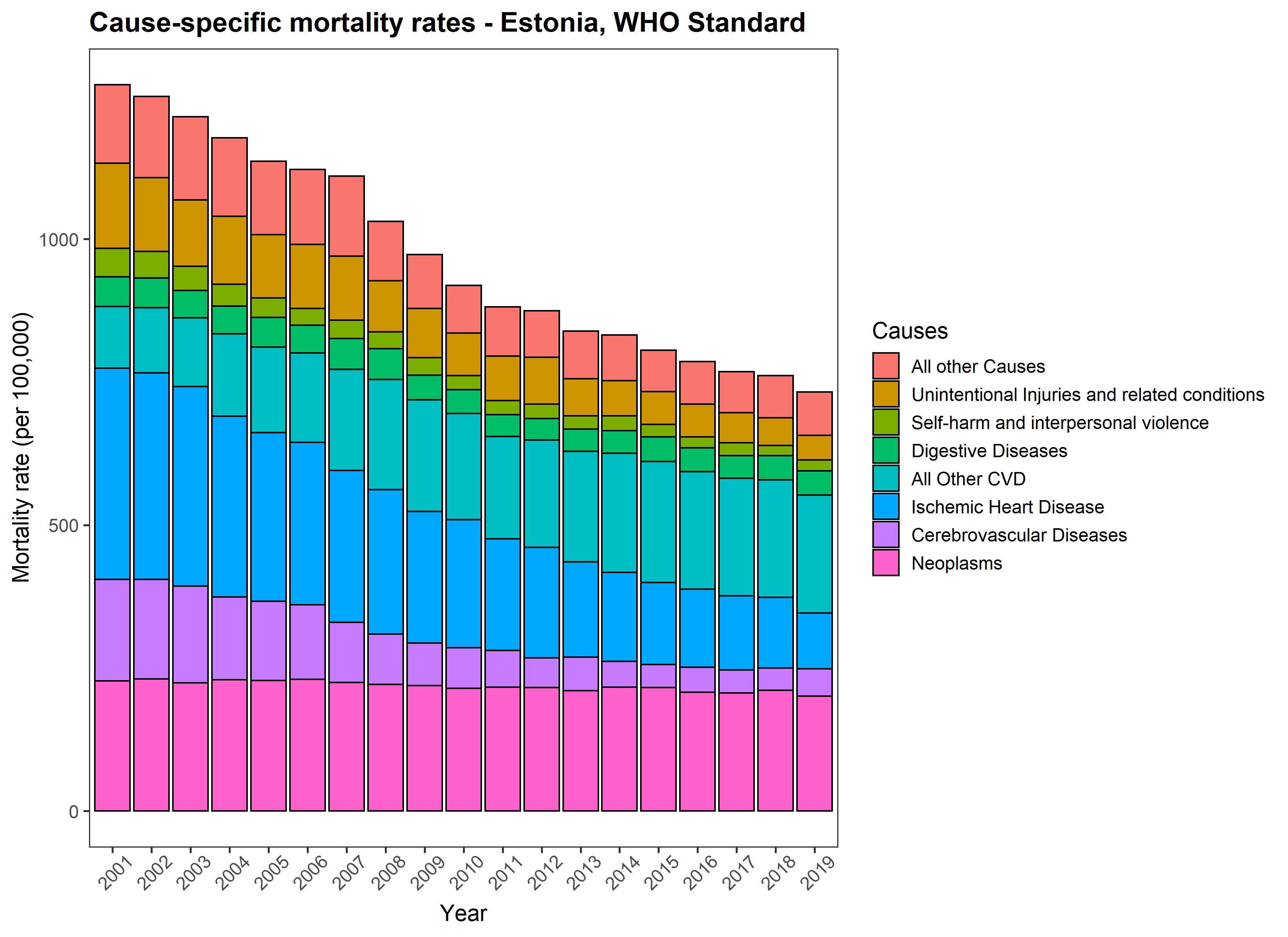


Figure S1. Mortality rate (deaths per 100,000 individuals, 20+ years of age) in Estonia (Graph A) and Lithuania (Graph B), separated by causes between 2001 and 2019 according to WHO standard. Mortality rate in each year across all category sums to all-cause mortality.


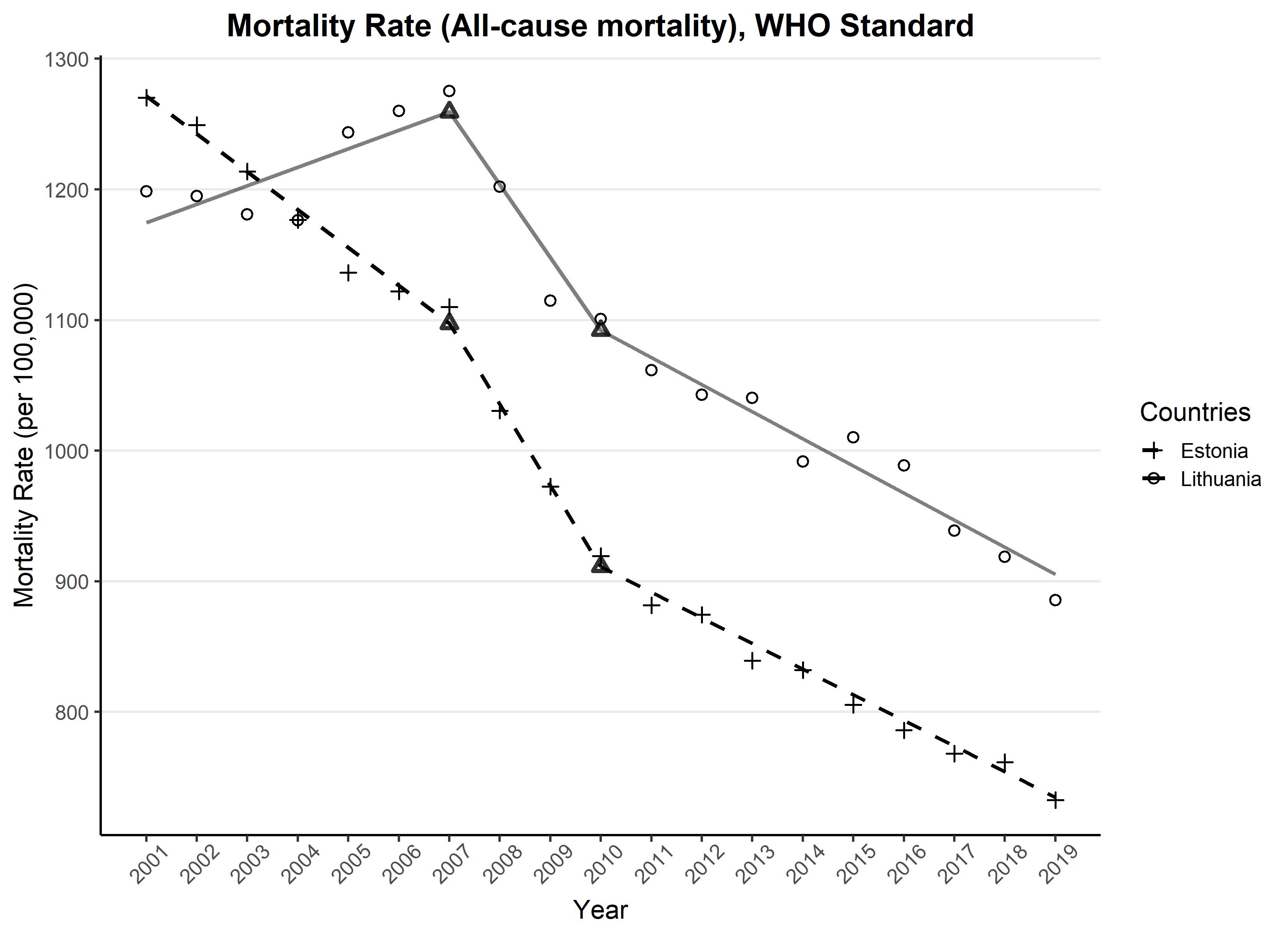


Figure S2. All-cause mortality rate (deaths per 100,000 individuals, 20+ years of age) in Estonia and Lithuania according to WHO standard.


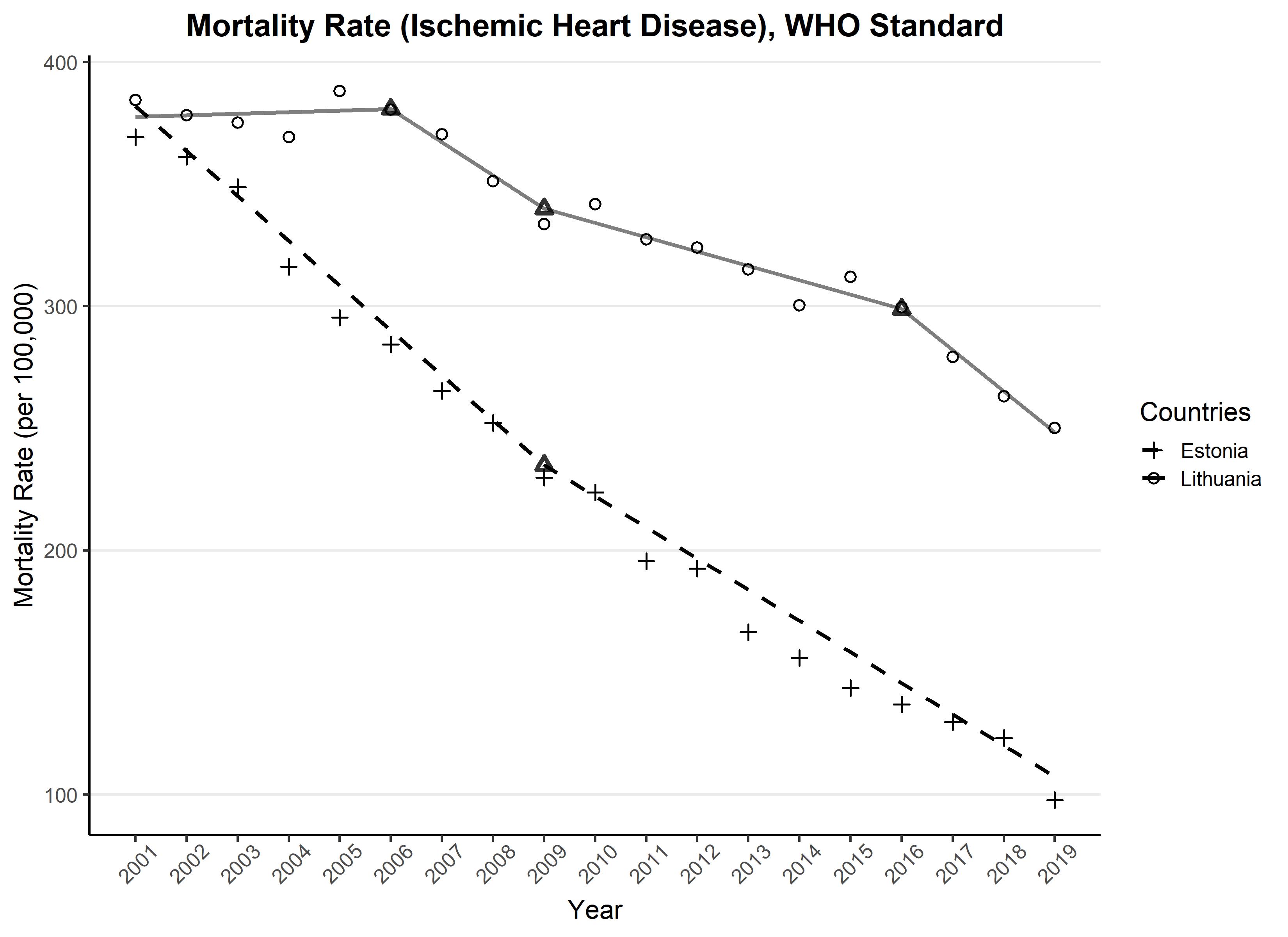


Figure S3. Ischemic heart disease mortality rate (deaths per 100,000 individuals, 20+ years of age) in Estonia and Lithuania according to WHO standard.


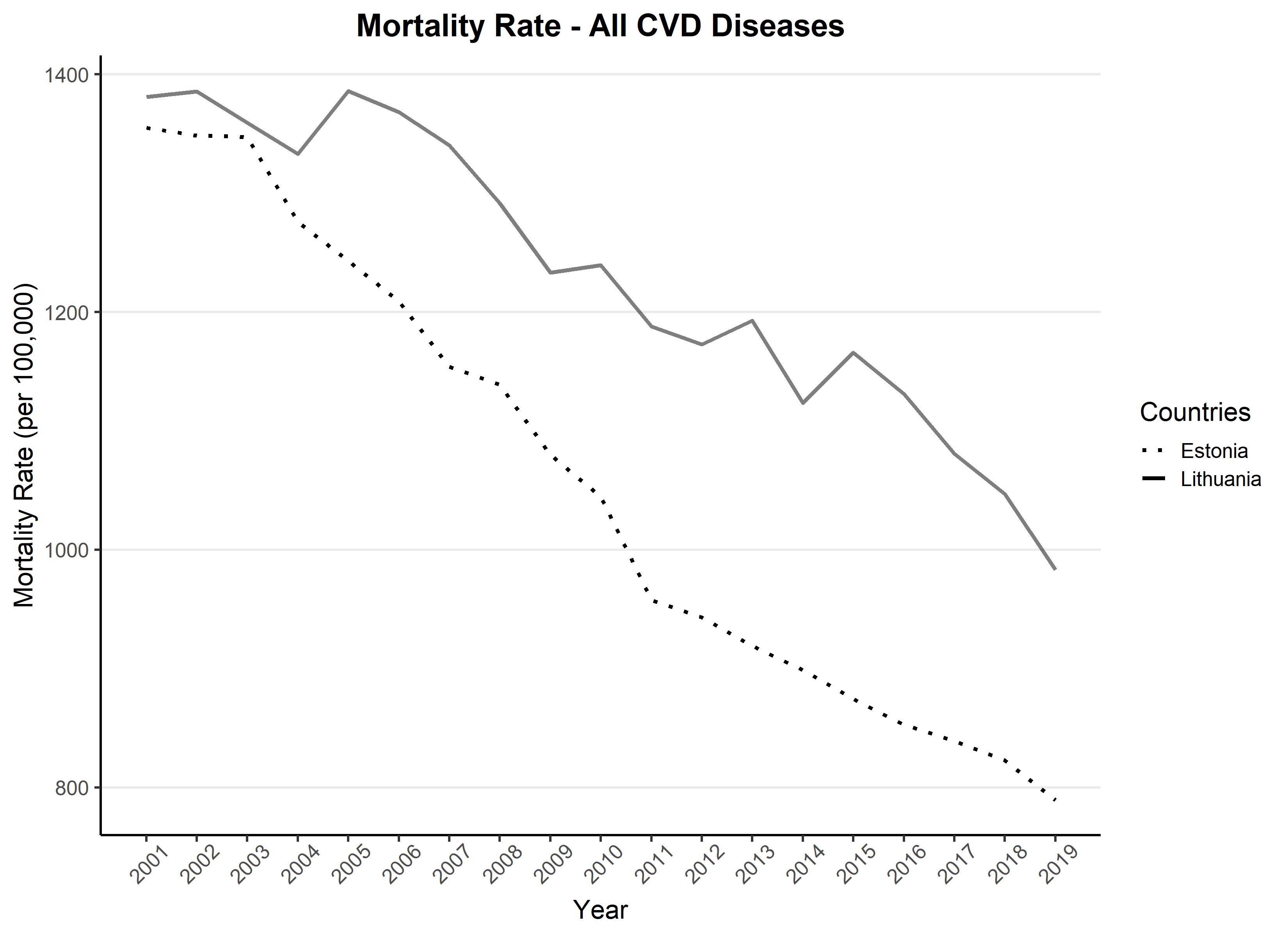


Figure S4. All cardiovascular disease mortality rate (cerebrovascular, ischemic heart disease, all other CVD) as deaths per 100,000 individuals, 20+ years of age, in Estonia and Lithuania according to EU standard.

**A**


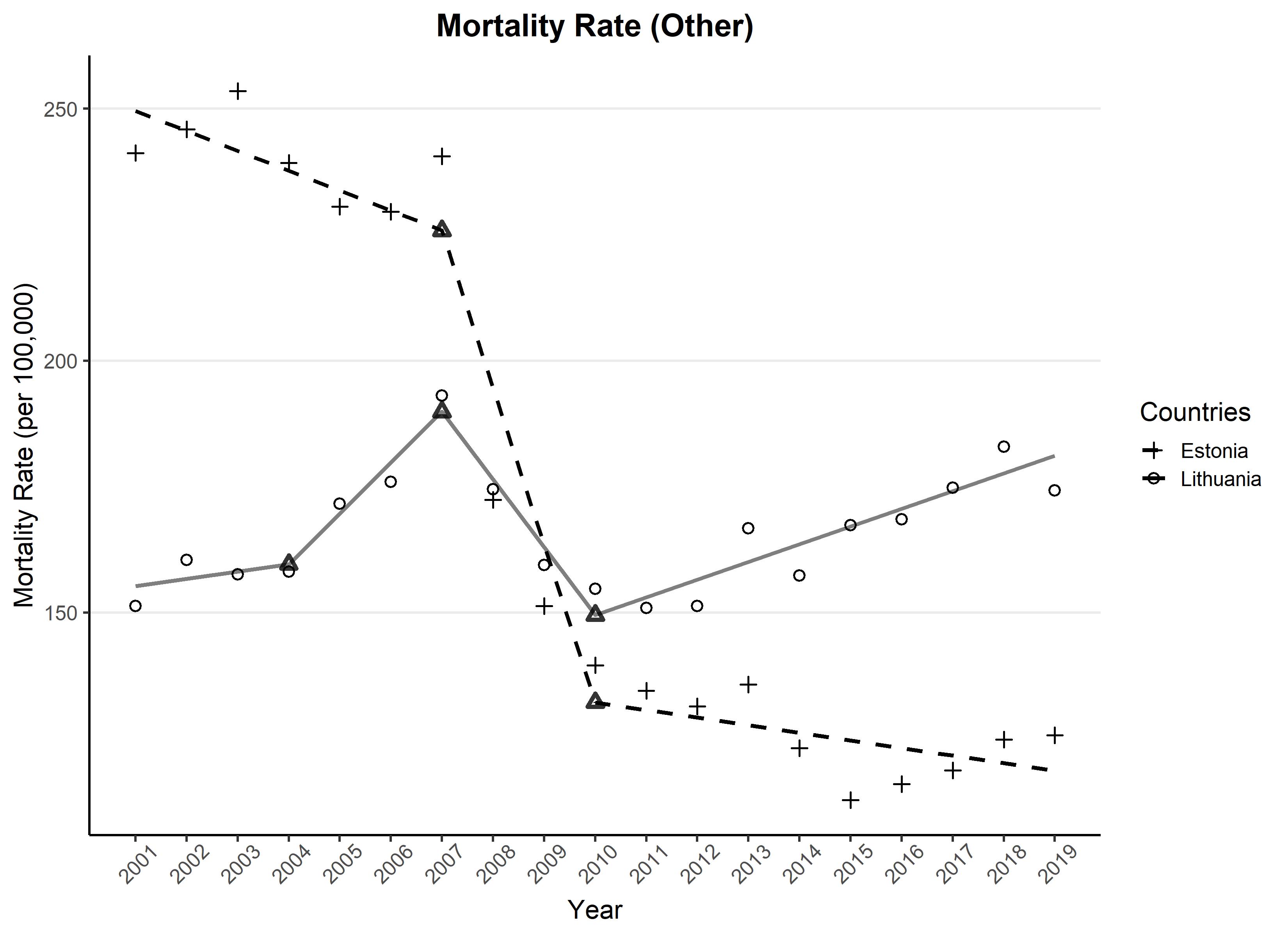


**B**


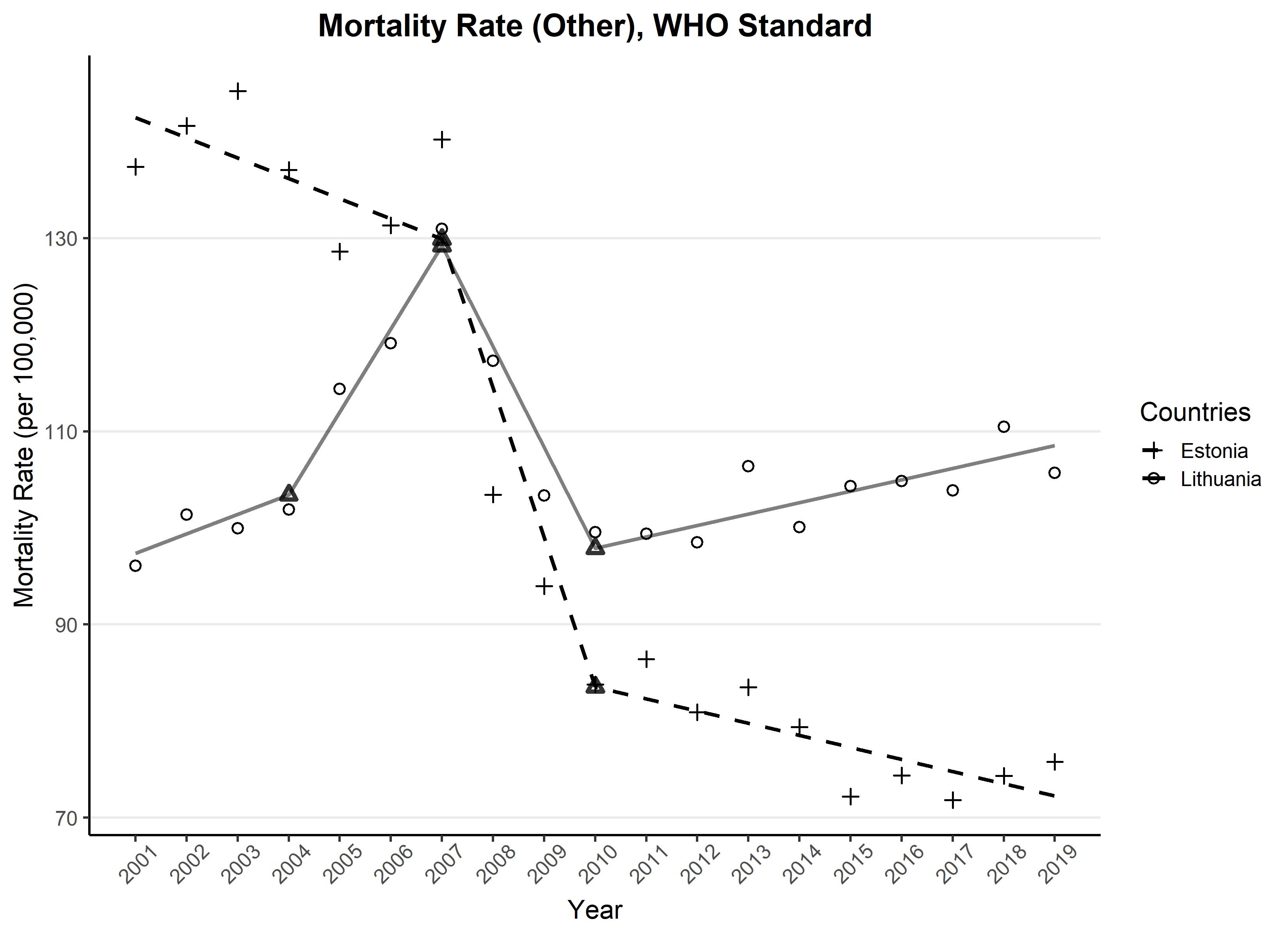


Figure S5. Other causes of death mortality rate (deaths per 100,000 individuals, 20+ years of age) based on EU standard (Graph A) and WHO standard (Graph B), between 2001 and 2019.


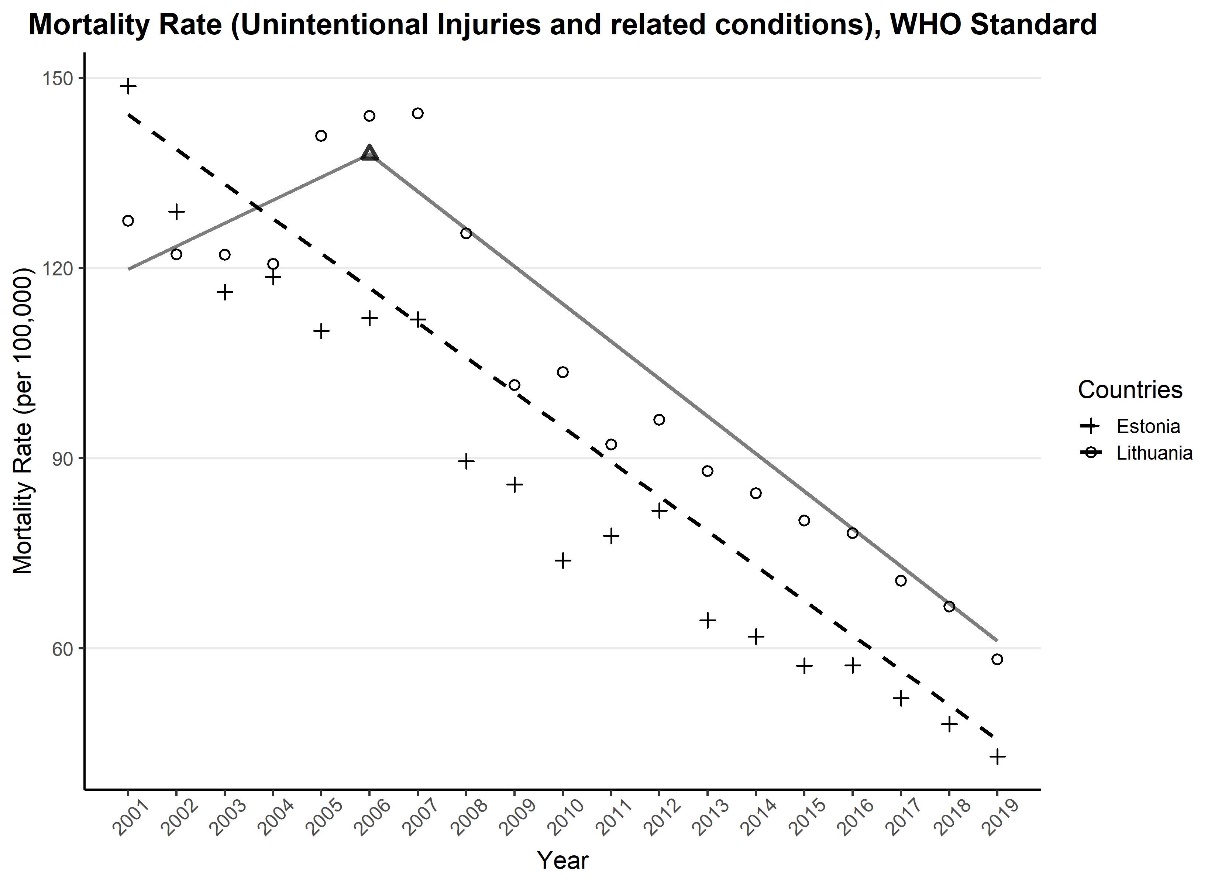


**A**

**B**


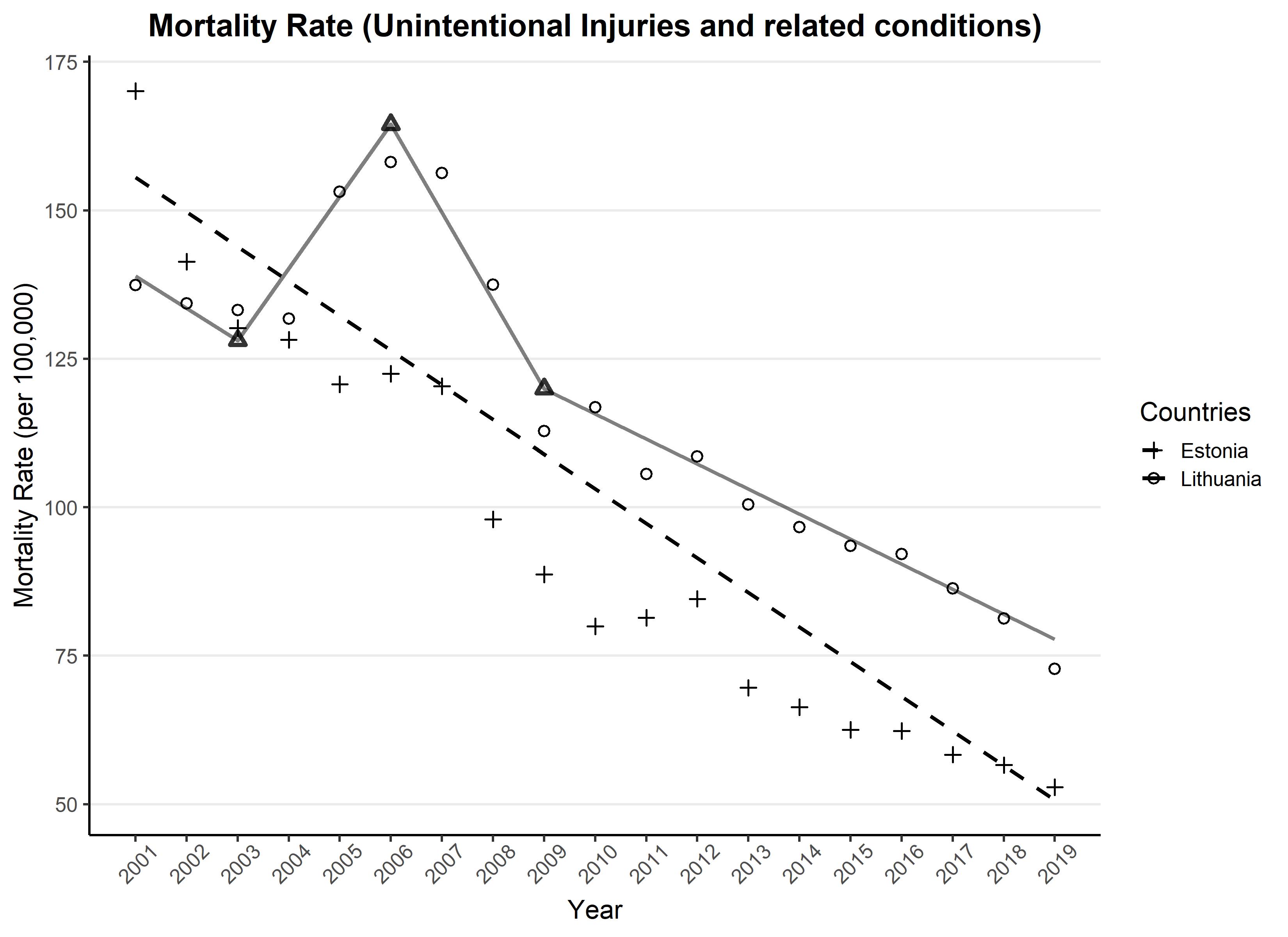


Figure S6. Unintentional injuries and related conditions mortality rate (deaths per 100,000 individuals, 20+ years of age) based on EU standard (Graph A) and WHO standard (Graph B), between 2001 and 2019.


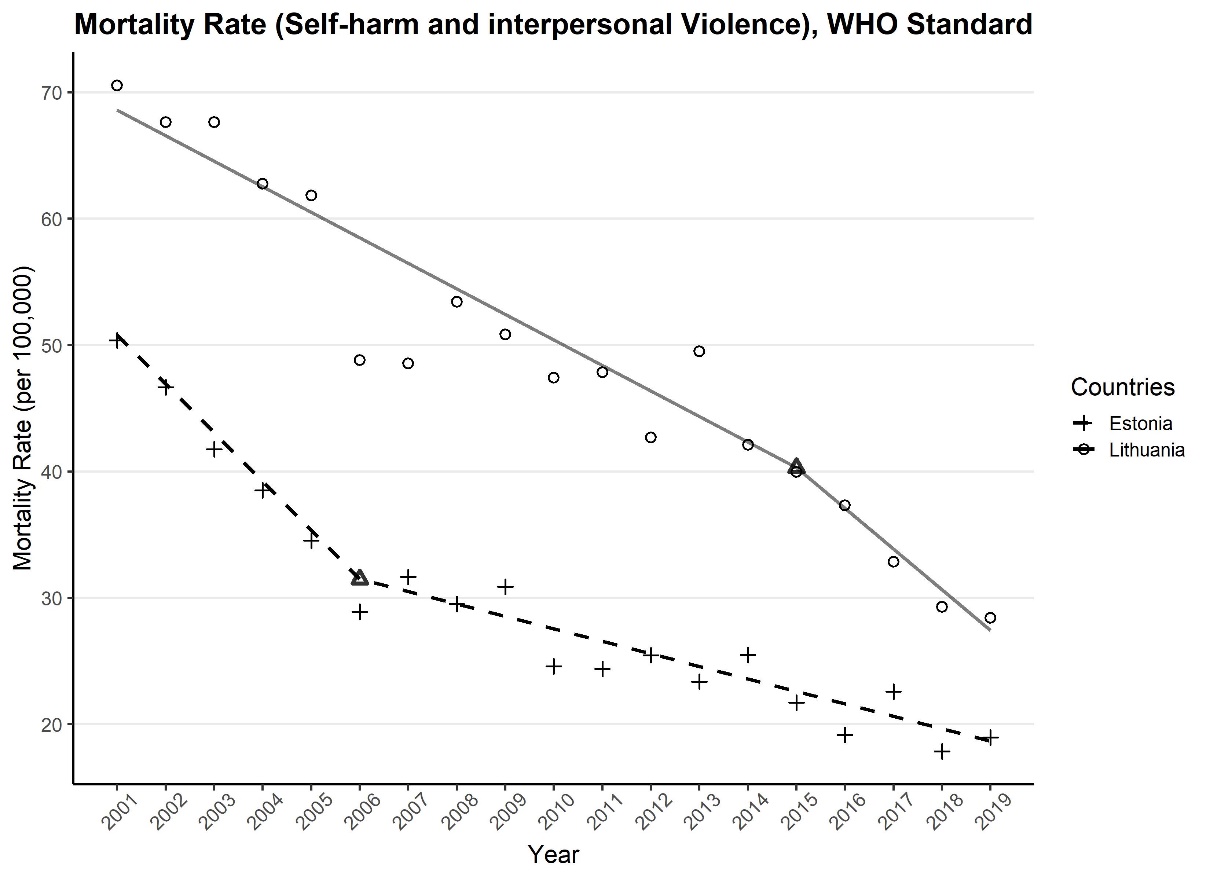


**B**

**A**


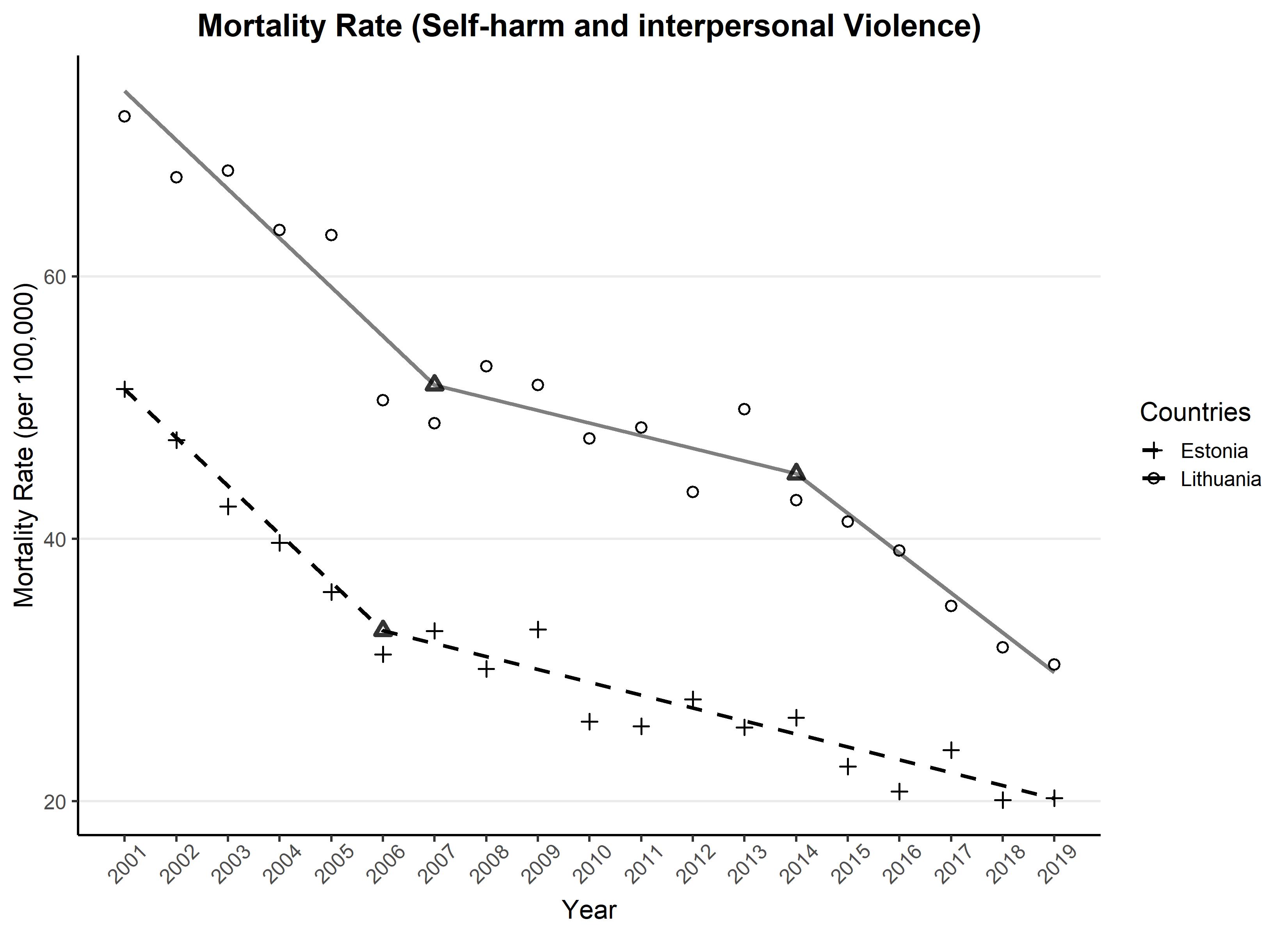


Figure S7. Self-harm and interpersonal violence mortality rate (deaths per 100,000 individuals, 20+ years of age) based on EU standard (Graph A) and WHO standard (Graph B), between 2001 and 2019.


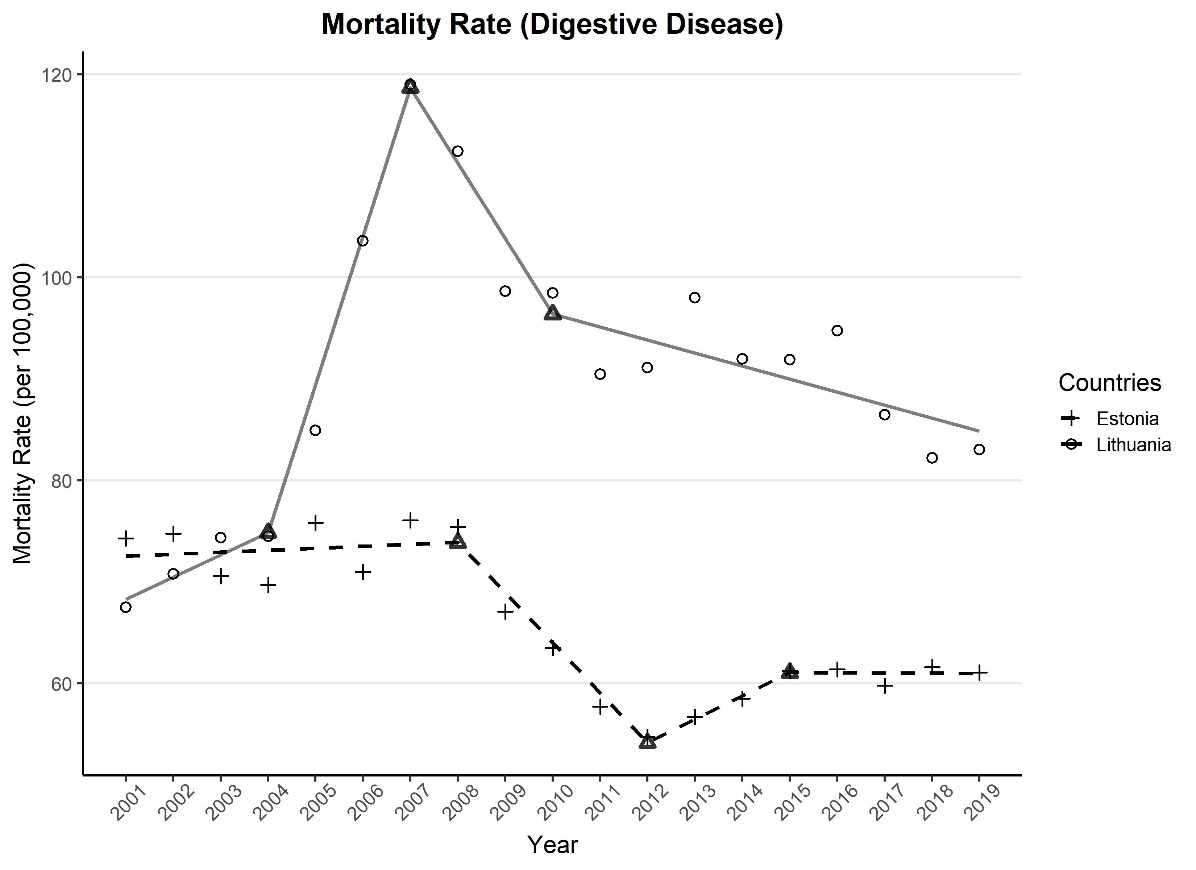


**A**

**B**


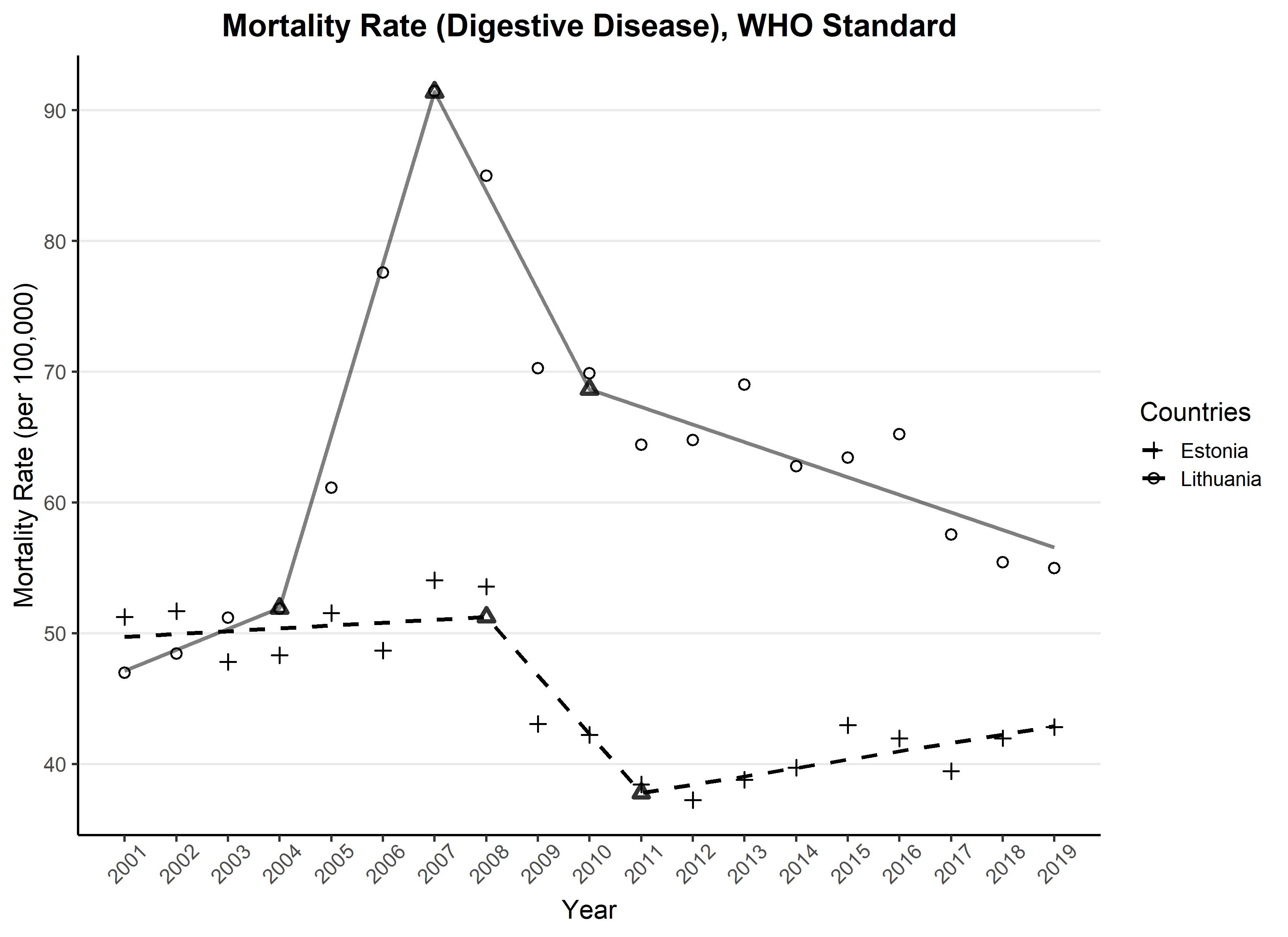


Figure S8. Digestive disease mortality rate (deaths per 100,000 individuals, 20+ years of age) based on EU standard (Graph A) and WHO standard (Graph B), between 2001 and 2019.

**A**


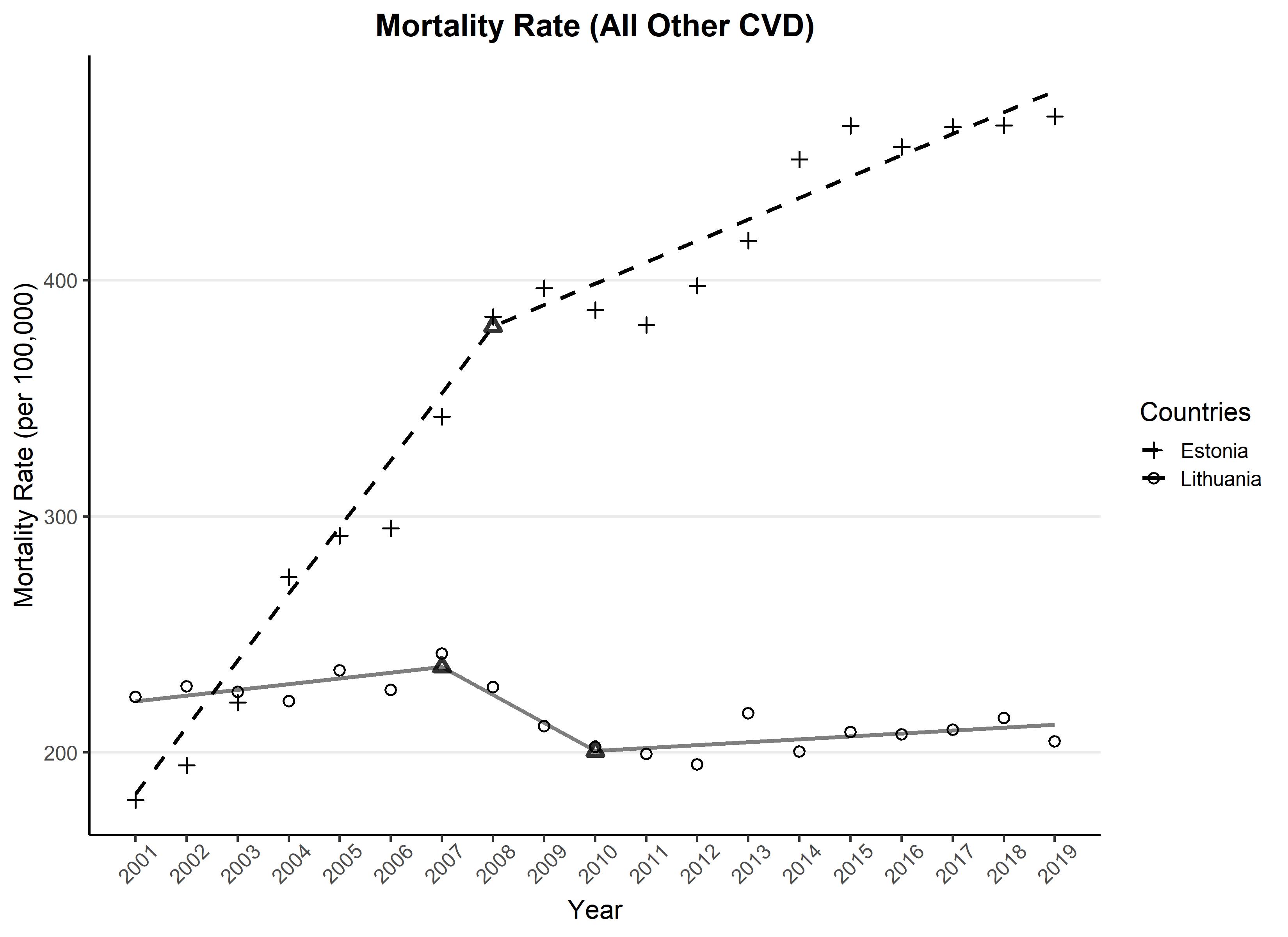


**B**


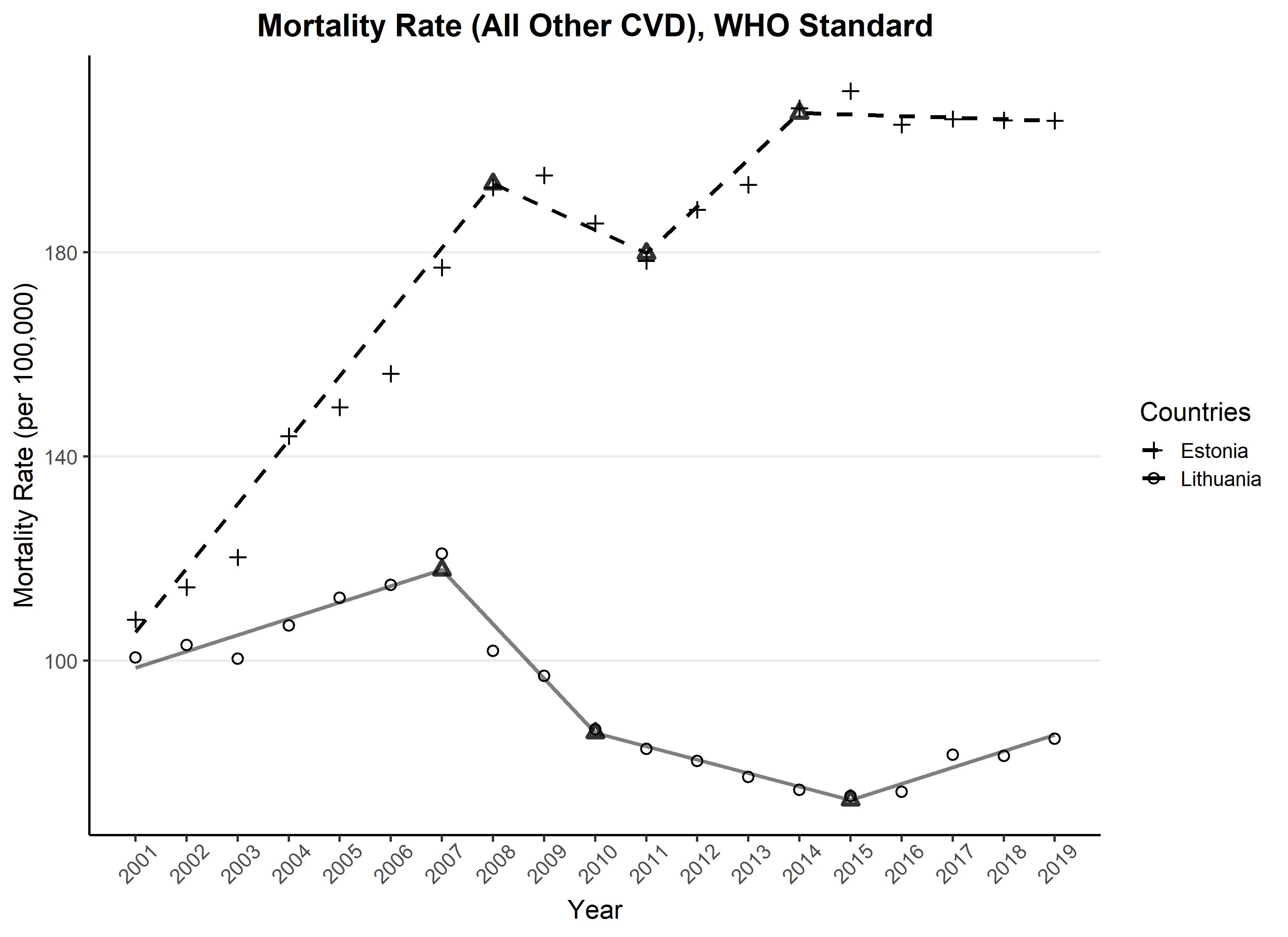


Figure S9. All other cardiovascular disease mortality rate (deaths per 100,000 individuals, 20+ years of age) based on EU standard (Graph A) and WHO standard (Graph B), between 2001 and 2019.


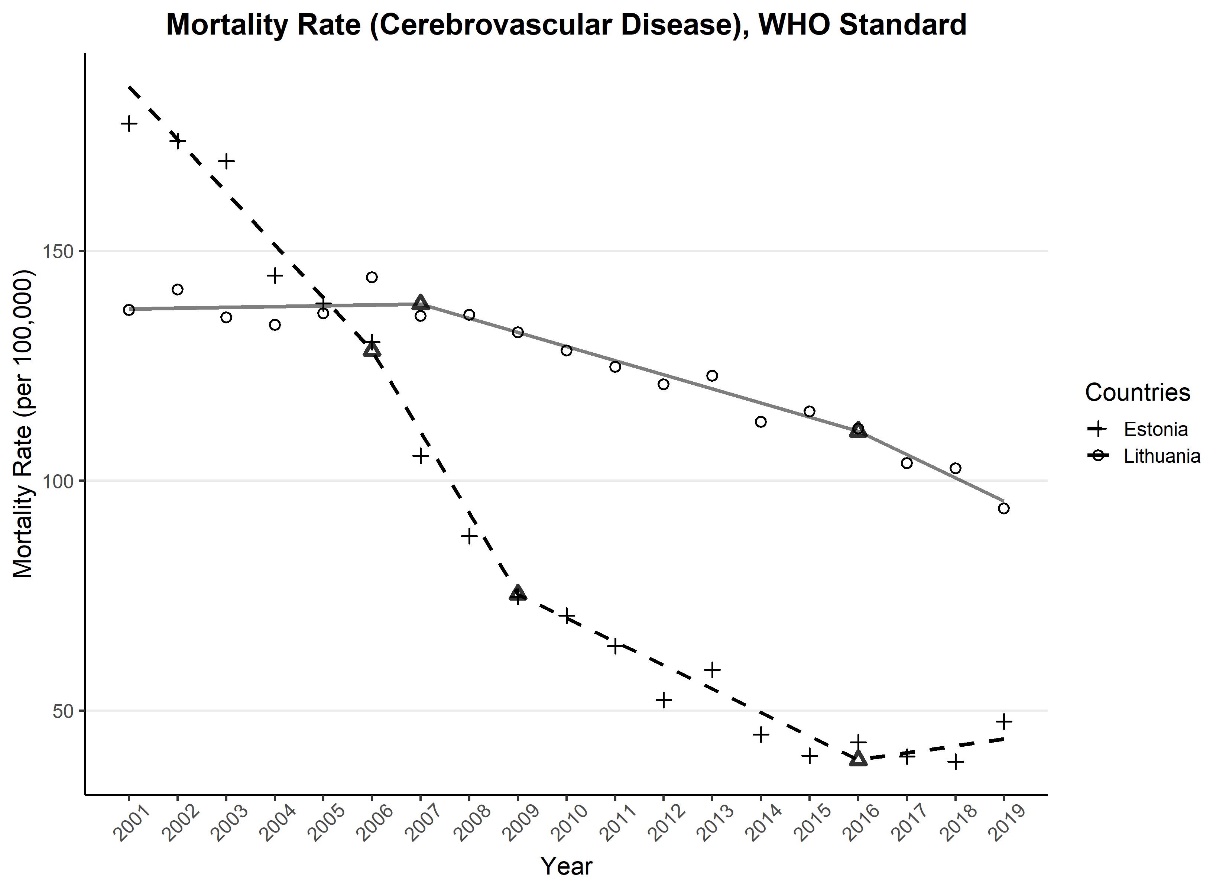


**A**


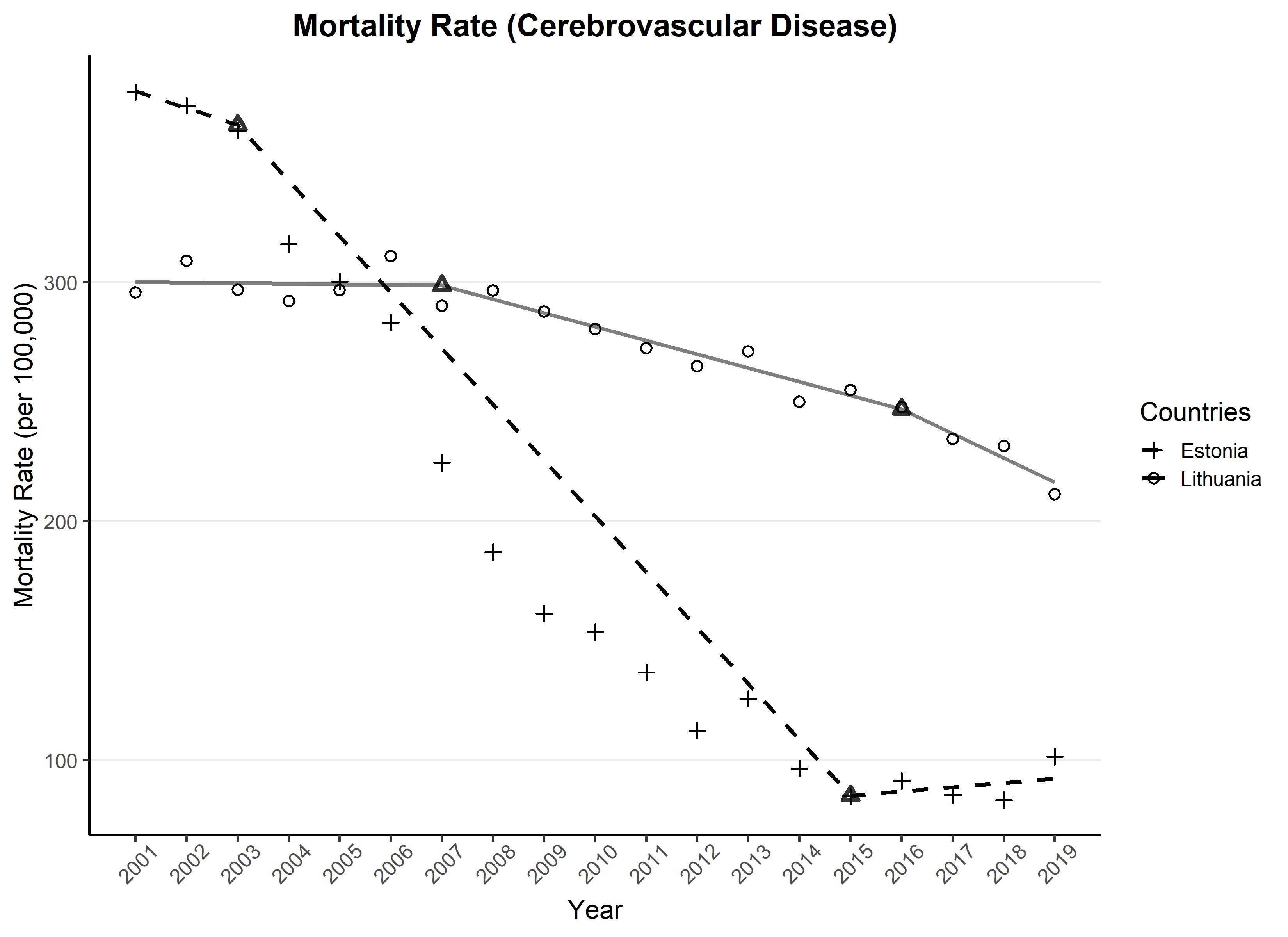


**B**

Figure S10. Cerebrovascular disease mortality rate (deaths per 100,000 individuals, 20+ years of age) based on EU standard (Graph A) and WHO standard (Graph B), between 2001 and 2019.

**A**


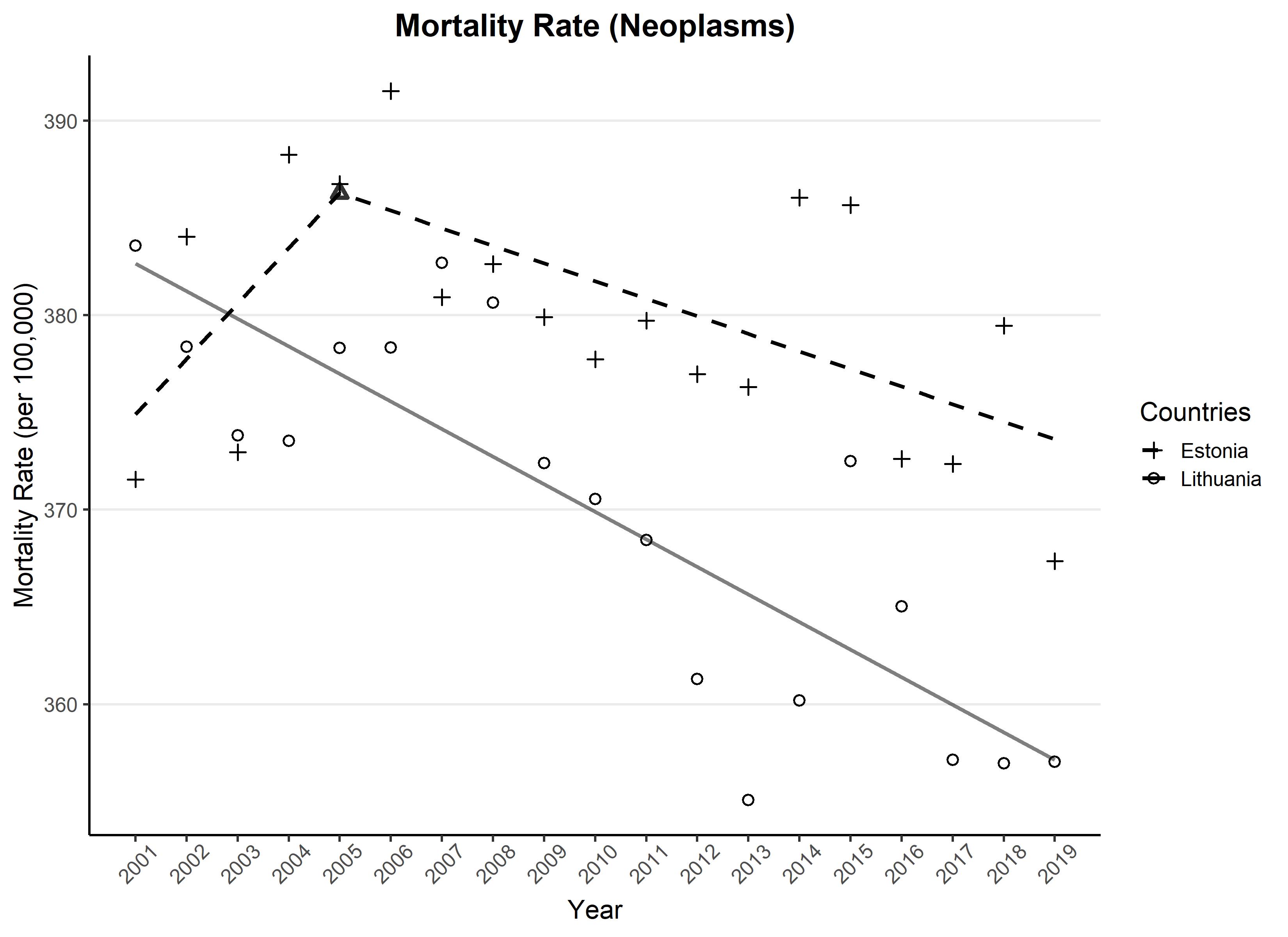


**B**


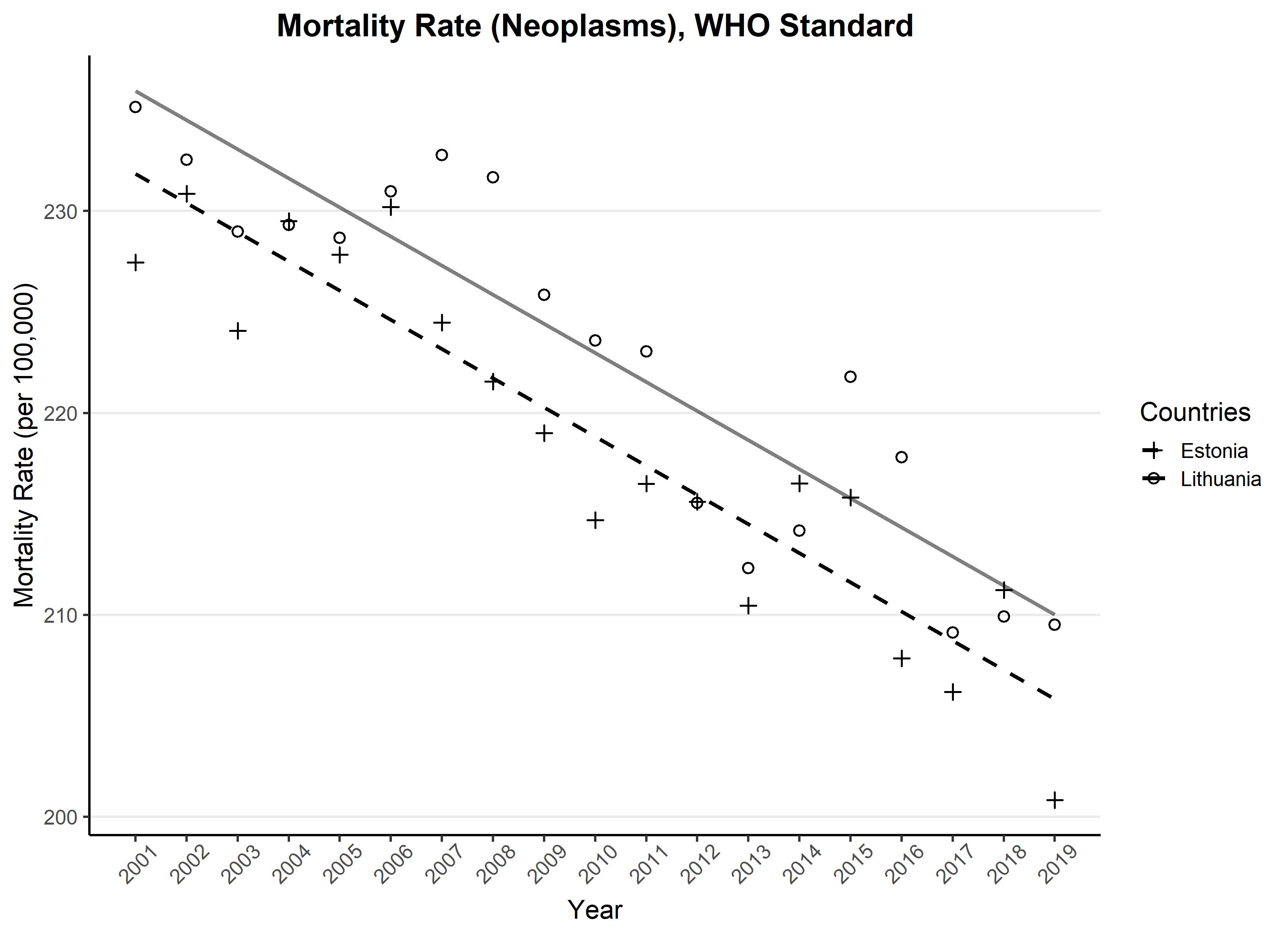


Figure S11. Neoplasm mortality rate (deaths per 100,000 individuals, 20+ years of age) based on EU standard (Graph A) and WHO standard (Graph B), between 2001 and 2019.


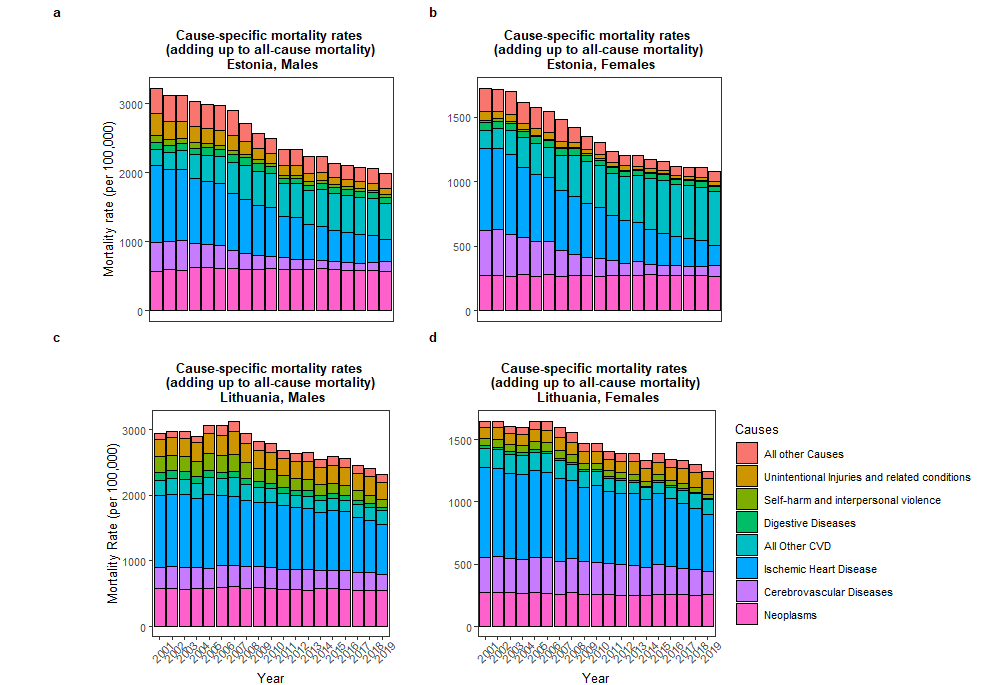
Figure S12. Mortality rate (deaths per 100,000 individuals, 20+ years of age) in Estonia (Graph a and b) and Lithuania (Graph c and d), separated by sexes and causes between 2001 and 2019 according to EU standard. Mortality rate in each year sums to all-cause mortality.


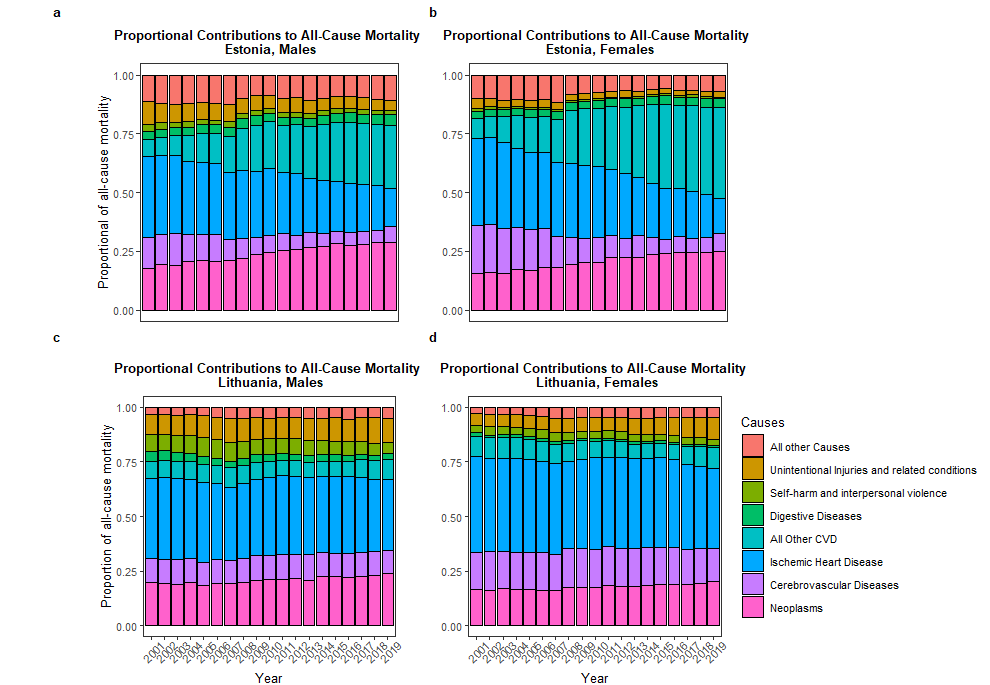
Figure S13. Mortality rate (deaths per 100,000 individuals, 20+ years of age) in Estonia (Graph a and b) and Lithuania (Graph c and d), separated by sexes and causes between 2001 and 2019 according to EU standard. Mortality rate in each year sums to all-cause mortality.


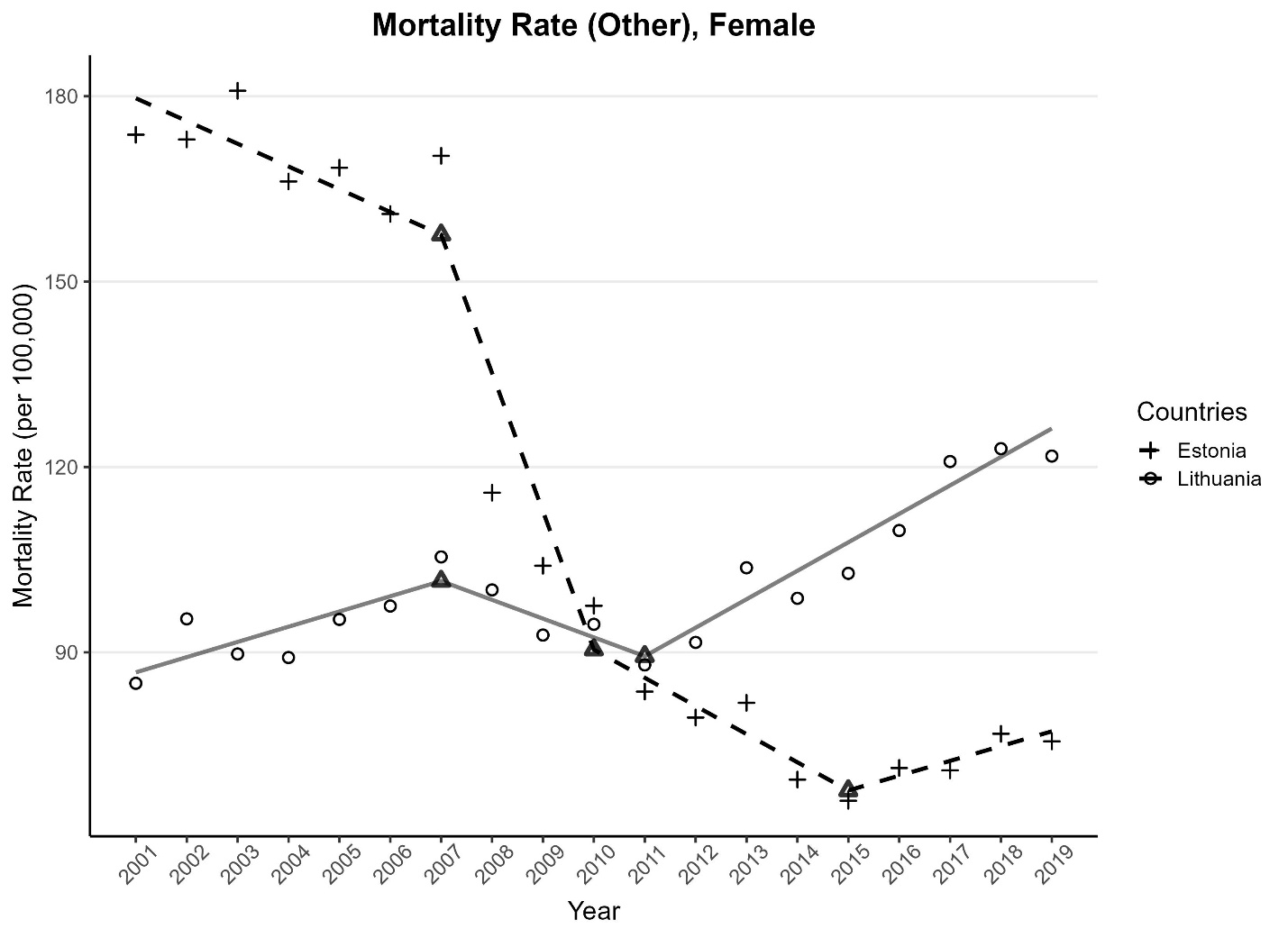


Figure S14. Other mortality rate (deaths per 100,000 individuals, 20+ years of age) for females, between 2001 and 2019.


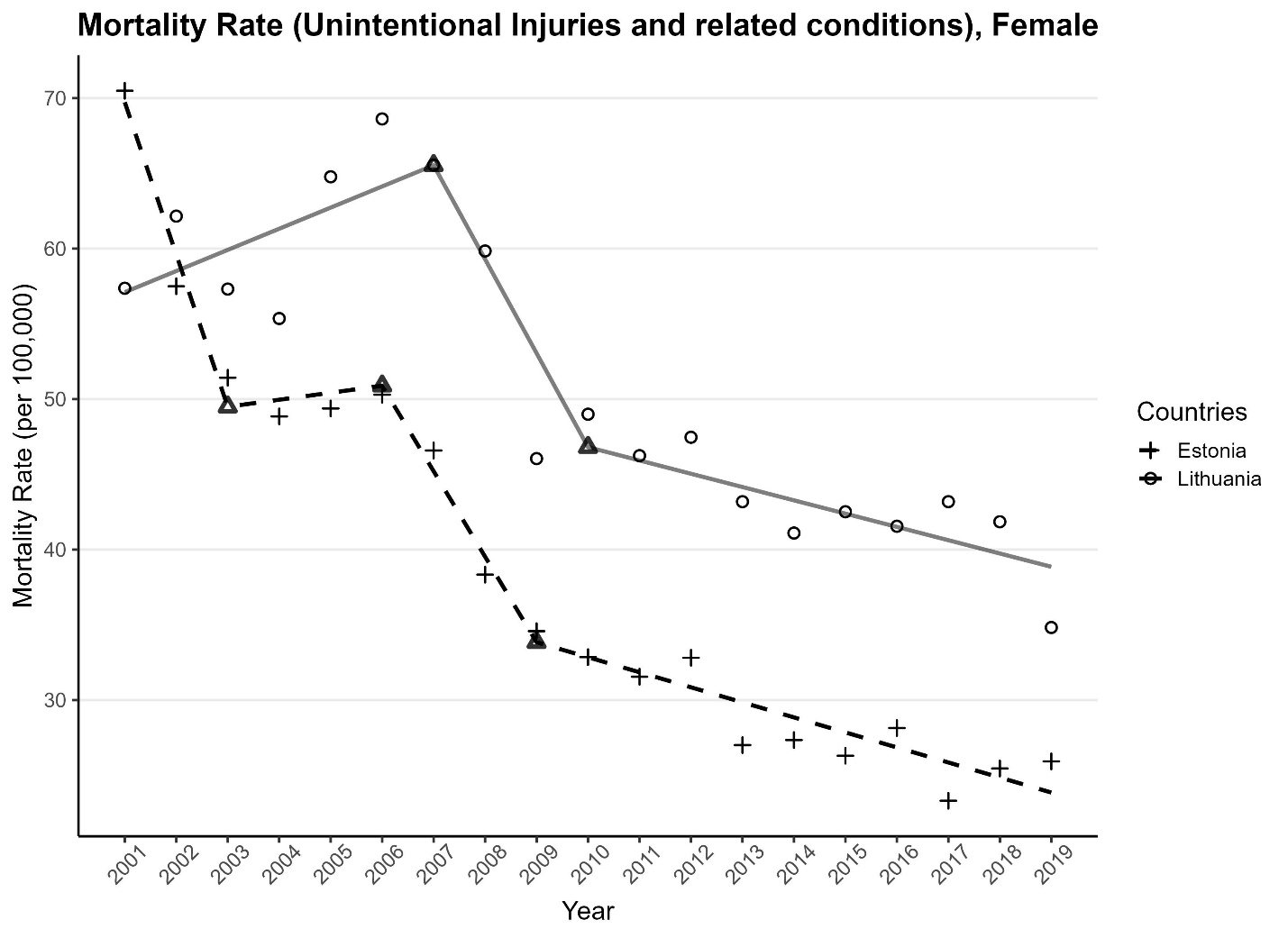
 Figure S15. Unintentional injuries and related conditions mortality rate (deaths per 100,000 individuals, 20+ years of age) for females, between 2001 and 2019.

Figure S16. Self-harm and interpersonal violence mortality rate (deaths per 100,000 individuals, 20+ years of age) for females, between 2001 and 2019.
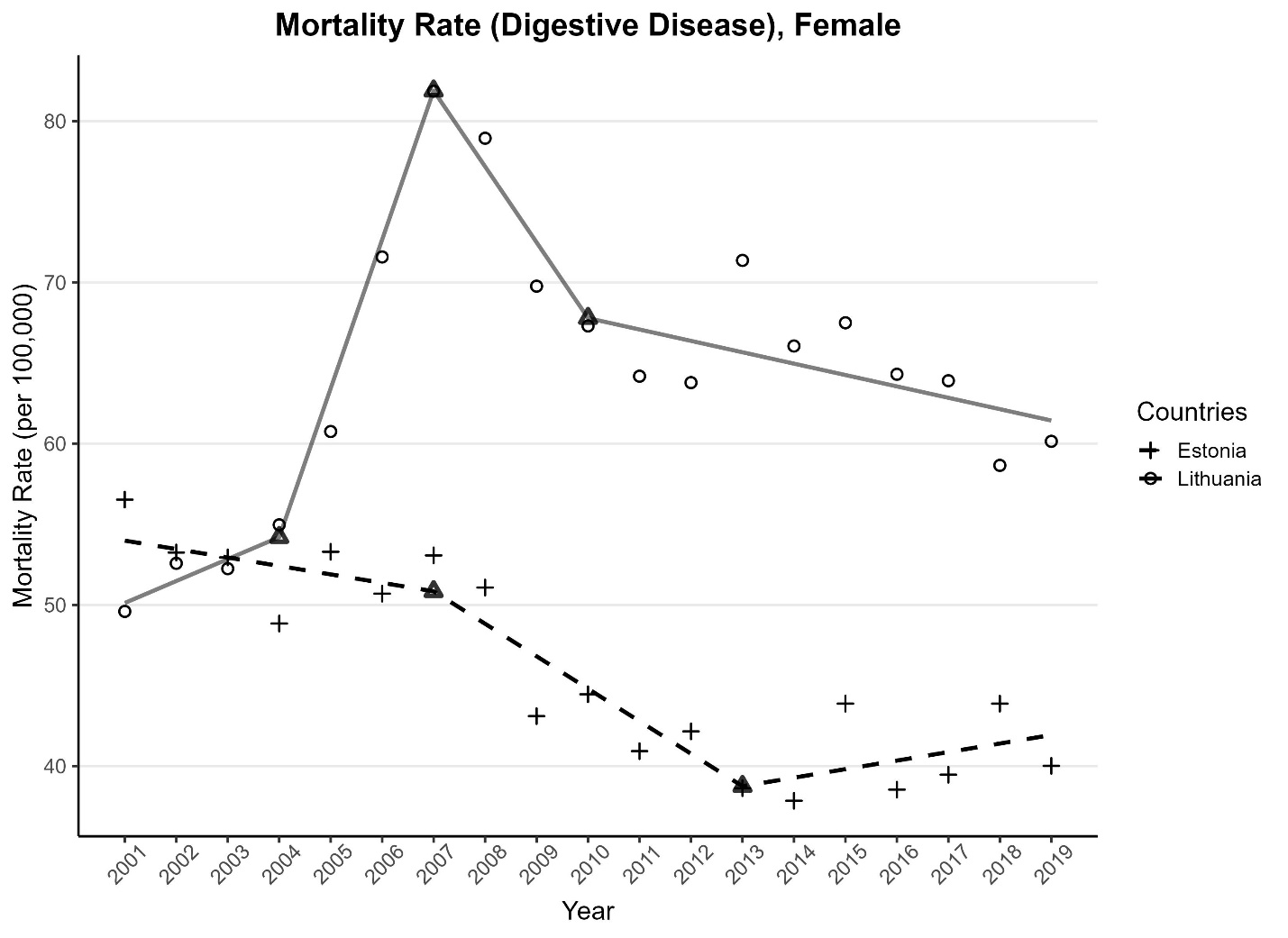
 Figure S17. Digestive disease mortality rate (deaths per 100,000 individuals, 20+ years of age) for females, between 2001 and 2019. Figure S18. All other cardiovascular disease mortality rate (deaths per 100,000 individuals, 20+ years of age) for females, between 2001 and 2019 Figure S19. Cerebrovascular disease mortality rate (deaths per 100,000 individuals, 20+ years of age) for females, between 2001 and 2019.


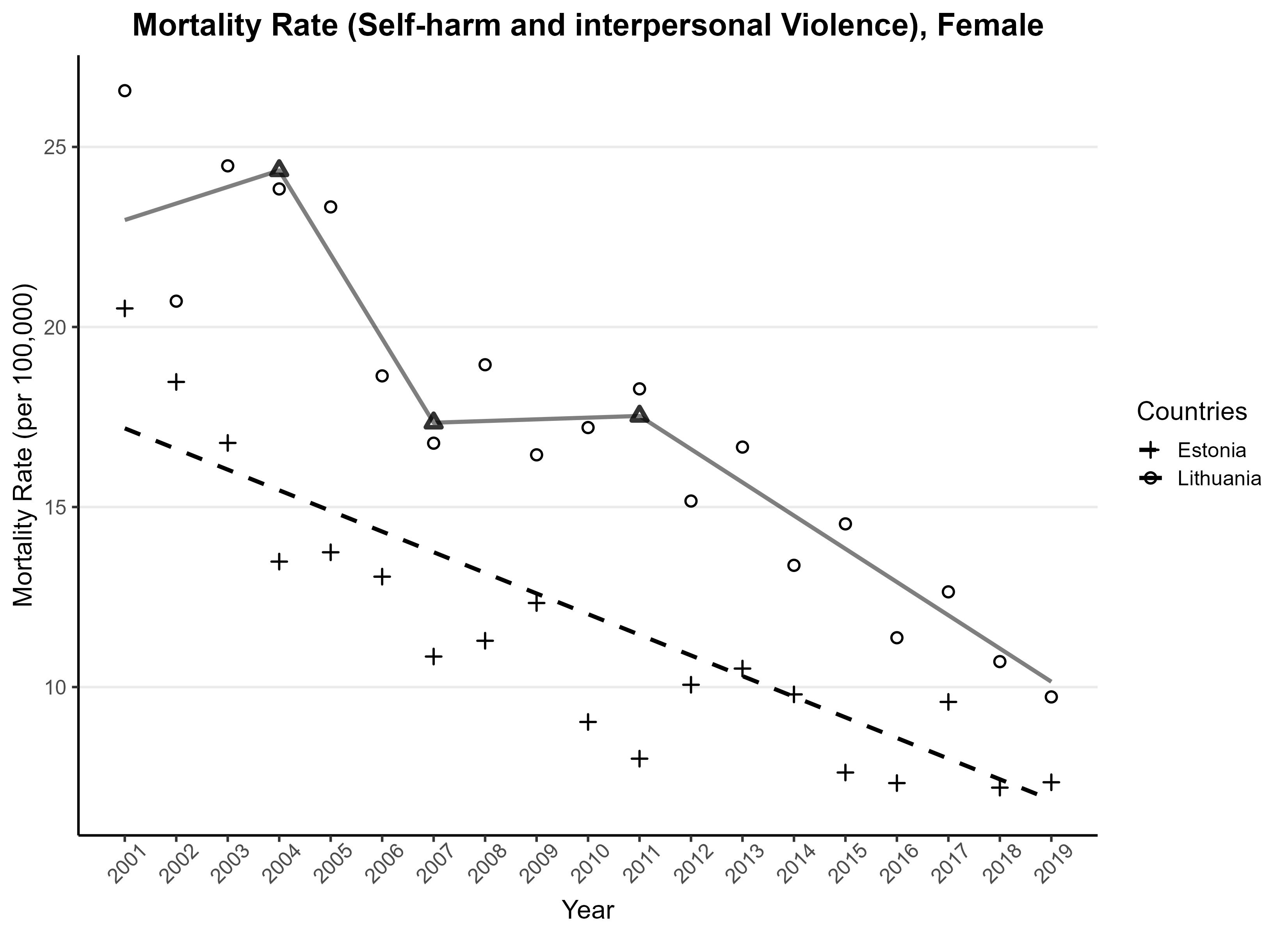

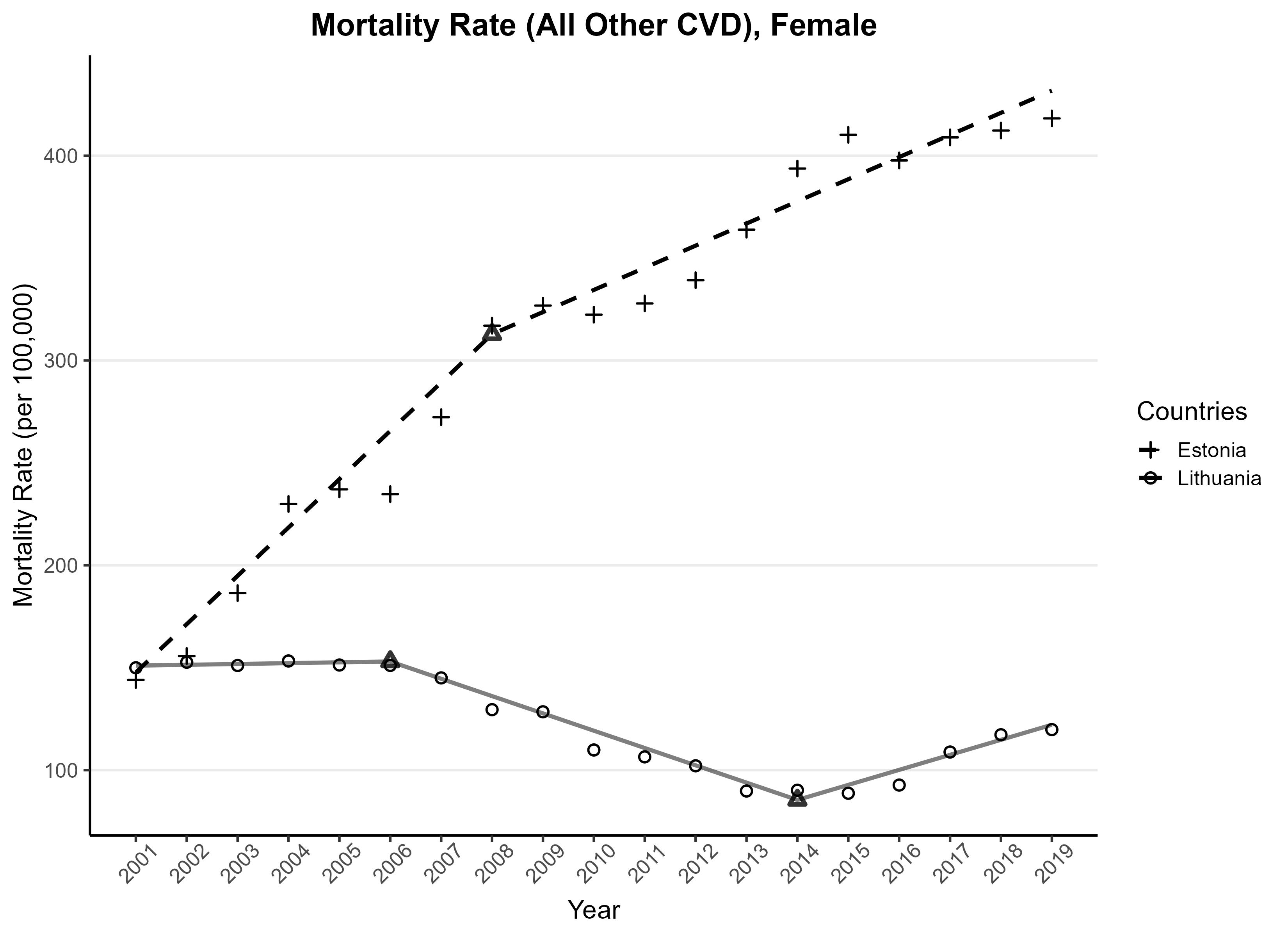

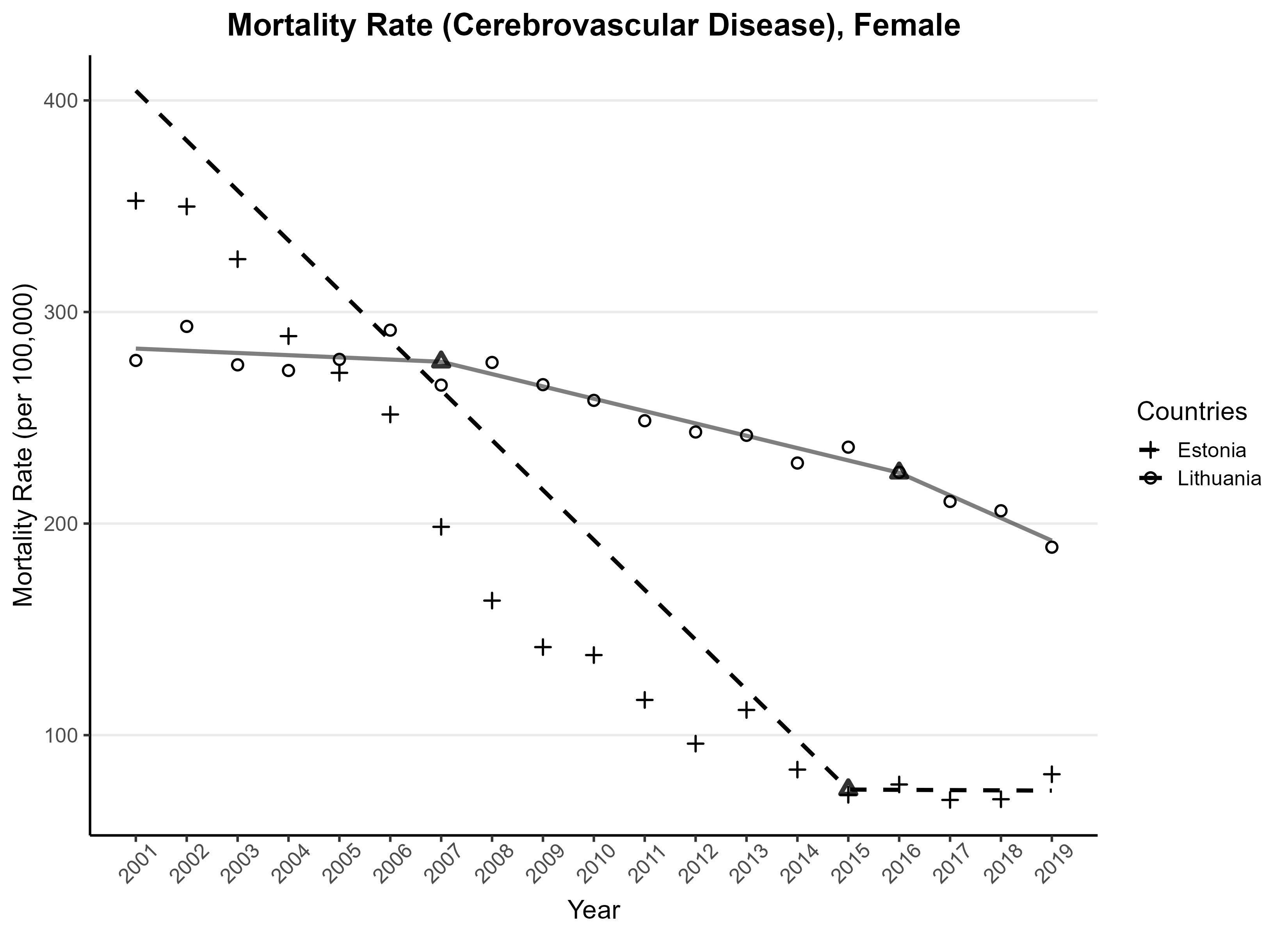

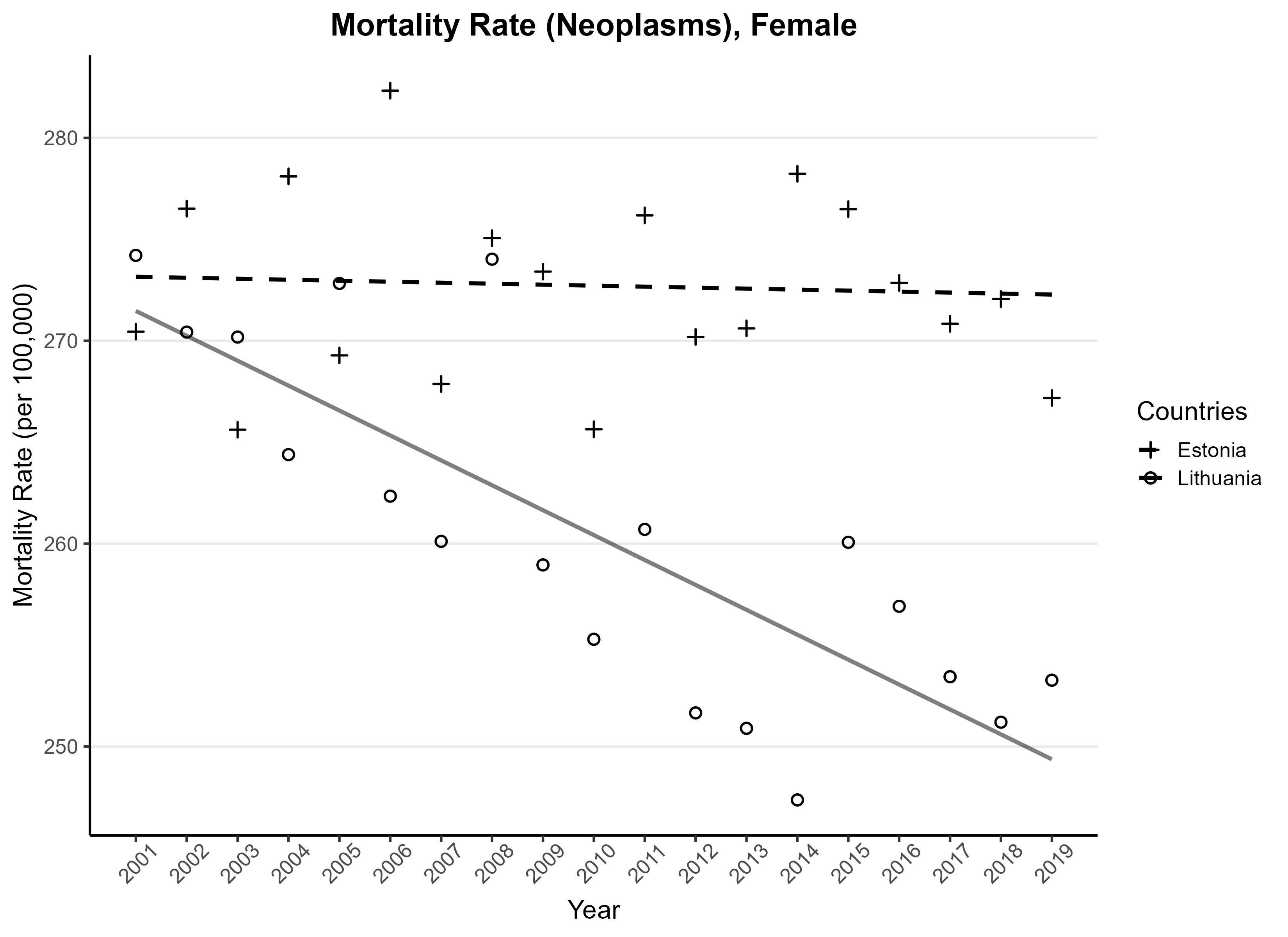


Figure S20. Neoplasm mortality rate (deaths per 100,000 individuals, 20+ years of age) for females, between 2001 and 2019.


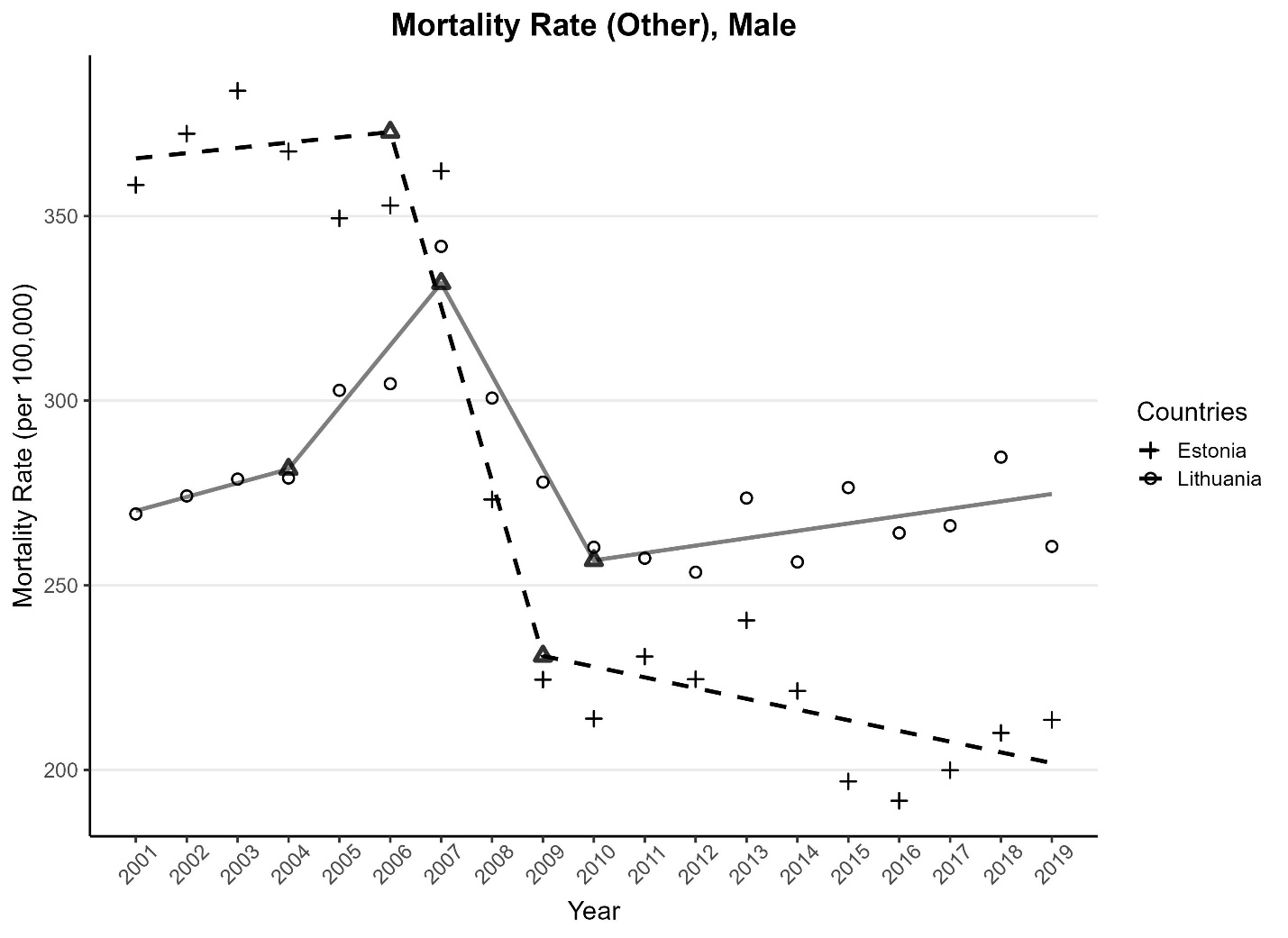
 Figure S21. Other mortality rate (deaths per 100,000 individuals, 20+ years of age) for males, between 2001 and 2019. Figure S22. Unintentional injuries and related conditions, mortality rate (deaths per 100,000 individuals, 20+ years of age) for males, between 2001 and 2019.
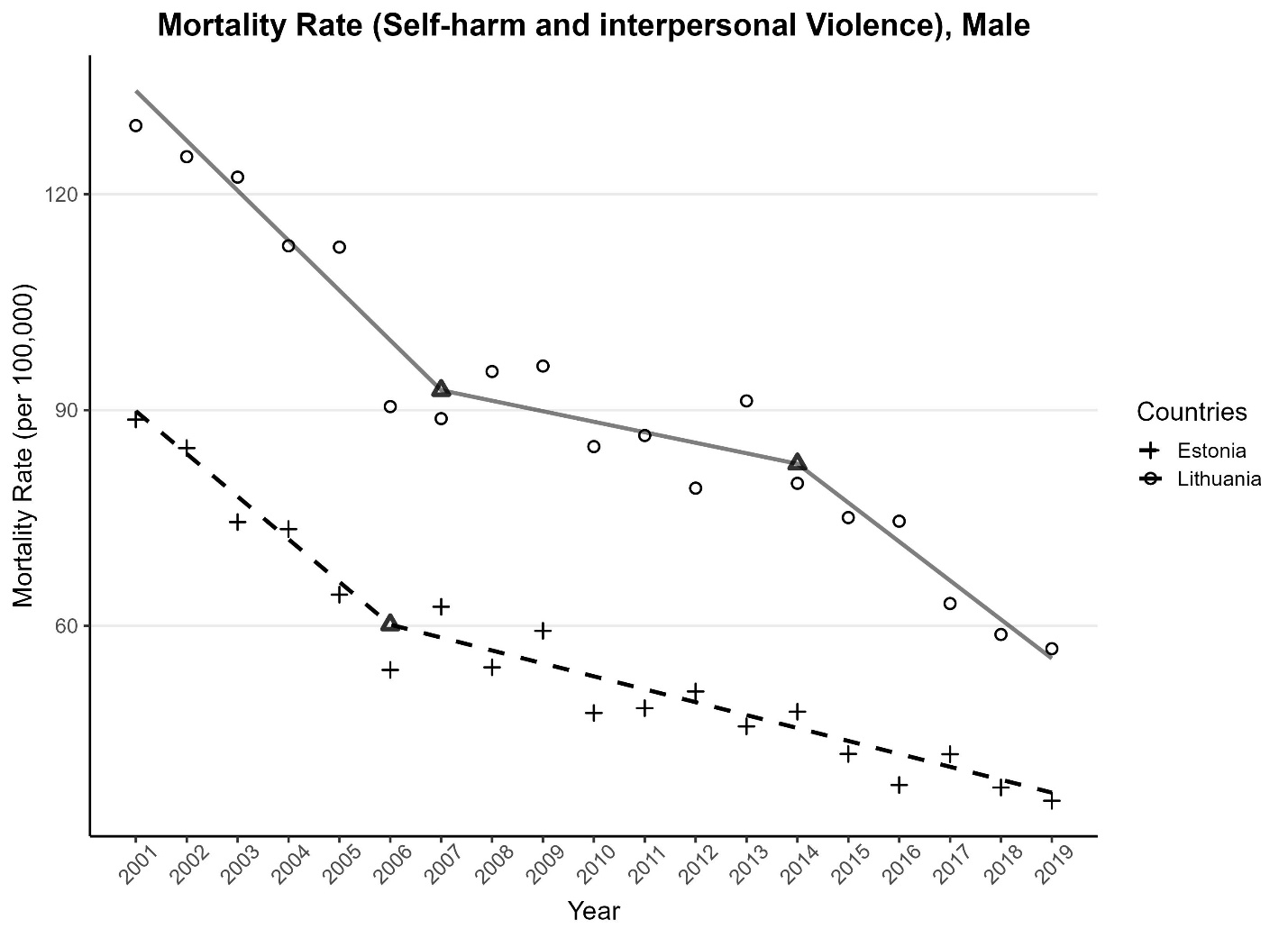
 Figure S23. Self-harm and interpersonal violence mortality rate (deaths per 100,000 individuals, 20+ years of age) for males, between 2001 and 2019.
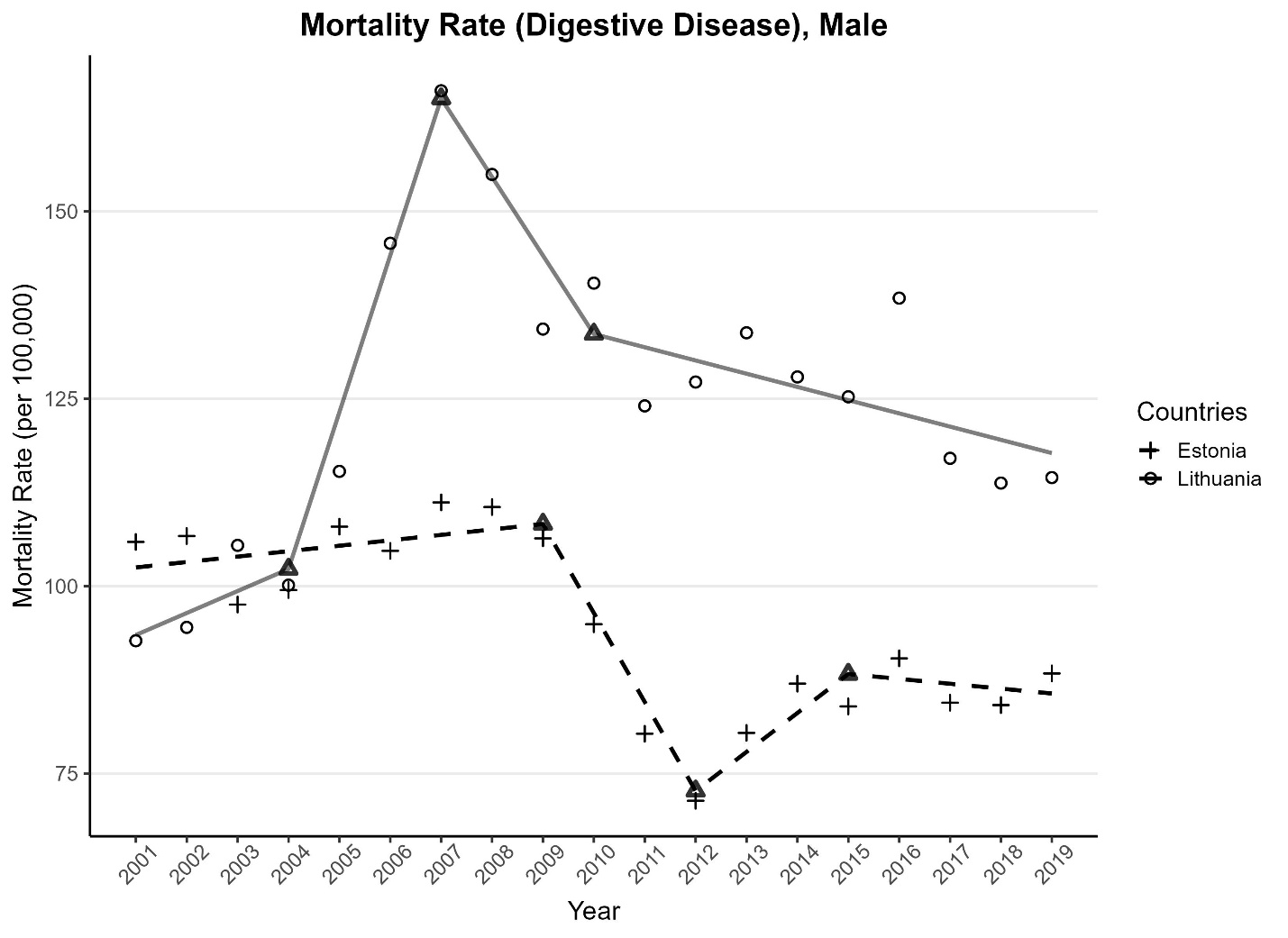
 Figure S24. Digestive disease mortality rate (deaths per 100,000 individuals, 20+ years of age) for males, between 2001 and 2019.
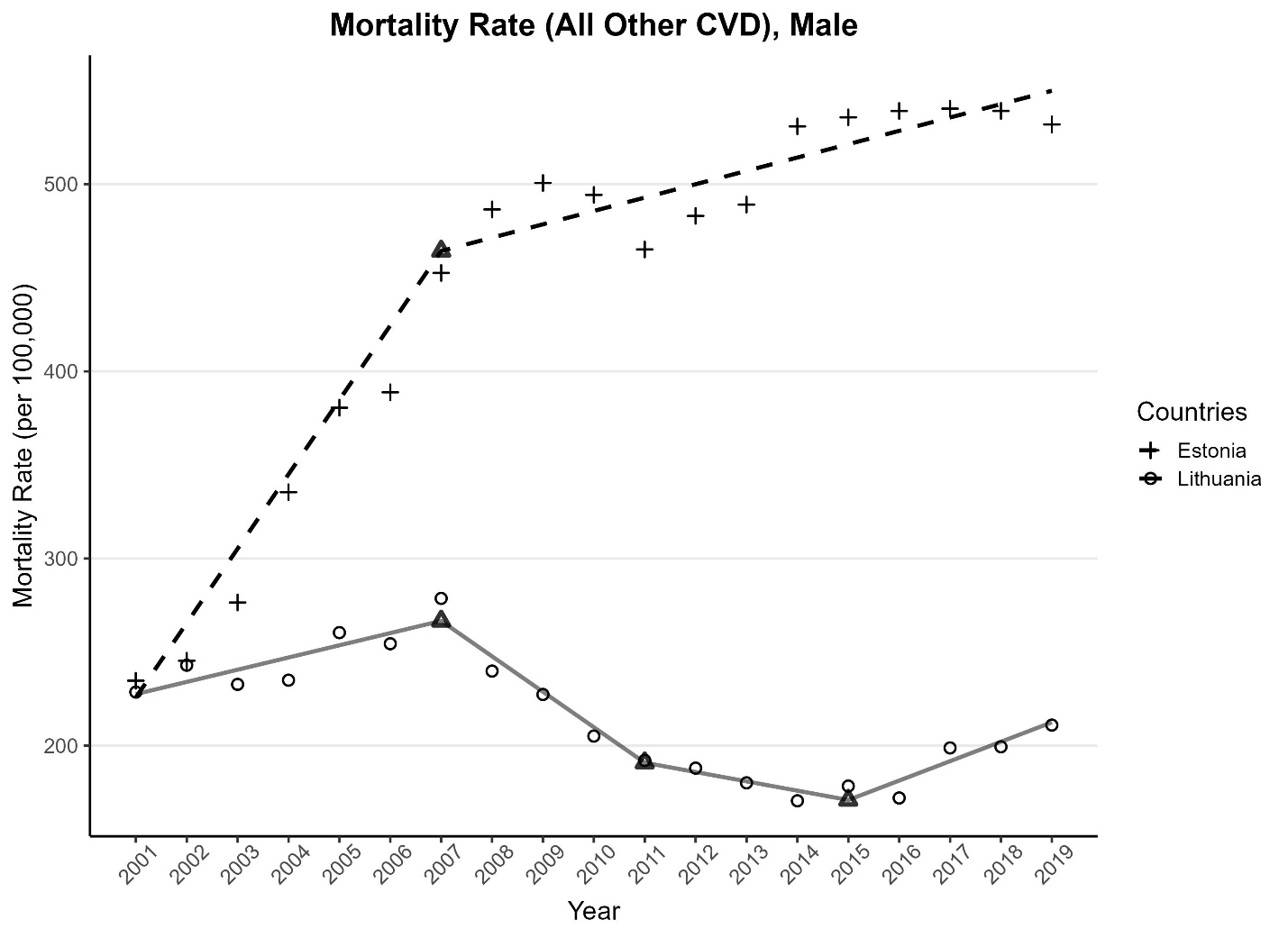
 Figure S25. All other cardiovascular disease mortality rate (deaths per 100,000 individuals, 20+ years of age) for males, between 2001 and 2019. Figure S26. Cerebrovascular disease mortality rate (deaths per 100,000 individuals, 20+ years of age) for males, between 2001 and 2019. Figure S27. Neoplasm mortality rate (deaths per 100,000 individuals, 20+ years of age) for males, between 2001 and 2019.


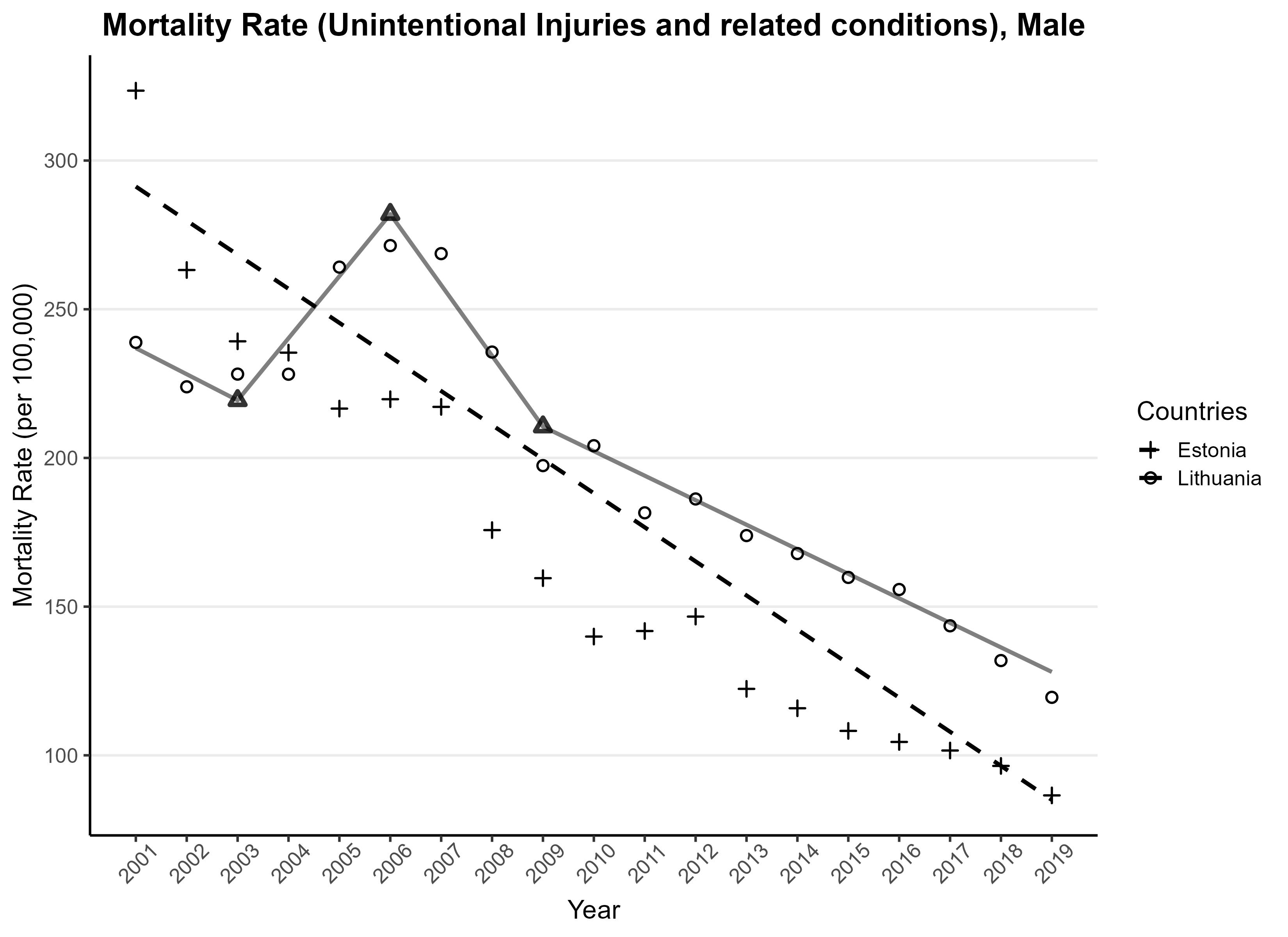

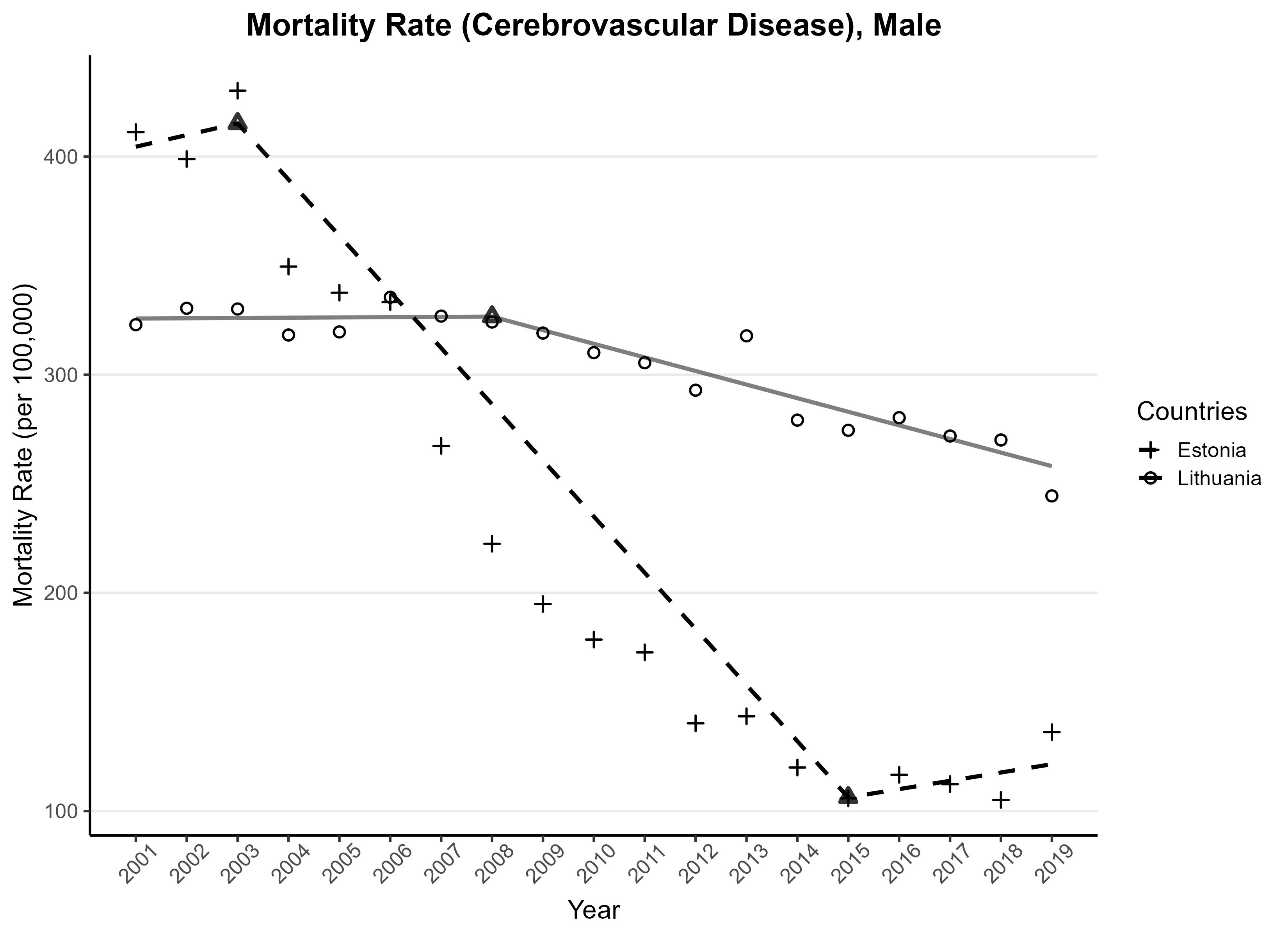

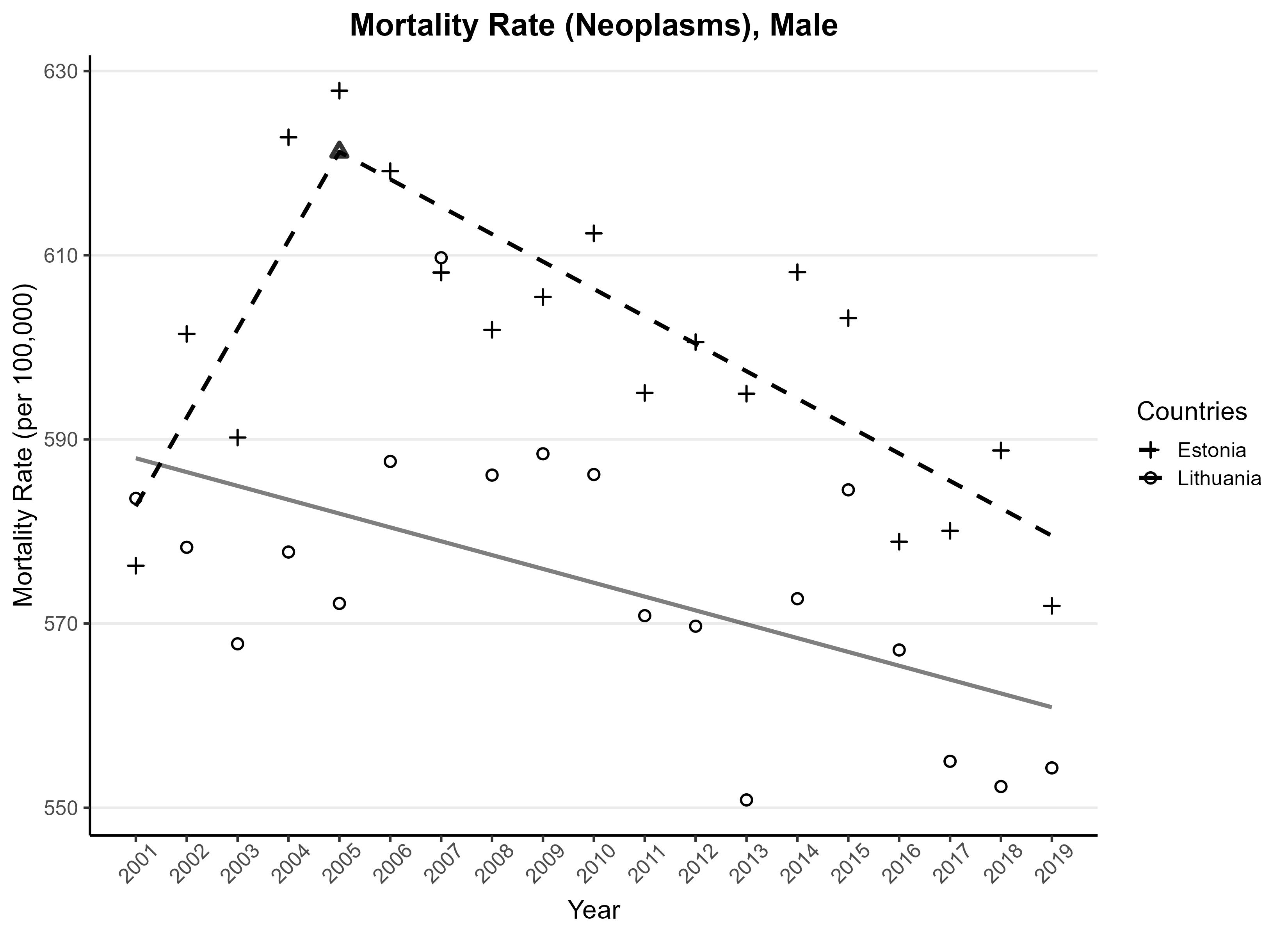

Supplement: Supplementary file 1 — Additional file 1. [file 12889_2022_14354_MOESM1_ESM.docx]
